# Exploring the Anticancer Potential of Plant, Marine, and Synthetic Glycosides: A Comprehensive Review

**DOI:** 10.3390/biom16101493

**Published:** 2026-10-14

**Authors:** Tooba Jabeen, Sumayya Akram, Matloob Ahmad, Sami A. Al-Hussain, Magdi E. A. Zaki

**Affiliations:** 1 Department of Chemistry, Government College University, Faisalabad 38000, Pakistan; toobajabeen65@gmail.com (T.J.); sumayya.201002384@gcuf.edu.pk (S.A.); 2 Department of Chemistry, Faculty of Science, Imam Mohammad Ibn Saud Islamic University (IMSIU), Riyadh 11623, Saudi Arabia; mezaki@imamu.edu.sa

**Keywords:** glycoside, anticancer, marine, plant, synthetic

## Abstract

Despite advances in pharmaceutical and technological development, cancer continues to be a life-threatening illness globally. In addition to other specialized procedures, radiotherapy, chemotherapy, and surgery are methods used to treat cancer. However, in recent years, natural products have either been used as food supplements or complementary medicine in the management and treatment of cancer. Natural products are the secondary metabolites present in the cells of medicinal plants, microbes, and marine organisms. Glycosides are a naturally occurring class of metabolites with exceptional therapeutic value and clinical efficacy. The structure of all glycosides is similar; it consists of two parts: a hydrophilic glycone unit made up of one or more sugar components, and a lipophilic aglycone unit. Over the past few years, the potential anti-cancer properties of several natural and synthetic glycosides have been investigated. In this review, we compile and analyze **224** naturally occurring glycosides from plant and marine sources, along with more than **40** synthetic glycosides, that exhibit anticancer properties. Reported anticancer activities span a wide potency range and involve multiple mechanisms, including apoptosis, cell-cycle arrest, oxidative stress, mitochondrial dysfunction, inhibition of PI3K/AKT, MAPK, STAT3, Wnt/β-catenin, and Na^+^/K^+^-ATPase signaling, as well as suppression of migration and invasion. Several compounds show potency comparable to or exceeding standard chemotherapeutics under matched assay conditions: for example, an epigallocatechin-3-*O*-gallate derivative inhibited MDA-MB-231 cells at an IC_50_ of 3.27 ± 0.18 µM versus 5.20 ± 0.89 µM for capecitabine, and a synthetic N-glycosyl heterocycle (compound **479a**) outperformed erlotinib against HepG-2 and HCT-116 cells (IC_50_ = 1.64 ± 0.11 and 5.00 ± 0.51 µM vs. 2.07 ± 0.07 and 5.14 ± 0.33 µM). Other studies reported glycosides with activities comparable to or greater than reference agents such as capecitabine, tamoxifen, and erlotinib. Despite these promising findings, the evidence remains predominantly preclinical, with substantial limitations in normal-cell selectivity, standardized cytotoxicity assessment, pharmacokinetic characterization, and in vivo and clinical validation. Overall, glycosides represent promising anticancer scaffolds, but systematic optimization of potency, selectivity, stability, bioavailability, and safety is required to facilitate their translation into clinically useful therapeutics.

## 1. Introduction

Cancer is regarded as one of the diseases that pose the biggest threat to life worldwide [1,2]. While early recognition contributes to a slight increase in overall survival, cancer-related mortality remains the second leading cause of death globally. According to the International Agency for Research on Cancer (IARC), there were 8.2 million deaths attributed to cancer and 14.1 million new cases globally in 2012. By 2030, there will be an estimated 21.7 million new cancer cases and 13 million deaths. Of the various cancer types, the most common malignancies in humans have been identified as lung, breast, colorectal, stomach, prostate, and liver cancers [3].

The rapid growth of drug resistance to cancer, coupled with the significant toxicity and side effects associated with some chemotherapeutic drugs, has caused a greater demand for new anti-cancer medications, especially those derived from natural sources [4]. Natural products (NP, also known as secondary or specialized metabolites) are found in the cells and tissues of microbes, fungi, plants, and animals from both aquatic and terrestrial habitats. They have been utilized for medicinal purposes since ancient times and are crucial components of modern pharmacology [5,6,7,8,9,10,11]. At present, almost 60% of drugs approved for the treatment of cancer are derived from natural sources [12,13]. Natural substances that are used to treat and mitigate cancer conditions can have an impact on biological pathways such as the cell cycle and proliferation, cancer cell migration and invasion, apoptosis, autophagy, and angiogenesis [14,15].

Among the myriad compounds isolated from natural sources, glycosides have attracted considerable attention because of their wide range of biological activities and potential anticancer effects. Glycosides arise from a variety of sources, including plants, marine organisms, and synthetic processes. They are distinguished by the glycosidic connections that they form between a glycone unit and a non-sugar component. The literature indicates that many glycosides extracted from various plants and marine environments exhibit significant anticancer activity against diverse cancer cell types (Figure 1). Moreover, various synthetic approaches have been utilized to afford a wide range of anticancer glycosides.

A comprehensive evaluation of the therapeutic potential of synthetic glycosides and semisynthetic derivatives has now been expanded by the rapid growth of research in this area. Unlike previous reviews, which have each focused on a single subclass or source, for example, cardiac glycosides, saponins, flavonoid glycosides, or marine-derived metabolites [16,17,18,19,20,21], this review is distinguished by its simultaneous coverage of plant-, marine-, and synthetic-source anticancer glycosides within a single framework, spanning the literature from 2000 to 2025. It further incorporates synthetic methodology alongside natural-product pharmacology, and includes a dedicated critical appraisal of methodological quality across the included studies (Section 5 that is largely absent from the narrower reviews cited above. In this way, the present work is intended to complement rather than supersede these earlier reviews by mapping the anticancer glycoside literature across sources, mechanisms, and synthetic strategies in one place.

### Literature Search Strategy and Selection Criteria

A systematic literature search was carried out to find articles on anticancer-based plant, marine, and synthetic glycosides and aptasensors from 2000 to 2025. The following electronic databases were searched: Web of Science, Scopus, PubMed, ScienceDirect, ACS Publications, and Google Scholar. The keywords used included: anticancer-based plant glycosides, anticancer-based anticancer glycosides, anticancer-based marine glycosides, anticancer-based cardiac glycosides, anticancer-based synthetic glycosides, saponins, cytotoxic glycosides, flavonoid glycosides, and glycoside-based cancer therapy. The inclusion criteria include studies reporting in vitro, in vivo, mechanistic, or computational evidence of anticancer activity of plant, marine, and synthetic glycosides, as well as English-language articles. The exclusion criteria were studies that did not include anticancer activity of glycosides, lacked sufficient biological evidence, conference abstracts, patents, non-peer-reviewed reports without full texts, and non-English-language articles. A PRISMA flowchart summarizing the literature search and selection process is presented in Figure 2.

## 2. Plant-Derived Glycosides

### 2.1. Steroid Glycosides

In the past, steroid glycosides have been utilized to treat malignant diseases, arrhythmias, and congestive heart failure [22,23,24,25]. These are substances isolated from plants or animals. Chemically, these glycosides are classified into two groups: cardenolides, identified by an unsaturated five-membered butyrolactone ring at the C-17 position along with a C-23 steroid structure, and bufadienolides, which have a six-membered double-unsaturated pyrone ring [26,27]. Together, cardenolides and bufadienolides constitute the class known as cardiac glycosides (CGs)—naturally produced chemical substances derived from secondary metabolites of plants and animals that consist of a sugar (glycone) and a non-sugar (aglycone) steroid component [28]. They are utilized to treat heart failure and also have good anticancer action via inhibition of Na^+^/K^+^-ATPase, initiation of cell apoptosis and autophagy, cell cycle regulation, and influence on cell signal transduction [29]. The therapeutic efficacy is attributed to the aglycone component, while the glycone is crucial for solubility and cell permeability [20]. In recent years, interest in cardiac glycosides (CGs) for cancer treatment has surged [30], as numerous CGs, including Bufalin **1**, Ouabain **2**, and Digoxin **3**, have been shown to decrease tumor cell development by inducing apoptosis [31,32,33,34]. Digitoxin **4**, another naturally occurring cardiac glycoside, has demonstrated significant antitumor activity in breast cancer [35,36] and brain tumors [37,38]. Digitoxin 4 exhibits a substantial cytotoxic effect on Gefitinib-resistant NSCLC H1975 cells by inducing cell cycle arrest, significantly activating AMPK, and decreasing α-tubulin. The CC50 value was found to be 0.19 ± 0.06 µM, which showed the strong inhibitory selectivity of digitoxin for NSCLC cells [39]. The apoptotic activity of five cardiac glycosides was analyzed in various human colon cancer cell lines, among which Oleandrin **5** exhibited the maximum inhibitory potential via the mitochondrial pathway. The results indicated that oleandrin significantly inhibits the proliferation of SW480 cells (IC_50_ = 0.02 µM) without affecting the viability of normal human colonic epithelial cells (IC_50_ = 0.56 µM). Moreover, it was found that it causes an increased concentration of intracellular Ca^2+^ ions, but it reduces the GSH concentration in cells [40]. Oleandrin also induces anti-proliferative activity in breast cancer cells by activation of the mitochondrial apoptosis signaling pathway and the initiation of endoplasmic reticulum stress. Additionally, it was discovered that oleandrin induces the destruction of breast cancer cells by promoting immunogenic cell death (ICD) through the PERK/eIF2α/ATF4/CHOP pathway [41,42]. In 2016, it was reported that, in contrast to digitoxin and Ouabain, Peruvoside **6** caused a greater degree of apoptosis in K562 and KG1a leukemic cells, via activation of the Caspase pathway. It demonstrated a comparatively low IC_50_ value in both cell lines and did not induce cytotoxicity in normal cells [43]. In the same year, peruvoside had shown time- and dose-dependent anti-proliferative activity against NSCLC such as H460 and Calu-3 lung cancer cells. Kaushik et al. (2017) [28] also explored the potential of another naturally occurring cardiac glycoside, i.e., Convallatoxin **7**, towards the same lung cancer cell lines. Convallatoxin exhibited twice the activity of peruvoside in both viability and colony formation assays, highlighting the importance of the aglycone region in determining therapeutic potency. Both drugs substantially decreased the cancer cells’ ability to migrate and invade and were able to alter proteins associated with autophagy, cell cycle regulation, proliferation, apoptosis, and EMT.

Glucoevatromonoside **8** was identified as a potent anti- lung cancer agent showing lower toxicity towards lung MRC-5 and PBMC, having greater binding affinity to the Na^+^/K^+^-ATPase α subunit, evaluated by molecular docking analysis. It induced an apoptotic effect by activating the caspase pathway, causing G2/M arrest, and downregulating cyclin B1 and p53 [44]. Further studies stated that Glucoevatromonoside 8 GEV decreased the survival of H460 cells over the short and long term without impacting the viability of healthy human peripheral blood mononuclear cells. It reduced the activity of Na^+^/K^+^-ATPase at high concentrations (µM) in the membranes of pig kidney cells and at lower concentrations in A549 and H460 cells, while not affecting the membranes of erythrocytes. Synergistic antiproliferative effects were shown when GEV was administered in combination with Irinotecan, Etoposide, Paclitaxel, and Cisplatin chemotherapeutic drugs. This was especially noticeable when GEV + CIS and GEV + PAC were combined [45]. The phytochemical analysis of six unidentified cardiac glycosides **9**, **10**, **11**, **12**, **13**, and **14** was carried out; these were isolated from the roots of Streblus asper Lour. Their anti-cancer potential has been examined in human A549 lung cancer cells, and it was found that each molecule exhibited exceptional inhibitory actions, with IC_50_ values falling between 0.01 and 6.08 μM. Moreover, compound 11 significantly reduced the growth and proliferation of A549 cells by inducing apoptosis through the activation of caspases-3, -8, and -9, as shown by Western blot analysis [46] (Figure 2).

The antiproliferative and anti-migratory effects of AnvirzelTM, a combination of two toxic cardiac glycosides, Oleandrin **5** and Oleandrigenin **15**, were studied in U87 cells. It significantly reduced the proliferation of U87 cells (*p* < 0.05) in a dose-dependent manner after 24 and 48 h of therapy. Furthermore, it was shown that in U87 cells, AnvirzelTM activated ERK while inhibiting the expression of NOS, GSK-3, and HIF1-α (*p* < 0.05) [47]. Ren et al. (2020) [48] isolated a cardiac glycoside named (+)-Strebloside **16** from the Streblus asper plant and identified its cytotoxicity using molecular docking analysis. According to the analysis, it was found that the compound docked deeply in the Na^+^/K^+^-ATPase pocket, and its C-14 and C-5, C-10 formyl, and C-4′ hydroxy groups showed hydrogen bonding interactions with Thr797, Glu117, Glu111, and Arg880 residues of Na^+^/K^+^-ATPase, respectively. Additionally, it did not affect glucose uptake in human lung cancer cells, despite demonstrating strong activity against them. This suggests that the cytotoxicity of this cardiac glycoside is mediated through targeting Na^+^/K^+^-ATPase rather than interacting with glucose transporters. Liu et al. (2021) [49] stated the anti-cancer potential of Deslanoside **17** in prostate cancer cells by controlling multiple pathways. It effectively inhibited colony formation and tumor growth in various prostate cancer cell lines by arresting the cell cycle at G2/M and inducing apoptosis. This anti-cancer compound changed the expression of several genes linked to different cancer-related signaling routes and cellular processes. α-L-diginoside **18** is a cardiac glycoside isolated from an indigenous plant by a research group in Taiwan. With an IC_50_ < 0.2 µM, α-L-diginoside exhibited potent anticancer activity in oral squamous cell carcinoma (OSCC) cell lines by inducing apoptosis and inhibiting various signaling pathways [50]. The anticancer activities of Proscillaridin A 19 were identified against prostate cancer cells. It 19 significantly reduced the growth, invasion, and migration of prostate cancer by inducing endoplasmic reticulum stress activation [51]. In another study, the researchers found that the most common secondary metabolite in the aqueous bulb extract of Drimia maritima is Proscillaridin A **19**. The extract was selectively cytotoxic to Caco-2 (IC_50_ 0.9 µg/mL) and COLO-205 (IC_50_ 2.3 µg/mL) colorectal cancer cells, but had less toxicity to normal fibroblasts (IC_50_ 13.1 µg/mL). The extract caused apoptosis, enhanced the production of ROS in COLO-205 cells, reduced the mitochondrial membrane potential, and raised the expression of CASP8, TNF-α, and IL-6 genes, extending the established anticancer profile of Proscillaridin A to colorectal cancer [52]. Ascleposide **20**, a cardiac glycoside, targets castration-resistant prostate cancer (CRPC) by inhibiting the pumping activity of Na^+^/K^+^ ATPase. This is achieved through p38 MAPK-mediated endocytosis of the enzyme, downregulation of the α1-subunit, tubulin acetylation, and cell cycle arrest [53]. Helveticoside **21**, isolated from the seed extract of Descurainia Sophia, confirmed its anti-cancer activity in colorectal cancer cells SW480 and HCT116 through in vitro studies. The cytotoxic activity of helveticoside involved the activation of the tumor suppressor p53 and the induction of mitochondria-mediated intrinsic apoptosis [54]. The anti-cancer effect of a naturally occurring cardiac glycoside named Strophanthidin **22** was examined in lung, breast and liver cancer cells via in vitro and in silico testing. The cytotoxicity of strophanthidin involved the arrest of the cell cycle at the G2/M phase through both p53-dependent and p53-independent mechanisms, and induced apoptosis by the reduction in several biochemical signaling pathways [55]. Ma et al. (2018) [56] investigated the in vitro anti-cancer potential of a cardiac glycoside, isolated from Helleborus thibetanus Franch, which was named HTF-1 **23**. It caused apoptosis against a variety of cancer cell types in a concentration- and time-dependent manner. Moreover, it was found to cause cytotoxicity by altering caspase-9, 3 PI3K-Akt-mTOR, and MAPK pathways. Human breast and lung cancer cell lines were used to test the anti-proliferative effects of two natural cardenolides, Calotropin **24** and Calotoxin **25**. The findings demonstrated the considerable cytotoxicity of calotoxin and calotropin against the MDA-MB-231 and A549 cell lines, with values ranging from 0.046 to 0.072 μM in comparison to the reference (0.47 to 58.41 μM) [57]. Recent research by Jayaraman et al. has broadened the anticancer profile of calotropin **24** to encompass oral squamous carcinoma (HSC-3 cells). The research indicated that calotropin suppresses cell growth by inducing G0/G1 phase arrest and apoptosis. It was the first to demonstrate that calotropin inhibits aerobic glycolysis in cancer cells, evidenced by reduced glucose uptake, lactate generation, and LDHA activity. It demonstrated anti-metastatic properties by obstructing cell migration and invasion. Molecular docking further validated its significant binding affinity to metastasis-related regions, confirming its great potential for oral cancer treatment [58].

Malayoside **26** exhibited inhibitory effects against NSCLC cells by inducing apoptotic pathways. Its anti-proliferative effect mainly included the activation of the MAPK-Nur77 signaling pathway [59]. Lanatoside C **27** exhibited anti-cancer effects against various cancer cell lines, notably in gastric cell lines SGC-7901 and MKN-45. It inhibited the growth, migration, and cell cycle arrest and death of MKN-45 human gastric cancer cells, which were linked to ROS generation and mitochondrial dysfunction. In addition, its anti-cancer actions were mediated by inhibition of the Wnt/β-catenin/c-Myc signaling pathway [60]. Rimpelová et al. (2021) [61] worked on the isolation, structure elucidation, and cytotoxicity of two steroid glycosides: Hyrcanoside **28** and Deglucohyrcanoside **29**. These glycosides were isolated from the Coronilla varia plant, and their anti-cancer potential was determined using in vitro and in silico studies in comparison to three other CGs: Ouabain, Digitoxin, and Cymarin. All of the CGs had the strongest anticancer potential (measured by cancer cell selectivity) when tested against lung and colorectal carcinoma cell lines. Additionally, every CG arrested the CRF-CEM cell cycle at the G2/M phase. From the methanolic extract of Solanum surattense, two steroid glycosides, Solamargine **30** and Dioscine **31**, were separated and their anticancer potential assessed. With IC_50_ values of 7.55 ± 1.5 and 3.3 ± 1.9 µg/mL, respectively, both compounds demonstrated remarkable anticancer activity against the NIH-3T3 fibroblast cancer cell line. These values were comparable to the control, clohexamide (IC_50_ value = 0.8 ± 0.2 µg/mL) [62]. Lee et al. (2018) [63] investigated the potential anti-cancer properties of Ophiopogonin D (OP-D) **32** and its mechanisms of action in non-small cell lung carcinoma (NSCLC) cells. It showed anti-proliferative properties by significantly suppressing STAT3 signaling, and this action was mediated by oxidative stress driven by a disruption in the GSH/GSSG ratio. Furthermore, OP-D reduced the expression of several carcinogenic genes, activated the caspase-mediated apoptotic cascade, and induced apoptosis. Malyarenko et al. (2021) [64] studied the anticancer effects of polyhydroxysteroid glycosides derived from the starfish Protoreaster lincki on colorectal cancer cells. The glycosides PL1 **33**, PL2 **34**, and L1 **35** suppressed 3D HCT 116 spheroids’ viability, colony expansion, and invasion, with Linckoside L1 **35** showing the highest activity. Cardiac glycosides (CGs) have anticancer effects by modifying cellular metabolism and mitochondrial function in addition to their traditional suppression of Na^+^/K^+^ ATPase. Proteome integral solubility alteration (PISA) tests were used in a recent study by Qin et al. (2024) [65] to examine the complex processes of Ouabain **2**, Digoxin **3**, and Digitoxin **4** in cancer cells. Several metabolic enzymes, such as PKM2 (pyruvate kinase M2), ANXA2 (annexin A2), SLC16A1 (monocarboxylate transporter 1), GOT2 (glutamate-oxaloacetate transaminase 2), and GLUD1 (glutamate dehydrogenase 1), were found to interact with CGs. Stable connections between these CGs and the indicated proteins were verified by molecular docking. Using column chromatography, beta-sitosterol glycoside **36** was separated from the ethyl acetate extract of Paraboea leuserensis, a plant native to Sumatra’s Leuser Mountain. The compound’s potential as an anticancer drug was demonstrated by its IC_50_ value of 24.83 mg/L (24.83 μg/mL) when tested for anticancer activity against MCF-7 human breast cancer cells using the MTT technique [66] (Figure 3). The structures, cancer cell types, and mechanisms of action of these steroid glycosides are summarized in Table 1.

### 2.2. Flavonoid and Polyphenol Glycosides

Seven known phenolic glycosides **37**, **38**, **39**, **40**, **41**, **42**, and **43** were isolated from the aerial portions of the Stipagrostis plumose plant, and their anti-cancer effects were checked through in vitro studies. Compared with the reference drug tamoxifen, all of them showed encouraging efficacy against the JEG-3 cell line. Fluorescence imaging research indicated the cytotoxicity mechanism, which involves the breakdown of the actin cytoskeleton and rupture of the cell nuclear membrane [67]. (-)-Epigallocatechin 3-O-gallate **44** and **45**, extracted from the leaves of Camellia sinensis var. assamica displayed considerable cytotoxic activities against CAL27 (9.78 ± 0.25 µM) and MDAMB231 (3.27 ± 0.18 µM) cell lines, compared to the reference, capecitabine with IC_50_ values of 8.20 ± 0.75 and 5.20 ± 0.89 µM, respectively [68]. Kim et al. (2020) [69] identified the cytotoxic potential of α-mangostin glycosides, designated Man-3DG **46** and Man-6DG **47,** on hepatocellular carcinoma (HCC) cells. By inhibiting c-Met signaling and HIF-1α expression, the analogs efficiently reduced the proliferation, migration, and tumor angiogenesis of HCC cells. A flavanonol glycoside, Engeletin (dihydrokaempferol 3-rhamnoside) **48,** exhibited anticancer properties in certain tumor types. It was observed that lung cancer cells underwent ENG-induced apoptosis, a mechanism controlled by ER stress. This process enhanced SMAC expression, which contributed to the inhibition of XIAP. Furthermore, according to the in vitro research, lung cancer cells treated with ENG showed mitochondrial dysfunction [70]. Elatif et al. (2020) [71] identified the anti-cancer properties of the chemical extract of Salicornia fruticose plant. The extract contained some active glycosides such as isorhamnetin 3-O-glucoside **49**, isorhamnetin 3-O-rhamnosyl (1–2) arabinoside **50**, isorhamnetin 3-O-rhamnoside **51**, isorhamnetin 3-O-rutinoside **52**, and isorhamnetin 3-O-neohesperidoside **53**. Their cytotoxic effects were checked against HepG2, MCF-7, A549 and HCT-116 cancer cell lines using the MTT assay, and the results showed their excellent potency with IC_50_ values of 2.6. 10.9, 37.9, and 5.4 (mg/mL), respectively, compared with reference doxorubicin (Figure 4).

Quercetin-3-O-β-D-galactopyranoside **54**, also known as Hyperoside, is a flavonol glycoside that possesses anti-cancer properties. In vitro studies have shown that it could prevent A549 cells from proliferating by causing apoptosis and G1/S phase arrest [72]. Moreover, hyperoside reduced proliferation and caused apoptosis in T790M-positive NSCLC by upregulating FoxO1 via CCAT1, indicating that it is a novel potential for treating T790M-positive NSCLC [73]. In a study conducted by Ervina et al. (2020) [74], the cytotoxicity of *Melia azedarach* L. wild-type leaves extract was analyzed. The secondary metabolites were extracted from the ethanolic extract of Melia leaves by fractionating it with n-hexane, ethyl acetate, and water. The findings demonstrated that the ethyl acetate fraction had the highest cytotoxic potential against T47D cells, with an IC_50_ value of 147.90 ± 8.49 mg/mL, compared with the others. It was revealed that the fraction contained quercetin glycosides **55** and **56** along with other phytochemicals. Mohammed et al. (2021) [75] aimed to evaluate the anti-cancer activities of phytoconstituents of Z. coccineum, a halophytic edible plant. It is proposed that the presence of flavonoid glycosides (Tiliroside **57**, Isorhamnetin-3-O-glucosid **58**, Isorhamnetin-3-O-rutinoside **59**, Rutin **60**, Spiraeoside **61**) had anti-cancer properties. The aqueous-ethanolic extract inhibited h-TOP-IIβ with an IC_50_ value of 45.05 ng/mL, compared to the standard reference inhibitor staurosporine (IC_50_, 135.33 ng/mL). Additionally, it exhibited cytotoxic effects with IC_50_ values of 3.19, 3.47, and 2.27 µg/mL against HCT-116, MCF-7, and HepG2 cell lines, respectively. Tiliroside’s anticancer potential has been further confirmed by molecular docking studies, which show that it has a strong binding affinity to epidermal growth factor receptor (EGFR) kinase with a score of −10.4 kcal/mol, better than the reference drug erlotinib (−9 kcal/mol). MMGBSA calculations and molecular dynamics simulations verified stable binding with a free energy of −42.17 kcal/mol, which is similar to erlotinib. The author also reported Isoquercetin (quercetin-3-O-β-D-glucopyranoside) **62**, a flavonol glycoside, as a potent cytotoxic metabolite through computational screening. Molecular docking studies revealed that isoquercetin exhibits strong binding affinity to epidermal growth factor receptor (EGFR) kinase with a score of −10.1 Kcal/mol, surpassing the reference drug erlotinib (−9 kcal/mol). This computational evidence, combined with its favorable pharmacokinetic properties predicted by SWISS ADME modeling, suggests that isoquercetin warrants further experimental investigation as a potential EGFR-targeting anticancer agent [76]. LC-MS analysis of the extract of Premna barbata also revealed the flavonol glycoside rutin **60**. The extract was found to have a high level of cytotoxicity towards COLO-205 colon cancer cells, implying a potential involvement of rutin in the anticancer efficacy [77]. Synthesis and characterization of a novel oxidovanadium cation containing the flavone glycoside Diosmin **63** have been completed by Naso et al. (2016) [78]. It is observed that the anti-cancer potential of diosmin in various human cancer cell lines has been enhanced by the generation of its metal complex. ROS generation and enhancement in cytoplasmic condensation accompanied the anti-cancer effects on the human lung cancer cell line. Solamargine **64** and Solasonine **65**, the important phytochemical constituents of the family Solanaceae, exhibited anticancer properties by influencing many biological pathways in a diverse array of human cancer cells [79,80,81,82,83,84,85,86,87,88,89,90,91,92] (Figure 5).

A subclass of flavonoid glycosides called anthocyanins, found in many fruits and vegetables, has been shown to have potential anti-cancer effects. In a rat model of colorectal cancer generated by DMH, the anthocyanin glycoside cyanidin-3-glucoside (C3G) **66** showed notable chemoprotective properties. When C3G (10 and 15 mg/kg) was administered, body weight was restored, colon and spleen weights were decreased, and tumor incidence and burden were controlled. The substance suppressed NF-κB signaling, decreased tumor markers (CFA, CA19.9, LDH, NO), and downregulated inflammatory mediators (TNF-α, IL-1β, IL-1α, IL-2, IL-4, IL-6, IL-10, IL-12, IL-17, COX-2, PGE2, iNOS). Additionally, C3G stimulated apoptosis by regulating the Bax/Bcl-2 apoptotic axis and activating caspases-3, -6, and -9. These results confirm C3G’s potential as a therapeutic agent for the treatment of colorectal cancer by indicating that it modulates NF-κB and the Bcl-2/Bax/Caspase pathway to produce its anticancer effects [93]. Naringenin-7-O-glucoside **67**, a flavanone glycoside isolated from Holarrhena antidysenterica, was investigated for its anticancer potential against triple-negative breast cancer (TNBC) cell lines. With an IC_50_ value of 233.56 µg/µL, the chemical demonstrated dose-dependent cytotoxicity against MDA-MB-231 and MCF-7 cells and negligible toxicity toward normal Vero cells, suggesting selective anticancer action. Mechanistic studies revealed that naringenin-7-O-glucoside induced apoptosis, as evidenced by extensive DNA fragmentation [94]. Hesperidin **68**, a flavanone glycoside, demonstrated dose-dependent cytotoxicity against gastric cancer AGS cells by triggering apoptosis via elevated BAX and reduced BCL-2 expression. Mechanistic studies revealed downregulation of PI3K signaling, a key pathway in cancer cell survival. These findings support hesperidin’s potential as a therapeutic agent for gastric cancer [95]. As a subclass of flavonol glycosides, kaempferol glycosides have become powerful inhibitors of the PI3K/Akt pathway, which is often imbalanced in cancer. In a recent work, Jamshidi et al. (2025) [96] used molecular docking to assess the Akt1 inhibitory efficacy of 61 compounds generated from flavonoids. In comparison to the control inhibitor Ipatasertib (ΔG = −9.98 kcal/mol), two kaempferol glycosides, kaempferol 3-rutinoside-4′-glucoside **69** and kaempferol 3-rutinoside-7-sophoroside **70**, showed remarkable binding affinities to the Akt1 ATP-binding site (ΔG = −21.79 and −20.73 kcal/mol; Ki = 106.03 aM and 640.24 aM). After 30 ns, molecular dynamics simulations verified that kaempferol 3-rutinoside-4′-glucoside had a stable binding conformation inside the Akt1 catalytic domain. Haplanthodes tentaculatus, a native plant in the Western Ghats of India, was found to be a source of a new flavanone glycoside, 5-hydroxy-7-methoxy-8-O-2S-flavanone-glucopyranosyl-2S-flavanone **71**. Characterization of the compound was done by preparative HPLC, NMR and HRMS. It was observed to have anticancer activity when analyzed against MDA-MB-231 breast cancer cells by the MTT assay, and cytotoxic effects were found to be high, with an IC50 of 155.21 ± 1.59 µM [97]. The Polyphenolic contents of Carlina acaulis were analyzed through UHPLC-HR/QTOF/MS-PDA, where several chlorogenic acids and various flavonoid C-glycosides were found. The glycosides were identified as schaftoside **72**, isoorientin **73**, vitexin-2-O-rhamnoside **74**, vicenin II **75** and kaempferol dihexosidipentose **76**, with schaftoside (~3 mg/g) being the most common flavonoid. The most active anticancer effect was demonstrated in the ethyl acetate fraction, which was the richest in polyphenolics, on HT29 colorectal and HeLa cervical cancer cells [98] (Figure 6).

Phytocompounds from Moroccan plants were subjected to molecular docking and virtual screening to assess their potential as anticancer drugs that target the EGFR and VEGFR2 receptors. Two glycosides from *Ajuga iva* L. were shown to be noteworthy active compounds: 3-glucoside resveratrol **77** and 4-O-glucoside ferulic acid **78**. Promising binding affinities to the target receptors were shown by both compounds. Molecular dynamics simulations and predictive ADMET analysis provided additional evidence for their stability and drug-like characteristics [99]. The anticancer potential of Colvillea racemosa flower and leaf extracts was assessed using A549 non-small cell lung cancer cells. Both extracts showed significant cytotoxicity with IC50 values of 17.0 µg/mL (leaf) and 17.2 µg/mL (flower). Forty-two metabolites were identified by UPLC-ESI-QTOF-MS analysis, with flavonoid C-glycosides being the most prevalent. Four flavonoids were chosen for molecular docking against lung cancer targets (γ-PI3K, EGFR, CDK2): vitexin **79**, kaempferol-3-O-rutinoside **80**, isoorientin **81**, and luteolin. Vitexin and kaempferol-3-O-rutinoside exhibited the greatest binding scores on CDK2 and γ-PI3K, indicating that they may play a major role in the extract’s anticancer action [100]. Ononin **82**, an isoflavone glycoside derived from traditional Chinese medicinal plants, induced ferroptosis in colorectal cancer cells by increasing lipid ROS via the PI3K/AKT/Nrf2 pathway and reducing GPX4 expression. Ononin’s potential as a therapeutic agent and immuno-adjuvant for colorectal cancer was demonstrated in an MC38 mouse model, where its combination with anti-PD-L1 therapy raised intratumoral IFNγ + CD^8+^ T cells and improved tumor growth suppression [101]. The flavonoid C-glycosides vicenin-II **83** and isovitexin **84**, as well as kaempferol-3-O-neohesperidoside **85**, were isolated for the first time from Dypsis pembana leaves. Spectroscopic data were used to characterize each molecule. HepG-2 liver cancer cells were used to examine the extracted compounds’ cytotoxic potential. With an IC_50_ of 14.38 µg/mL, Vicenin-II exhibited the strongest activity, followed by isovitexin with an IC_50_ of 15.39 µg/mL. Vicenin-II’s potential as a lead structure for the development of anticancer drugs was supported by molecular docking experiments that showed superior binding affinities to human topoisomerase IIα and cyclin-dependent kinase 2 [102]. The phytochemical profile of Leontodon hispidulus ethanolic extract was analyzed by LC-qToF-MS and GC-MS, revealing 36 phenolic compounds including glycosides of luteolin, quercetin, kaempferol, apigenin, isorhamnetin, and daidzein, along with the coumarin glycoside esculin. With IC_50_ values of 19 and 19.6 μg/mL, respectively, the methylene chloride and ethyl acetate fractions exhibited the most cytotoxic effect against PC3 prostate cancer cells. Apigenin-7-O-glucoside **86**, luteolin-7-O-glucoside **87**, kaempferol-3-O-glucuronide **88**, quercetin-4′-O-glucoside **89**, and esculin **90** all showed strong binding affinities to the target, according to molecular docking against hexokinase-2 [103]. Kaempferol 3-O-[(6-O-caffeoyl)-glucosyl(1→2)]-(6-O-caffeoyl) glucoside-7-O-rhamnoside **91**, a novel flavonol glycoside, was extracted from Clematis flammula leaves and identified using NMR and HR-MS. Promising findings were obtained from cytotoxicity assays on HepG2 hepatoma, A2780, and OVCAR3 ovarian cancer cells, especially against A2780 cells with an IC_50_ of 77.0 μg/mL, which is equivalent to taxol (76.9 μg/mL) [104] (Figure 7). A summary of these flavonoid and polyphenol glycosides, including their key mechanisms and anticancer potency, is provided in Table 2.

### 2.3. Terpene Glycosides

Two sesquiterpene glycosides, **92** and **93,** that were isolated from S. mukorossi were found to have anti-cancer properties against breast cancer. According to the XTT assay results, the 50% inhibitory concentrations ranged from 120.73 to 154.01 µg/mL, indicating weaker inhibition compared to their parent fraction. At a concentration of 200 µg/mL, their inhibitory rates against three cancer cell lines (MCF-7, MDA-MB-231, and MDA-MB435s) approached approximately 80%. Additionally, the mechanisms underlying breast cancer were anticipated, and 22 targets, including IL2, PTPN1, and VEGFA, were found to be quite significant [105].

Dioscin **94**, a steroidal saponin found in the Polygonatum plant, displayed cytotoxic activities towards various cancer cell lines. Dioscin appears to trigger anti-tumor immunity in lung cancer patients by preventing macrophage polarization [106]. Dioscin may cause liver cancer cells to undergo apoptosis via cell cycle arrest [107]. Hernández-Vázquez et al. (2020) [108] reported the anti-proliferative activity of dioscin on cervical HeLa and CaSki cancer cells via in vitro studies. Yen et al. (2021) [109] isolated anti-cancer Oleanane-type triterpene saponins **95a**, **95b**, **96**, and **97** from *Aralia armata*. Three human cancer cell lines (A2058, HT29, and A549) were shown to be susceptible to the cytotoxic effects of compounds **67–97**, with IC_50_ values between 2.01 ± 0.17 and 18.8 ± 1.17 μM. Compound **95b** (narcissiflorin) exhibited notable cytotoxic activity against HT29 and A549 cell lines, with IC_50_ values of 2.02 ± 1.65 and 2.01 ± 0.17 μM, respectively. These values are lower than those of irinotecan hydrochloride, the positive control, which had IC_50_ values of 10.3 ± 1.32 and 9.89 ± 0.19 μM. Glycyrrhizin triterpene **98**, which is a naturally occurring glycoside, suppresses cell migration and invasion and induces apoptosis and autophagy in colorectal cancer cells by inhibiting the expression of MMP-9 and MMP-2 [110]. This compound has shown anti-cancer effects against various other cancer cells such as melanoma cells [111], ovarian cancer A2780 Cells [112], colon cancer cells [113], and cervical cancer cells [114]. Ha et al. (2021) [115] analyzed the cytotoxic potential of steroidal oligoglycosides **99** and **100**, which were isolated from the starfish Acanthaster planci. Findings have shown that both compounds displayed slight anti-cancer activities against HT-29, RPMI-7951, and MDA-MB-231 cell lines, but successfully prevented MDA-MB-231 cell migration and HT-29 and MDA-MB-231 cell colony formation at non-toxic concentrations. The necrotic, apoptotic, and antiproliferative properties of DSG and its glycoside derivatives **101**, **102**, and **103** were studied against cervical cancer cells through in vitro analysis. The findings indicated that DSG glycosides were more effective at inducing apoptosis than DSG, indicating that rhamnose and glucose residues are crucial in raising the apoptotic activity of DSG. Furthermore, because of their selective action and lower necrotic activity, DSG and its glycosidic derivatives are prospective anticancer agents [108]. Two promising anti-cancer compounds **104** and **105** were isolated from Tinospora cordifolia. Compound **104**, a clerodane furano diterpenoid, demonstrated anticancer efficacy in HCT116 cells by inducing autophagy and mitochondria-mediated cell death. Findings from in silico studies of compound **105**, a norditerpene furan glycoside, displayed good binding affinity for the oncogene and membrane tyrosine kinase HER2 protein [116,117]. The anti-proliferative properties of β-Escin **106**, a triterpene saponin, extracted from horse chestnut seed, have been described. The anti-cancer mechanism includes the upregulation of p21, a reduction in the production of cyclin D1, and the inhibition of apoptosis-inducing Bcl-2 protein family members [118] (Figure 8). The anticancer activity of five cycloartane-type saponins, including Astragaloside IV **107**, Cyclocanthoside E **108**, Astrasieversianin X **109,** Macrophylosaponin B **110** and Macrophylosaponin D **111** against MCF-7 and MDA-MB-231 breast cancer cells was assessed. The cytotoxicity was determined at 10, 100, 150 and 200 μM concentrations during 24, 48 h and 72 h using Cock-8 assay. MDA-MB-231 cells of MDA-MB-231 showed a high level of cytotoxicity at all concentrations during all time intervals. Apoptosis analysis using the TUNEL assay showed significant apoptosis induction in MCF-7 cells at 10 and 100 μM after 48 h. The study demonstrates that low-dose saponin glycosides reduce breast cancer cell viability and induce apoptosis [119]. Six main saponins, including saponariosides C **112**, D **113**, and E **114**, as well as derivatives of gypsogenin and gypsogenic acid, were identified from root extracts of Saponaria officinalis from Cyprus using UHPLC/Q-TOF-MS. Additionally, six phenolic compounds were found, including quercetin galactoside **115** and rutin **60**. In addition to having antibacterial and antioxidant properties, the acetone extract significantly inhibited the growth of A375 melanoma cells in a concentration-dependent manner [120]. Phytochemical analysis of Ziziphus spina-christi leaf extract produced two new dammarane-type saponins, christinin E **116** and christinin F **117**, along with the known christinin A **118**. The isolated compounds were evaluated for cytotoxic activity against cell lines of A549 lung cancer, U87 glioblastoma, MDA-MB-231 breast cancer, and CT-26 colorectal carcinoma at 1–20 µg/mL concentrations. All three compounds exhibited potent activity at low concentrations (<10 µg/mL) with low IC_50_ values. To find further possible targets and interactions with cancer-related signaling pathways, network pharmacology and in silico analyses (inverse docking, molecular dynamics simulations) were carried out [121] (Figure 9).

### 2.4. Terpenoid Glycosides

Kutkoside **119**, an iridoid glycoside, inhibited cell migration and invasion and had anti-proliferative effects on drug-resistant human oral cancer cells by targeting the PI3K/AKT signaling pathway. Using flow cytometry, the percentage of apoptotic cells was found to be 4.9%, 12.18%, 22.18%, and 34.10% at various dose exposures of Kutkoside, which were 0, 8, 16, and 32 μM [122]. Kuhtinskaja et al. (2020) [123] demonstrated the anti-cancer effect of bis-iridoid glycosides, namely Sylvestroside III **120** and Sylvestroside IV **121**, against different human cancer cell lines. The results have shown that the iridoids had a specific anti-proliferative effect on several cancer cells, but they had no effect on healthy cells. Six gypenosides were obtained from a phytochemical study of Gynostemma pentaphyllum. The compounds included two with a 21,23-epoxy group **122**, **123** and two with a 21,23-lactone skeleton **124**, **125**, exhibited moderate anticancer activity against HepG2 by inhibiting HIF-1a mRNA expression and disrupting HepG2 migration and invasion, in comparison to reference compound [124]. Two iridoid glycosides, aucubin **126** and ajugol **127**, were isolated from Verbascum speciosum using phytochemical analysis. IR, NMR, and LC-TOF/MS spectroscopic techniques were used to determine the structures of these compounds. Aucubin demonstrated selectivity for cancer cells over normal 3T3-L1 fibroblasts and dose-dependent antiproliferative efficacy against MDA-MB-453 breast cancer and A-549 lung cancer cell lines. According to mechanistic investigations, aucubin inhibits the PI3K/AKT signaling pathway to cause early apoptosis in MDA-MB-453 cells. Ajugol, while not evaluated for anticancer activity in this study, adds to the library of iridoid glycosides with potential for future investigation [125]. The ethyl acetate extract of Premna barbata stem bark exhibited selective cytotoxicity on the COLO-205 colon cancer cells (GI50 44.6 0.5 g/mL). The analysis of the sample by LC-MS showed the presence of the iridoid glycoside geniposidic acid **128**, which could be the source of the anticancer activity observed [77] (Figure 10). These terpenes and terpenoid glycosides, along with their most potent compounds and IC50 values, are summarized in Table 3.

### 2.5. Other Glycosides

Sikdar et al. (2015) [126] evaluated the anti-cancer potential of glycoside-rich components of condurango vine on lung cancer cells via in vitro studies. The H460 cell viability results demonstrated that a significant proportion of cancer cells may be destroyed in 24 h by 32 µg/mL (the IC_50_ value) of Condurangogenin A **129** (an esteric glycoside). Furthermore, its inhibition mechanism involves DNA damage-induced apoptosis and p21/p53 mediated cell cycle modulation. From the seeds of Caesalpinia sappan, three novel ester glycosides were isolated and given the names Caesateroside A **130**, Caesateroside B **131**, and Caesateroside C **132**. Weak to moderate cytotoxicity was demonstrated by these compounds towards HepG-2 and HeLa human cancer cell lines [127]. The naturally occurring cyanogenic glycoside Amygdalin **133** is found in the seeds of various edible plants, including bitter almonds and peaches. Numerous studies using in vitro cell culture demonstrated amygdalin actions that could be helpful in the treatment of cancer. In human prostate cancer cells, amygdalin treatment increased Bax expression, decreased Bcl-2 expression, and induced caspase-3 activation [128]; induced apoptosis of HeLa cervical cancer cells via the endogenous mitochondrial pathway [129]; and decreased adhesion and migration of UMUC-3 and RT112 bladder cancer cells by activating focal adhesion kinase and modulating β1 integrin [130]. Additionally, amygdalin can prevent the production of genes that prevent cell death, such as XIAP and survivin [131]. The antitumorigenic potential of amygdalin-containing apricot oil extract for oral cancer has been evaluated. The extract significantly decreased cancer cell viability through upregulation of autophagy, enhanced apoptosis, and cell cycle arrest. Cancer cell migration was also reduced. At low doses, the extract showed no significant toxicity to normal oral epithelial cells [132]. Another cyanogenic substance, Linamarin **134**, which is present in the leaves and roots of cassava plants, is a potential anti-cancer compound [133]. It has shown great cytotoxic effects towards HeLa, ovarian, MCF-7 and HT-29 cancer cell lines [134,135]. A potential mechanism of linamarin is its effect on the respiratory chain of mitochondria via hydrogen cyanide, leading to the death of cancer cells [136]. The anti-proliferative effect of CTPG (Cistanche tubulosa phenylethanoid glycosides) **135** against hepatocellular carcinoma cells was determined. In vitro analysis showed that CTPG promoted apoptosis in HCC cells via the mitochondria-dependent and MAPK signaling pathways. In addition, CTPG decreased the growth of HCC by directly preventing tumor growth and indirectly boosting immunity when combined with cisplatin [137].

Two phenylethanoid glycosides, verbascoside **136** and poliumoside **137**, were separated and identified using spectroscopic techniques from the Verbascum speciosum extract. Verbascoside showed preference for cancer cells over normal 3T3-L1 fibroblasts and dose-dependent cytotoxicity against A-549 lung cancer and MDA-MB-453 breast cancer cell lines. Although poliumoside’s anticancer potential was not assessed in this work, it is a structurally similar phenylethanoid glycoside that requires further research [125]. The cytotoxic activity of three glycosides from P. lanceolata inflorescences named acteoside **138**, plantamajoside **139** and isorhamnetin-3-O-rutinoside-4-O-glucoside **140** was analyzed by MTT against seven human carcinoma cell lines (MCF-7, MDA-MB-231, Caco-2, HepG2, OVCAR-3, U1. The phenylethanoid glycosides (acteoside and plantamajoside) were more cytotoxic than the flavonoid glycoside. Acteoside (200 µM) and plantamajoside (300 µM) showed toxicity against MDA-MB-231, MCF-7, OVCAR-3, U138-MG and HepG2 cells at lower concentrations of 400 µM [138]. Using silica gel chromatography, a novel tetrahydrobenzochromone glycoside derivative **141** was extracted from the leaves of Melochia corchorifolia. The anticancer activity of the compound against the MCF-7 cancer cell line demonstrated an IC_50_ value of 120.71 ± 3.46, indicating its therapeutic efficacy [139]. Two glycosides, **142** and **143,** that were isolated from Asparagus cochinchinensis showed moderate anti-proliferative activities against cervical cancer cells with IC_50_ values of 35.5 and 39.6 μM, respectively [140] (Figure 11). The anticancer potential of bergenin **144**, a C-glycoside of 4-O-methylgallic acid that was extracted from medicinal plants like Bergenia crassifolia, against cervical carcinoma cells was studied. Bergenin affects several cancer hallmarks, such as angiogenesis, apoptosis, and tumor suppressor proteins, according to a proteomic study. By lowering the important angiogenic proteins Galectin-3 and MMP-9 in cervical cancer cells, it showed anti-angiogenic actions. Bergenin has a greater binding affinity to MMP-9 than Galectin-3, according to molecular docking [141]. Polygonum multiflorum roots yielded seven stilbene glycosides, including a novel chemical called stiquinoside A **145**. The growth of H2452 malignant pleural mesothelioma (MPM) cancer cells was markedly suppressed by stiquinoside A **145**, compounds **146**, and **147** among the isolated compounds, indicating their potential as anticancer medicines for MPM treatment [142]. Ipomoea biflora was used to isolate eight novel pentasaccharide resin glycosides, ipomofins I–VIII, as well as five previously identified ones. These were then studied using spectroscopic and chemical techniques. Certain cancer cell lines were cytotoxically affected by compounds **148**, **149**, **150a**, **150b** and **151**. Notably, **150b** showed potential as an anticancer agent through ERS pathway activation by impairing colony formation, decreasing EdU-positive cells, and increasing expression of endoplasmic reticulum stress-related proteins in HCT-15 cells [143] (Figure 12 and Figure 13).

The remaining glycosides with anticancer activity that do not fall into the categories above are summarized in Table 4.

## 3. Marine Derived Glycosides

The great diversity of biological components found in the marine environment makes it an excellent place to find new therapeutic anti-cancer drugs [144,145]. Marine-derived bioactive compounds, including alkaloids, flavonoids, phenols, sterols, terpenoids, and various secondary metabolites, have demonstrated significant anticancer activities. These compounds exhibit diverse chemical compositions and target different signaling pathways to combat various types of cancer [146]. Marine anti-cancer glycosides have attracted a lot of interest because of their distinct structures and diverse biological functions. The structures of marine glycosides with anti-cancer properties, isolated from marine organisms, their name, cancer cell types and mechanisms of action are summarized in Table 5.

The chemical structures of these marine-derived anti-cancer compounds are illustrated in Figure 14, Figure 15, Figure 16, Figure 17, Figure 18, Figure 19 and Figure 20.

### 3.1. Comparative Analysis of Marine Glycosides

#### 3.1.1. Structural Diversity and Source Distribution

The marine glycosides under review belong to three main taxonomic groups: sea cucumbers (Holothuroidea), starfish (Asteroidea), and marine actinomycetes. The largest group of sea cucumber glycosides in Table 5 is triterpene glycosides, which contain a characteristic 18(20)-lactone ring on the holostane-type aglycone. Polyhydroxysteroid glycosides are steroids obtained from Starfish that have a 3β,6α-dihydroxylated steroid backbone and 1–4 monosaccharide groups. Angucycline glycosides are glycosides that have a benz[*a*]anthracene aglycone and deoxy sugar side chains.

#### 3.1.2. Potency Comparison Across Marine Sources

Based on the IC_50_ values found in Table 5, it is apparent that there are different patterns of potency. The angucycline glycosides derived from actinomycetes exhibit significantly higher potency than do the glycosides from sea cucumbers. However, because normal-cell cytotoxicity data are lacking for the actinomycete-derived compounds, it was not possible to compare their increased potency against their selectivity.

#### 3.1.3. Mechanistic Convergence and Divergence

There are similarities and differences in the mechanisms of action of marine glycosides. Polyhydroxysteroid glycosides found in the starfish share the Na^+^/K^+^-ATPase inhibition pathway that has been studied for cardiac glycosides in plants, and as such, they produce cytotoxic effects. Sea cucumber triterpene glycosides also decreased apoptosis induction (echinoside A 179 having IC_50_ = 2.4 µM) and produced ceramide (stichoposide D 182 and C 183). Unlike other classes of glycosides, the actinomycetes glycoside angucycline shows a unique anti-migration/invasion activity at sub-cytotoxic concentrations, which implies anti-metastatic activity without any direct cytotoxicity.

#### 3.1.4. Unique Advantage of Marine Glycosides

Marine glycosides have three distinguishing characteristics from their counterparts found in plants. The sulfation at different positions improves the water solubility of the glycoside and still allows it to penetrate the membranes and molecular targets. This suggests that these may better penetrate tumor tissue than the plant cardiac glycosides.

## 4. Synthetic Glycosides

### 4.1. O-Glycosides

Pregnenolonyl-α-glucoside (**228**) is a steryl glycoside that is produced by a sequential multienzyme-catalyzed process from the reaction of pregnenolone and glucose. Anticancer studies have revealed that it exhibits notable dose-dependent cytotoxic activity with IC50 values ranging from 23.5 to 50.9 µM against AGS, HT29, and ES-2 cells. According to an in vitro experiment and protein–ligand docking model, it showed binding interactions in the heme iron pocket of the CYP17A1 active site (Scheme 1) [191].

Many glycoconjugate derivatives of 8-HQ (**240a–d**, **241a–d**, and **243–250**) were produced, and their biological activity was examined in vitro. The inhibitory potency of the obtained glycoconjugates was evaluated against a variety of cancer cell lines (MCF-7, HCT 116, PANC-1, HeLa, U-251, Hs683, and AsPC-1). Among the tested compounds, the conjugates **240a–d** and **241a–d** showed promising anticancer activities. The study revealed that the utilization of the glycoconjugation approach improved the bioavailability as well as the activity of 8-HQ derivatives. The activity of these derivatives relies on the specific type of attached sugar, the presence of protecting groups within the glycone, and the existence of a linker between the quinolone aglycone and the sugar (Scheme 2) [192].

Diosgenin (**255**) was used as the basis for the design and synthesis of nine glycosylated derivatives. Using the MTT method, the anticancer properties of the targeted compounds, **257a–i**, and the intermediate compounds, **256a–i**, were examined against the human leukemia and melanoma cell lines. These compounds displayed strong inhibitory effects on the four cancer cell lines. Additionally, compared to other cell lines, these derivatives exhibited stronger inhibitory activities against K562. Furthermore, compounds **257a–i** demonstrated greater activity than their intermediates **256a–i** (Scheme 3) [193].

Fujitaka et al. (2019) [194] applied chemo-enzymatic methods to synthesize **262a**, **b** the ester-linked glucoside conjugate of docetaxel. In the initial synthesis step, phenyl *α*-D-glucopyranoside reacted with hydroxypropionic acid in the presence of α-glucosidase, yielding carboxyethyl *α*-D-glucopyranoside. Then, the 2′-TES ester of docetaxel reacted with carboxyethyl *α*-D-glucopyranoside to yield **262a**, and further desilylation produced **262b**. The compound has shown strong in vivo anticancer action, effectively suppressing tumor growth as compared to the parent compound (Scheme 4).

Eight novel dihydroartemisinin-*O*-glycosides were synthesized, and their relative configurations were established by Yu et al. An in vitro immunosuppressive experiment revealed that **268a** exhibited a 98% reduction in IFN-γ levels in cell medium and an 88% inhibition of T cell proliferation. These findings highlight dihydroartemisinin-*O*-glycoside as a potent candidate for additional in vivo studies (Scheme 5) [195].

The Koenigs-Knorr reaction has produced a novel class of phenolic glycoside esters, saccharumoside-B and its analogs (**277a–n**, **278**). The compounds (**277a–n**, **278**) were tested for their antiproliferative properties using a range of cancer cell lines by MTT assay. With an IC_50_ range of 4.43 ± 0.35 to 49.63 ± 3.59 µM, compounds (**277a–n**, **278**) showed significant antiproliferative effects against cancer cells; however, their cytotoxicity against normal cells is lower (IC_50_ > 100 µM). Among all the compounds, **277f** exhibited significant antiproliferative activity with IC_50_ values of 4.43 ± 0.35 and 6.13 ± 0.64 against MCF-7 cells and HL-60 cells, respectively (Scheme 6) [196].

Nguyen et al. (2018) [197] prepared three novel *O*-Glycoside derivatives of anthraquinones named anthraflavic acid-O-glucoside **282**, 2-amino-3-*O*-glucosyl anthraquinone **283**, and alizarin 2-*O*-*β*-D-glucoside **284**. In the anti-proliferative assay, the synthesized glucoside compounds demonstrated over 60% inhibition of cell growth at concentrations ranging from ~50 µM to 100 µM against three different cancer cells: AGS, HeLa, and HepG2. Additionally, alizarin 2-O-glucoside, one of the glucoside derivatives produced, inhibited over 90% of the proliferation of all the cancer cell lines (Scheme 7).

Sharipova et al. (2019) [198] synthesized and evaluated the cytotoxicity of a range of glycosides and glycoconjugates of the diterpenoid isosteviol against a variety of human cancer and normal cell lines. Most of the compounds produced showed moderate to considerable cytotoxicity towards M-Hela and MCF-7 cell lines. The most active compounds demonstrated selective cytotoxic activity with IC_50_ = 10.0–15.1 µM against M-Hela, which is three times more effective than the anticancer medication Tamoxifen (IC_50_ = 28.0 µM). It was shown that the cytotoxic activities of the compounds resulted from their stimulation of apoptosis, which proceeded along the mitochondrial pathway (Scheme 8).

Elganzory et al. (2022) [199] developed new 1,3,4-thiadiazole thioglycosides using a 1,3-dipolar cycloaddition reaction. By reacting azide derivatives with the appropriate acetylated glycosyl terminal acetylenes, new hybrid compounds, **308a**, **b** and **312a–d,** were synthesized using the click approach. Using the MTT assay, the cytotoxic properties of the compounds were investigated against the HCT-116 and MCF-7 cancer cell lines. The findings demonstrated that the primary thiadiazolethione compounds, the free hydroxyl glycoside, and the triazole glycosides connected to derivatives of *p*-methoxyarylidine had strong anti-MCF-7 activity, compared to that of the reference drug, doxorubicin. Additionally, in silico studies were conducted to determine the binding patterns of the synthesized compounds (Scheme 9).

Xie et al. (2023) [200] aimed to design and synthesize flavonoid glycosides from hesperetin and determined their anticancer activities against human breast cancer cells. Hesperidin was converted to the benzyl-protected flavone glycone through specific methylation, oxidation, and benzylation processes. Next, the glycosides were condensed with peracetylated brominated sugars in the presence of DMF/K_2_CO_3_, and then the flavonoid glycosides were produced through an acetylation and debenzylation reaction. The majority of the compounds exhibited significant antihuman TNBC activity. Compounds **318a–c**, **319a**, and **319c** demonstrated growth inhibition effects on human TNBC cell lines to varying degrees; their IC_50_ values were all less than 16.61 μM. Moreover, compound **318b** had the highest cytotoxicity, with an IC_50_ value of 1.38 ± 0.31 μM, whereas the other compounds showed no activity at the highest dose tested (20 μM), with inhibition rates less than 50% (Scheme 10).

Liu and Kong (2018) [201] developed the enzymatic synthesis of acylated steroidal glycosides, and their biological activities have been determined to be potent anticancer agents. By employing OsSGT1 and EcSGA1 as the biocatalysts, a unique two-step enzymatic synthesis of two ASGs was developed from the readily available free steroids. EcSGA1 catalyzed the late-stage regioselective acylations of all hydroxyl groups on the glycone of unprotected steroidal glycosides (SGs), which greatly simplifies the synthetic pathway towards ASGs and resulted in the formation of four monoacylates. Thus, it was possible to observe the enhanced cytotoxic effects of 3′-acetylated testosterone17-*O*-*β*-glucoside against seven human carcinoma cell lines (Scheme 11).

Gurung et al. (2017) [202] significantly converted curcumin to its glycosylated derivatives: **330**, **331**, **332**, and **333** via one-pot chemoenzymatic glycosylation processes. Improved water solubility and comparable antibacterial properties were demonstrated by all the products. Moreover, compared to the parent diglycoside and aglycone derivatives, **330** (AGS (IC_50_ = 7.11 μM), HCT116 (IC_50_ = 5.27 μM)) and **332** (AGS (IC_50_ = 5.86 μM), HepG2 (IC_50_ = 18.61 μM), HCT (IC_50_ = 5.40 μM), and HeLa (IC_50_ = 12.47 μM)) showed increased anticancer activity. In summary, the anticancer activity of the monoglycosides was superior to that of the diglycosides. The antitumor activity of 2-deoxyglucoside was higher than that of its corresponding oxyform among the monoglycosides (Scheme 12).

The anticancer efficacy of a number of perbutyrylated glycosides of podophyllotoxin and its derivatives was assessed in vitro. Using MTT evaluations, the majority of them demonstrated cytotoxic action against a panel of five human cancer cell lines (SMMC-7721, A-549, MCF-7, HL-60, and SW480). Three of the produced compounds, **338b**, **338e**, and **342**, demonstrated the strongest anticancer activity, with IC_50_ values ranging from 0.14 to 1.69 µM (Scheme 13) [203].

### 4.2. N-Glycosides

Using click chemistry, novel 1,3,4-thiadiazole and 1,2,3-triazole hybrid glycosides were synthesized by reacting acetylenic derivatives with various glycosyl azides, followed by deacetylation. Using the MTT assay, the cytotoxic properties of the prepared compounds were investigated against the human colorectal carcinoma HCT-116 and the human breast adenocarcinoma MCF-7 cell lines. The findings showed that the free hydroxyl glycoside **350c** and the triazole glycosides linked to *p*-methoxyarylidine derivatives **349a** and **349b** exhibited potent activity against MCF-7 human cancer cells, comparable to the reference drug doxorubicin. It was also discovered that glycoside **350c** was very potent against HCT-116 cell lines (Scheme 14) [204].

Khodair et al. (2023) [205] designed and synthesized the N-Rhodanine Glycoside Derivatives **359a–f** by the reaction of protected nitrogen nucleosides with aromatic aldehydes using morpholine as a catalyst in the presence of ethanol. The deprotected nitrogen nucleosides **359a–f** were obtained by deacetylating the protected nitrogen nucleosides **358a–f** using NaOCH_3_/CH_3_OH without cleaving the rhodanine ring. Their biological evaluation studies confirmed that the prepared compounds have shown excellent anticancer properties. Most of the compounds showed highly promising IC_50_ values when tested against HepG2, MCF-7, and A549 cancer cells. Notably, the IC_50_ values for **358a** (3.7, 8.2, 9.8 μM), **359b** (3.1, 13.7, 21.8 μM), and **359f** (7.17, 2.2, 4.5 μM) were significantly lower than those for doxorubicin (7.67, 8.28, 6.62 μM). Considering compound **359f** with Dox (IC_50_ = 9.65 and 31.27 μM), it demonstrated IC_50_ values of 7.3 and 18.2 μM for DNA intercalation and Topo II inhibition, respectively. Furthermore, compound **359f** caused HepG2 cells to undergo a 65.6-fold increase in apoptotic cell death, arresting the cell cycle in the G2/M phase (Scheme 15).

A novel series of 1,2,3-triazole glycosides was synthesized via the condensation of O-methyl allose aldehyde with a Grignard reagent followed by reaction with TMSN_3_ to afford an azide, which, on further treatment with alkynes, produced targeted compounds **363a–h**. The compounds were evaluated for anti-cancer activities towards four cancer cell lines: A549, MCF-7, HeLa, and DU145. Based on the IC_50_ values, compound **363a** was selected for further testing to prove its effects on prostate cancer cells (Scheme 16) [206].

### 4.3. S-Glycosides

Vudhgiri et al. (2017) [207] synthesized the thiadiazole-based thioglycosides **371a–j**, **372a–j** and determined their anti-cancer activities. A convergent synthetic method was used, which involved the synthesis of starting material **369** followed by the formation of **370a–j** via reaction with various acid chlorides. The acylated derivatives **371a–j** were obtained by the condensation of compound **370a–j** with imidate **367** using TMSOTf. Lastly, Zemplen deacetylation was applied to the acylated compounds **371a–j** to produce thioglycosides **372a–j**. The compounds **372e** and **372i** demonstrated significant anti-cancer activity against cervical cancer cell lines (Scheme 17).

Flefel et al. (2017) [208] afforded the thioglycoside derivatives of 1,3,4-thiadiazolyl)thiaazaspiro[4.5]decane via glycosylation using acetylated glycosyl bromides. A variety of compounds demonstrated moderate to high inhibitory activity when tested for anticancer activity against HepG-2, PC-3, and HCT116 cell lines. The most active compounds exhibited antiproliferative effects against HCT116 cancer cells, with IC_50_ values ranging from 92.2 to 120.1 nM (Scheme 18).

Khalaf et al. (2020) [209] prepared the pyridine-containing thioglycosides **389a**, **b** by the glycosylation of derivative **386** with the bromide of acetylated glycosides **387a**, **b,** followed by deacetylation at room temperature using methanolic ammonia solution. The synthesized compounds were tested against various cancer cell lines, and the results have shown that compounds **388b** and **389b** displayed prominent anti-cancer effects towards all the tested cell lines, while compound **389b** demonstrated excellent cytotoxicity against A549 and HCT116 in comparison to doxorubicin (Scheme 19).

A series of quinazoline derivatives **393** were afforded by the reaction of **390** with isothiocyanate derivatives **391**. Under anhydrous alkaline and glycoside conditions, **393** were reacted with alkyl halides and bromides **395** to carry out S-alkylation and S-glycosylation, respectively. Based on molecular docking studies, several derivatives (**396c**, **396g**, **397c**, and **397a**) were identified as promising anti-cancer agents due to their better binding affinities towards the EGFR tyrosine kinase. In vitro analysis revealed that the derivatives exhibited effective anti-proliferative activity with lower IC_50_ values, particularly **397a**, which had IC_50_ values of 2.09 μM against MCF-7 and 2.08 μM against HepG2 cell lines (Scheme 20) [210].

### 4.4. Miscellaneous

Khodair et al. (2019) [211] developed and synthesized the N- and S-glycosides of the polyfunctionalized pyridine-biphenyl system attached to the benzotriazole molecule. A simple, efficient, and high-yielding method was employed to synthesize the N-glycosides of pyridine-2-one derivatives **400** and the S-glycosides of pyridine-2-thione derivatives **402a–h**. Using the MTT assay on the MCF7 cell line, the anticancer properties were evaluated in relation to standard cisplatin. The compounds with the highest cytotoxic effect on MCF7 were **401f**, **401g**, **402f**, and **402h**. The increased expression of apoptotic genes Bax and p53, coupled with the decreased expression of the anti-apoptotic gene BCL2, indicated that the anticancer effects of these four drugs induced apoptosis. Furthermore, the nontoxic dosages of the examined compounds were assessed in Hep2, MA104, BGM, FRHK4, and Vero cells; S-Glycoside derivatives were found to be more active than N-glycosides (Scheme 21).

The Click reaction, also known as Cu(I)-Catalyzed Azide-Alkyne Cycloaddition (CAAC), was used to create novel 1,2,3-triazole glycosides substituted with acridine and benzothiazole. Under click reaction conditions, the corresponding 1,2,3-triazole acetylated N-glycoside products were obtained by reacting the alkyne derivatives with glycopyranosyl azides. In addition, novel tetrazolyl glycosides linked to the benzothiazole ring system were created. The findings also showed that benzothiazolyl-based triazole glycosides are highly active compounds. The designed products were tested against several tumor cell lines, including human colon (HCT116), hepatocellular carcinoma (HepG2), human lung carcinoma (A549), and breast (MCF-7) cancer cell lines. Overall, the majority of the targeted compounds demonstrated outstanding efficacy against each type of tumor cell (Scheme 22) [212].

Glycosylation of 1,3,4-thiadiazolethiol compounds produced new 1,3,4-thiadiazole thioglycosides attached to substituted pyrimidines **424**, **430**. The anti-cancer properties of the prepared derivatives were examined in vitro using human cell lines HepG-2 and MCF7. All of the investigated substances exhibited dose-dependent moderate cytotoxic activities against all cell types (Scheme 23) [213].

El-Sofany et al. (2022) [214] investigated the anti-cancer potential of some novel imidazole-triazole-based glycosides. The intended compounds were prepared via a multistep synthetic pathway. The starting material **431** was prepared and then reacted with various alkyl halides and propargyl bromide to obtain the corresponding compounds **433** and **441a, b**. The 1,2,3-triazole-*N*-acetylated glycosides **405a**, **b** were produced from compound **433** when it was allowed to react under the click reaction conditions. These glycosides then interacted with a methanolic ammonia solution to produce free hydroxyl N-glycosides **436a**, **b**. Additionally, compounds **441a**, **b** and sodium azide reacted to form azido derivatives **442a**, **b**, which in turn reacted under the conditions of a click reaction to form glycosides **444a–d**. The free hydroxyl thioglycosides **445** can be obtained by deacetylating the molecules **444a–d**. Moreover, the thioglycoside derivatives **439a–c** of **431** were obtained by treatment with acetylated glycosyl bromides **437** followed by deacetylation using ammonia-methanol solution. All newly developed substances were tested on the human breast cancer cell lines MCF-7 and MDA-MB-231. The MTT assay was used to evaluate cytotoxicity, and IC_50_ was calculated. Compounds **445a**, **439b**, and **436a** were shown to have the strongest cytotoxic effects on the two cancer cell lines (Scheme 24).

El-Sayed et al. (2022) [215] elaborated the synthesis and anti-cancer activities of coumarin-based triazole-glycosyl hybrid and their thioglycoside derivatives. Compounds **449b**, **449d**, **451**, and **454a** showed higher activities against various human cancer cells via the activation of mitochondrial apoptotic pathways when the cytotoxic activity of the produced compounds was tested against a range of human tumor cell lines. Comparing the **449b** and **449d** to the reference drugs Sorafenib, Erlotinib, and Roscovitine having IC_50_ values of 1.58 ± 0.11, 0.18 ± 0.05, and 0.46 ± 0.30 µM, respectively, the compounds showed excellent inhibitory activity (IC_50_ values of 0.22 ± 0.01, 0.93 ± 0.42 and 0.24 ± 0.20 µM, respectively, for compound **449b**) and (IC_50_ values of 0.12 ± 0.50, 0.79 ± 0.14 and 0.15 ± 0. 60 µM, respectively, for compound **449d**). In addition, the encouraging enzymatic results were confirmed by the molecular docking simulation, which further demonstrated the well-fitting behavior of compounds **449b** and **449d** in the active sites of CDK-2/cyclin A2 kinases, EGFR, and VEGFR-2, as well as their suitable binding patterns (Scheme 25).

Libnow et al. (2008) [216] have reported the synthesis of indirubin-N′-glycosides as potential anti-cancer agents. First, L-rhamnose **456** was coupled with aniline **457** in ethanol and was further acetylated with acetic anhydride in pyridine to give N-glycosyl aniline **458**. The compound **458** obtained was then cyclized by AlCl_3_ treatment with oxalyl chloride to give the desired N-glycosyl isatin **459**. The reaction of N-glycosyl isatin **459** with indoxyl derivative **460** in the presence of Na_2_CO_3_ led to condensation to give the corresponding indirubin-N′-glycoside **461a**. Other indirubin-N′-glycoside derivatives **461b** were obtained by deacetylation and functionalization. The synthetic indirubin-N′-glycosides **461a–b** were then screened for anti-proliferative activity against 5637 (bladder carcinoma), A-427 (small-cell lung cancer), KYSE-70 (esophageal cancer), and MCF-7 (breast cancer) human cancer cell lines. The compound **461a** exhibited significant and selective activity against MCF-7 cells with an IC_50_ value of 0.76 ± 0.16 µM. The compound **461a** was moderately active against 5637, A-427, and KYSE-70 cancer cell lines with IC_50_ values of >12.5 µM. The anti-proliferative activity of indirubin-N′-glycoside was generally greater than that of the non-glycoside indirubin aglycone, suggesting that a glycosyl group could considerably improve the anticancer activity of the indirubin scaffold (Scheme 26).

Tiwari et al. (2024) [217] used microwave-assisted click chemistry to elaborate on the synthesis and anticancer properties of triazole-bridged N-glycosides of pyrazolo[1,5-*a*]pyrimidinones. Compound **464b** had exceptional anticancer activity against MDA-MB231 cells with an IC_50_ value of 27.66 mM, while compound **466f** demonstrated noteworthy inhibitory potential against MCF-7 cells with an IC_50_ value of 4.93 mM. The experimental results were confirmed by in silico docking studies, which demonstrated the potential of these triazole-bridged N-glycosides as effective anti-cancer drugs (Scheme 27).

In this study, the synthesis and anti-cancer properties of Pd and Cu metal complexes with N-aryl fluorinated O-glycoconjugate Schiff base ligands that include CF_3_ and *α*-D-glucose groups were elaborated. Pd(L1G)_2_ **470** was the most cytotoxic of the synthesized complexes against a panel of six cancer cell lines, including PC-3, MCF-7, HCT-15, SK-LU-1, K-562, and U-251. It also showed less activity against non-cancerous cells, emphasizing the role of carbohydrate moieties in improving selectivity toward malignant cells. Pd complexes containing glycosylated ligands demonstrated greater DNA-binding affinity through improved hydrogen bonding interactions, according to molecular docking studies, suggesting their potential as selective anticancer drugs (Scheme 28) [218].

Abbas et al. (2024) [219] described the synthesis and anticancer properties of novel sulfonamide-triazole-glycoside hybrids. Compounds **474a** and **474b** showed promising activity against HepG-2 and MCF-7 cancer cell lines, with IC_50_ values ranging from 8.39 to 16.90 μM against HepG-2 and 19.57–21.15 μM against MCF-7, comparable to doxorubicin (IC_50_ = 13.76 ± 0.45 and 17.44 ± 0.46 μM, respectively). Compounds **474a** and **474b** demonstrated strong inhibition of VEGFR-2 (IC50 = 1.33 and 0.38 μM), carbonic anhydrase hCA IX (IC_50_ = 66 and 40 nM), and hCA XII (IC_50_ = 7.6 and 3.2 nM), in comparison to sorafenib (IC_50_ = 0.43 μM) and SLC-0111 (IC_50_ = 53 and 4.8 nM, respectively). These derivatives’ binding affinities to their target enzymes were revealed by molecular docking, which supported their potential for additional modification in the manufacturing of anticancer drugs (Scheme 29).

Srour et al. (2025) [220] developed the synthesis and anticancer properties of novel N-glycosyl heterocyclic scaffolds. **479a** outperformed erlotinib (IC_50_ = 2.07 ± 0.07 and 5.14 ± 0.33 µM) with IC_50_ values of 1.64 ± 0.11 and 5.00 ± 0.51 µM for HepG-2 and HCT-116 cancer cell lines, respectively, and showed a safe profile against normal BJ-1 cells. Compounds **479a** and **479b** had significant inhibitory action in EGFR inhibition experiments, with IC_50_ values of 0.069 ± 0.003 and 0.075 ± 0.003 µM, respectively, similar to erlotinib (0.048 ± 0.002 µM). Molecular docking revealed compounds **479a** and **479b** as possible anti-cancer drugs that target EGFR kinase (Scheme 30).

Five synthetic analogs of the cardiac glycoside proscillaridin A have been designed by Somani et al. (2025) [221]. Triacetate **482** showed similar potency to proscillaridin A, whereas acetonide **481** demonstrated strong antiproliferative activity in colorectal, hepatocarcinoma, and ovarian cancer cells, with IC_50_ values in the single- to double-digit nanomolar range in SK-OV-3 cells. Because of their high logP values, silylated analogs **485** and **483** showed decreased activity. Acetonide 5 significantly decreased the NF-κB and STAT signaling pathways and inhibited the pro-cancer markers CD117 and CD133 while enhancing cleaved caspase-3. These results show that specific glycosylation changes can be tolerated while maintaining anticancer efficacy (Scheme 31).

This study demonstrates the formation and anticancer properties of pyridine-thiazole hybrids and their derived 1,2,3-triazole analogs with different sugar moieties. Compounds **491a–491c** demonstrated better CDK-2/cyclin A2 inhibition (IC_50_ = 0.26, 0.22, and 0.18 μM) than roscovitine (0.42 μM), while pyridine-thiazolidinone-1,2,3-triazole-based glycosides **491b** and **491c** showed equipotent EGFR inhibitory activity (IC_50_ = 0.12 and 0.15 μM) than erlotinib (IC_50_ = 0.15 μM). By upregulating caspase-3, Bax, and Bcl-2, triazole glycoside **491c** caused apoptosis in MCF-7 cells (Scheme 32) [222].

El Malah et al. (2025) [223] presented the synthesis and anticancer properties of new 1,2,3-triazole-N-glycosides with a benzoquinoline moiety. The LDH assay was used to assess the synthetic compounds’ cytotoxic activities against the cancer cell lines HepG-2, MCF-7, and HCT-116. Compounds **494** and **495** showed good safety profiles against normal BJ-1 cells and similar cytotoxic effects to doxorubicin, especially against HCT-116 cells. Molecular docking correlated the observed activity with the ability of these compounds to form stable protein–ligand interactions with biological targets (Scheme 33).

The synthesis and anti-cancer properties of a new class of pyridine-based glycosides with a 1,2,4-triazole core and their 1,3,4-oxadiazolyl equivalents with different sugar segments were evaluated. Compounds **499** and **503** exhibited excellent anticancer activity against HepG-2 and MCF-7 cell lines with IC_50_ values of 10.6 and 12.8 μM against HepG-2 and 8.3 and 9.8 μM against MCF-7, respectively. While oxadiazolyl-glycoside **503** showed better CDK-2/cyclin A2 inhibition (half the potency of roscovitine) than its triazole counterpart **499**, both drugs displayed EGFR inhibitory action similar to erlotinib. According to mechanistic research, compound **503** enhanced the levels of p53 and Bax in MCF-7 cells while causing apoptosis and cell cycle arrest (Scheme 34) [224]. The synthetic glycosides discussed above, together with their anticancer mechanisms, are summarized in Table 6.

## 5. Critical Appraisal of Anticancer Glycoside Studies

### 5.1. Plant-Derived Glycoside Studies

Though there is a significant amount of literature reporting the anticancer effects of plant glycosides, there are a number of methodological problems that need to be taken into account.

#### 5.1.1. Lack of Normal Cell Controls

From the 151 plant-derived glycosides included in this review, only a few were tested on non-malignant control cell lines. These include oleandrin 5 (IC50 = 0.56 μM for normal colonic epithelial cells vs. 0.02 μM for SW480 cancer cells), peruvoside 6 (no cytotoxicity at active concentration in normal cells, glucoevatromonoside 8 exhibited no toxicity towards normal lung MRC-5 fibroblasts or PBMCs at specific concentrations, Proscillaridin A 19 (from Drimia maritima extract showed IC50 = 13.1 µg/mL in normal fibroblasts compared to 0.9–2.3 µg/mL in colorectal cancer cells, and digitoxin 4 was examined against normal human bronchial epithelial cells compared to gefitinib-resistant NSCLC H1975 cells having IC50 = 0.19 µM. Resultantly, the therapeutic potential of many reported glycosides remains ambiguous.

#### 5.1.2. Methodological Concerns of the Cytotoxicity Assessment

The MTT assay is one of the most commonly used assays for cytotoxicity, but it can yield false-positive results, such as the ability of redox-active molecules to interfere with the assay, and it relies on cellular metabolic activity instead of directly measuring cell viability. Thus, confirmation by additional assays, such as resazurin reduction, ATP measurement, clonogenic assays and flow cytometry analysis, can support biological findings. In most of the papers, IC_50_ values were not always accompanied by confidence intervals or detailed statistical analysis, thus restricting direct comparison of potency for the various compounds.

#### 5.1.3. In Vivo Validation Gap

A few of the reviewed studies went beyond in vitro cancer cell models to animal-based validation. In addition, although effective growth inhibition of cancer cells in vitro is not a guarantee of therapeutic activity in vivo, a variety of pharmacokinetic factors can affect the therapeutic effects, such as oral bioavailability, plasma protein binding, metabolic stability, tissue distribution, maximum tolerated dose, excretion, and tumor microenvironment. However, all of the above studies are lacking, which is a critical gap in translational development.

#### 5.1.4. Serum Protein Binding Consideration

Cardiac glycoside binding to serum albumin is a significant factor that diminishes the free drug concentration, thereby greatly affecting the anticancer activity of cardiac glycosides. Few studies of plant glycosides included the effect of physiological serum protein amounts on anticancer activity, despite evidence that albumin binding can considerably influence the free concentration of cardiac glycosides.

#### 5.1.5. Future Studies Recommendations

Future studies of plant glycosides should address the following minimum criteria for reporting: (i) include at least one cell line of normal human tissue of the same origin as the cancer cell line used; (ii) use two or more orthogonal viability assays; (iii) report IC_50_ values with 95% confidence intervals; (iv) include a clinically relevant positive control (e.g., doxorubicin, cisplatin, paclitaxel); and (v) test, if applicable, serum protein binding when translation to the in vivo environment is intended.

### 5.2. Marine-Based Glycoside Studies

The marine glycosides reviewed here contain many substances with well-documented cytotoxicity in cell models of cancer, while data on the effects of these substances on non-cancerous cells is far more variable. This constraint is especially relevant if highly potent compounds are to be considered for further development, since a compound’s potency in the context of cancer cells does not constitute therapeutic selectivity.

This is true in the case of angucycline glycosides from actinomycetes. Mormomycin B (**152**), saquayamycin B1 (**153**) and saquayamycin B (**154**) were all shown to have submicromolar activity against breast cancer cell lines MDA-MB-231, MCF-7 and BT-474, with IC_50_ values of 0.16 to 0.67 µM. But similar data on cytotoxicity against appropriate non-malignant breast epithelial cells were not reported, making it difficult to assess the selectivity of the agents towards malignant cells. Likewise, urdamycin W (**159**) had potent growth-inhibitory activity against multiple cell lines with GI_50_ values in the 0.019–0.104 µM range, but the cytotoxicity results against normal cells were not described in the cited paper.

Several of the glycosides derived from sea cucumbers show a similar restriction. In the reviewed study, echinoside B (**156**) and 24-dehydroechinoside B (**157**) were tested against Ehrlich ascites carcinoma cells, but no reports were found on their activity against non-malignant cells. The potent cytotoxicity of Coloquadranoside A (**158**) was observed against a wide range of human tumor cell lines; however, there were no corresponding data on normal cells for determining tissue-specific selectivity. Most of the other glycosides isolated from sea cucumbers have similar limitations; for instance, the anticancer activity has been claimed mainly for the following glycosides based on the growth of malignant cell models: hillaside C (**162**), philinopside E (**163**), intercedensides A–C (**167a–b**, **168**), and holothurinosides A and C (**171–172**).

Not all marine glycosides lack normal-cell evaluation; however, non-malignant cell models have been used in some studies and have shown that selectivity can be studied in candidates derived from the marine environment. However, this type of assessment is not completely uniform throughout the literature on marine glycosides, especially for those with high or wide-range cytotoxicity. The introduction of tissue-relevant normal-cell models in combination with quantitative selectivity indices and mechanistic validation would significantly aid in prioritizing marine glycosides for continued anticancer development.

### 5.3. Synthetic-Based Glycoside Studies

The pattern of normal-cell evaluation for the synthetic glycosides is more variable. There are several synthetic series that have used non-malignant cell models in their biological characterization, and have shown that structural modification and glycosylation can be correlated with an improved differential response between the malignant and non-malignant cells. Normal-cell testing is not consistent, however, throughout the synthetic glycosides included in this review.

For the purposes of comparative selectivity assessment, the phenolic glycoside esters **277a–n** and **278** can serve as a good example. The antiproliferative activity against cancer cell lines and the low level of cytotoxicity towards normal cells, with IC_50_ values > 100 µM, were shown by these compounds. In this series, compound **277f** exhibited IC_50_ values of 4.43 ± 0.35 μM against MCF-7 cells and 6.13 ± 0.64 μM against HL-60 cells, with a significant difference between the responses of the malignant and normal cells under the conditions reported here. The indole glycosides **460a–l** and **461a–l** were also found to be active against breast cancer cell lines, such as MDA-MB-231, MCF-7 and MDA-MB-453, without any significant toxicity against the normal breast epithelial cell line (MCF-10A) at the concentrations that were tested. The use of MCF-10A is especially informative as it will provide a tissue-relevant non-malignant comparator in breast cancer models. Other synthetic series of glycosides have also used non-cancerous cells. The normal fibroblast models were used to assess pyridine–thiazole–triazole glycosides and benzimidazole–triazole glycoconjugates; further evidence of differential cytotoxicity between malignant and non-malignant cells was gained. Similarly, certain metal glycoconjugates with activity against non-cancerous cells exhibited decreased activity compared to cancer cell lines. These studies all show that the use of suitable normal-cell controls can yield valuable insights in the identification of increased anticancer activity from non-specific cytotoxicity.

While all these are promising examples, the evaluation of normal cells is not a regular occurrence in the synthetic glycoside literature. Some synthetic series presented in this review were screened mainly or solely for their inhibitory activity against cancerous cell lines, such as 8-hydroxyquinoline glycoconjugates (**240a–d** and **241a–d**), diosgenin glycosylated derivatives (**257a–i**), dihydroartemisinin-O-glycosides (**268a**), and some triazole-based glycosides. Therefore, the high potency values reported for individual synthetic derivatives may not necessarily be taken as a sign of increased therapeutic potential if non-malignant controls were not used.

Another issue is selecting the model for the normal cells. Fibroblast cell lines, e.g., BJ-1, can serve as a valuable tool for general information about cell cytotoxicity, but may not match the normal counterpart of the tissue in which the cancer model was derived. Possibly, tissue-matched nonmalignant human cells (such as MCF 10A for breast cancer, BEAS 2B for lung cancer, and appropriate normal colonic epithelial models for colorectal cancer) give a more informative basis for evaluating tissue-specific selectivity.

## 6. Structure-Activity Relationships of Anticancer Glycosides

Structural properties that control the anticancer potency are crucial for rational drug design. Thereafter, an analysis is presented combining the data for natural and synthetic glycosides discussed above.

### 6.1. Aglycone Determinants

#### 6.1.1. Steroidal Aglycones

For Na^+^/K^+^-ATPase inhibition, the presence of an unsaturated 5-membered lactone ring at C-17 is required among the cardenolide-type aglycones. The binding affinity is lowered when the pyrone ring is replaced with a six-membered pyrone ring (bufadienolides); this is demonstrated by comparing digitoxin **4** with bufalin **1**. The C-14*β* hydroxyl group is involved in hydrogen bonding with the ATPase pocket residue Glu111, which is why the removal of this hydroxyl group (as in 14-deoxy analogs) leads to a greater drop in potency.

#### 6.1.2. Flavonoid Aglycones

The B-ring with a catechol group (3′,4′-dihydroxy substitution) increases the radical scavenging and pro-apoptotic activity of the flavonoid glycosides. Quercetin glycosides **54–56** (catechol) and **60–62** (catechol) exhibit lower IC_50_ values against the same cell lines compared to the kaempferol glycosides (catechol with 4′-hydroxy). The presence of the 4′-hydroxy group, as in isorhamnetin glycosides **49–53**, **58–59**, partially maintains the activity, although it loses activity as compared to quercetin.

#### 6.1.3. Triterpene Aglycones

For activation of the mitochondrial apoptosis pathway, the carboxylic acid group at C-30 of oleanane-type triterpenes (in glycyrrhizin **98** and saponariosides **112–114**) must be present. Esterification or amidation of this carboxyl group (as in synthetic derivatives **95a–97**) lowers the activation of caspase-3, but does not affect initial uptake by the cells.

### 6.2. Sugar Chain Effects

#### 6.2.1. Chain Length

Based on the analysis of glycosides, which differ from each other only in length of the sugar chain, a common trend was observed: The shorter the sugar chain, the more permeable the membrane and the less selective the compound is for cancer cells versus normal cells. The longer chains and trisaccharide counterparts have permeability and activity that are less than their disaccharide counterparts.

#### 6.2.2. Deoxy Sugars

In the presence of 2,6-dideoxy sugars (digitoxose, cymarose, oleandrose), the cytotoxicity is greater than that of glycosides containing 2,6-dihydroxy sugars (glucose) with the same aglycones. This is due to greater lipophilicity and to less recognition by efflux transporters like P-glycoprotein. Convallatoxin **7** (deoxy sugars) is more potent in lung cancer cells than its glucoside analog.

#### 6.2.3. Sugar Stereochemistry

The configuration of the anomeric center at the glycosidic bond is always the *β*-form, with the *α*-form of cardiac glycosides showing less inhibition of Na^+^/K^+^-ATPase. This stereospecificity is due to the orientation of the 3′-hydroxy group, as it forms an important hydrogen bond with the ATPase residue Glu117 in *β*-anomers, whereas in *α*-anomers it hydrogen bonds with other residues.

### 6.3. Synthetic Modifications That Increase Activity

Some synthetic modifications that increase activity are discussed below.

#### 6.3.1. Acylation

Synthetic glycosides with 3′-*O*-acetylation are more potent than the parents; for example, derivatives **262a**, **338b**, and **338e** are more potent than the parent compounds. The acetyl group would enhance membrane partitioning and would also make the compound less susceptible to hydrolysis by *β*-glucosidase. Perbutyrylated podophyllotoxin glycosides **338b** and **338e** have the best profile among the acylated derivatives.

#### 6.3.2. Triazole Linkers

The click chemistry-derived 1,2,3-triazole-linked glycosides (**308a–b**, **312a–d**, **349a–b**, **350c**, **363a–h**) show improvement in anticancer activity while maintaining high metabolic stability. The triazole ring is known to be resistant to glycosidase and is a non-hydrolyzable bioisostere of the natural *O*-glycosidic bond. The compound **349b** (triazole-linked thiadiazole glycoside) is more potent than its *O*-glycosidic analog against MCF-7 cells.

#### 6.3.3. Metal Complexation

The formation of metal complexes with flavonoid glycosides (such as the oxidovanadium-diosmin complex 63) makes them more effective against cancer than the basic glycoside. The metal center selectively releases the ROS, taking advantage of the increased basal oxidative stress in cancerous cells. This approach has not yet been employed for cardiac glycosides and is an underutilized area of opportunity.

## 7. Conclusions and Future Directions

In this review, we have examined the therapeutic potential of anti-cancer glycosides derived from plant, marine, and synthetic sources. Glycosides, with their diverse structures and mechanisms of action, represent a promising class of compounds in the fight against cancer.

A total of **224** glycosides from natural sources and more than **40** synthetic glycosides showing anti-cancer properties were assembled by chemical classes. Although some glycosides were only evaluated for their anti-cancer activity against a single cancer type, some compounds were tested against a variety of cancers, showing their potential to be the best anti-cancer agents. For example, in plant-derived steroid glycosides, the compound Strophanthidin **22** showed potential against lung, breast, and liver cancer. Similarly, the cardiac glycosides Calotropin **24** and Calotoxin **25** and compounds **28–29** displayed activity towards breast, lung and colorectal cancer cells. Among the polyphenol glycosides, the compounds **49–54** showed potent activities against lung, colon, liver, and breast cancers. The flavonoid glycosides named Tiliroside, Isorhamnetin-3-*O*-glucosid, Isorhamnetin-3-*O*-rutinoside, Rutin, and Spiraeoside **57–61** demonstrated anti-cancer potential in breast, colon, and liver cancer cell lines. The compounds Solamargine **64** and Solasonine **65** were found to be active against a variety of cancer types such as ovarian, skin, lung, colon, breast, leukemia, pancreatic, and gastric cancer. Moreover, in the plant-derived terpene glycosides, the substances **95–98** showed promising anti-cancer potential towards lung, colorectal, ovarian, colon, and cervical cancer cells. Amygdalin **133** and Linamarin **134**, the cyanogenic glycosides, were proven to be active anticancer agents against prostate, breast, colorectal, ovarian, and bladder cancer types. Marine glycosides, such as those derived from sea cucumber, possess unique structures and potent cytotoxic effects, making them valuable candidates for drug development. All of the discussed synthetic glycosides displayed prominent anticancer potential against a variety of cancer cells, including some more potent derivatives such as **268a**, **277f**, **318b**, **338b**, **338e**, **342**, **359f**, **388b**, **389b**, **397a**, **449b**, and **449d,** having low IC_50_ values.

Various studies have reported several mechanisms of action for the anti-cancer activity of glycosides. A complete summary of the pathways targeted by these compounds is given in Figure 1. It is suggested that these mechanisms must be studied in order to develop potent drugs with a number of significant benefits, including a lower chance of resistance and a broad range of action. Keeping in view the reduced side effects and lower toxicity, studies of toxicity in human cell lines need to be carried out for the most promising anti-cancer substances. It should be noted that only a few compounds were tested towards normal human cell lines, which include compounds **5**, **6**, **277a–n**, **278**, **460a–l**, and **461a–l**.

The structure–activity analysis in Section 5 not only summarizes anticancer activity but also identifies a number of drug-like properties that are crucial for the development of glycosides in the future. While the relatively large molecular weight and polarity of many glycosides can limit conventional drug-likeness, sugar-chain length and deoxygenation affect membrane permeability and cancer-cell selectivity. Therefore, rational synthetic and semisynthetic modification should be used to address these structural trade-offs. Another significant limitation is metabolic stability. While more stable bioisosteric linkers, like 1,2,3-triazoles, may increase resistance to enzymatic degradation without compromising anticancer action, native O-glycosidic bonds are susceptible to hydrolysis by β-glucosidase. Furthermore, in vitro potency could overestimate physiologically attainable activity due to serum protein binding, especially for cardiac glycosides, which might lower the free drug concentration in vivo. A further challenge is the limited translation of in vitro findings to in vivo and clinical settings. Most of the 224 natural and more than 40 synthetic glycosides reviewed have been evaluated mainly in cell-based models, with limited information on bioavailability, tissue distribution, pharmacokinetics, maximum tolerated dose, and in vivo efficacy. Therefore, thorough cytotoxicity evaluation using normal-cell controls, suitable reference medications, statistical reporting, serum protein-binding investigations, and pharmacokinetic and animal validation should all be included in future research. Acylation, triazole bioisosteres, and metal complexation are among the strategies previously discussed in this review that offer helpful methods for enhancing stability, selectivity, and drug-like behavior. These strategies may help advance promising glycosides toward the development of clinically relevant anticancer candidates.

## Figures and Tables

**Figure 1:**
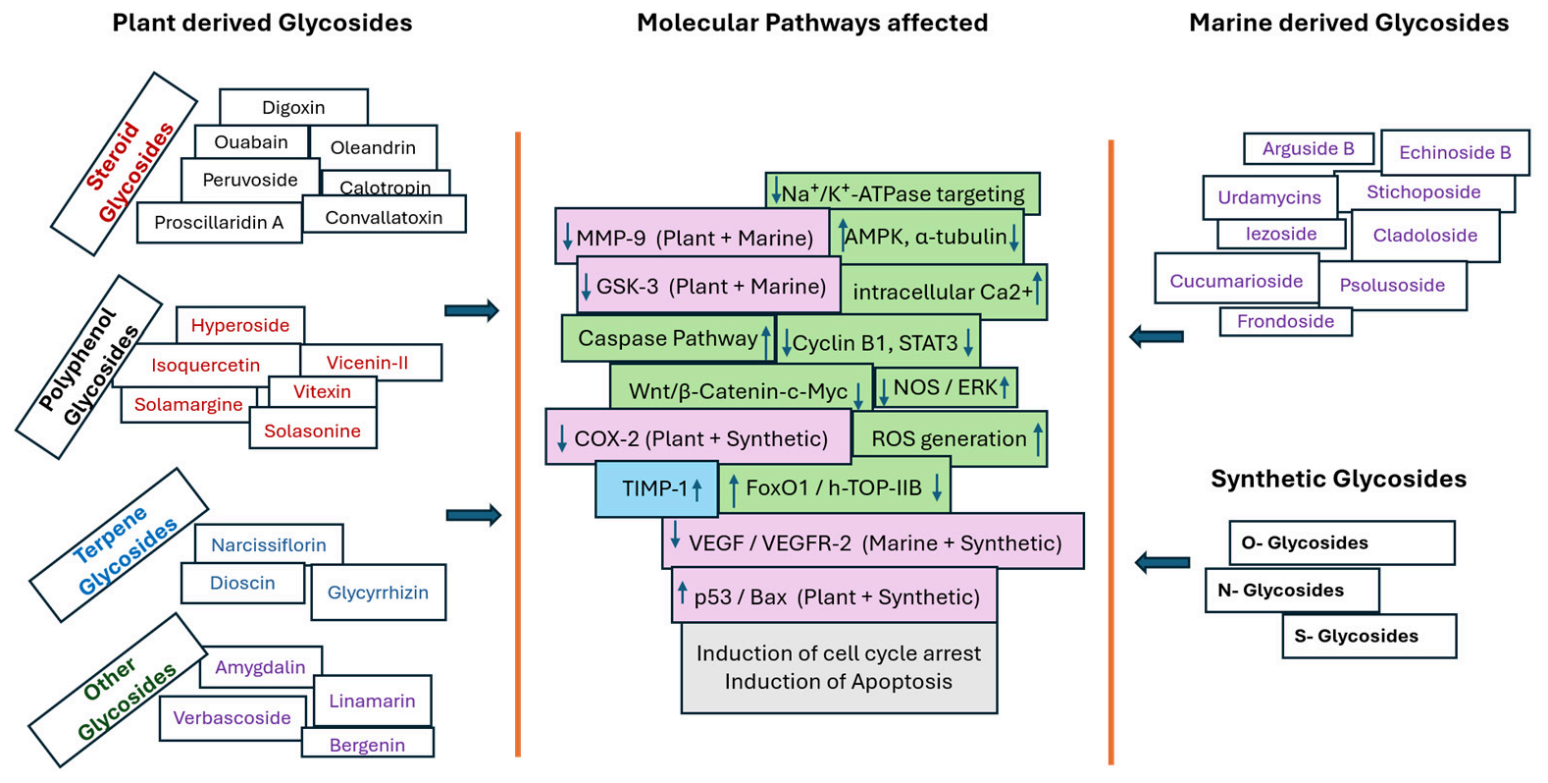
Illustration of various pathways targeted by glycosides discussed in the present review, color-coded by source: green = plant-derived only, blue = marine-derived only, purple = shared across two or more source classes, gray = universal endpoints (cell cycle arrest, apoptosis) common to all classes. ↑, increasing; ↓, decreasing.

**Figure 2:**
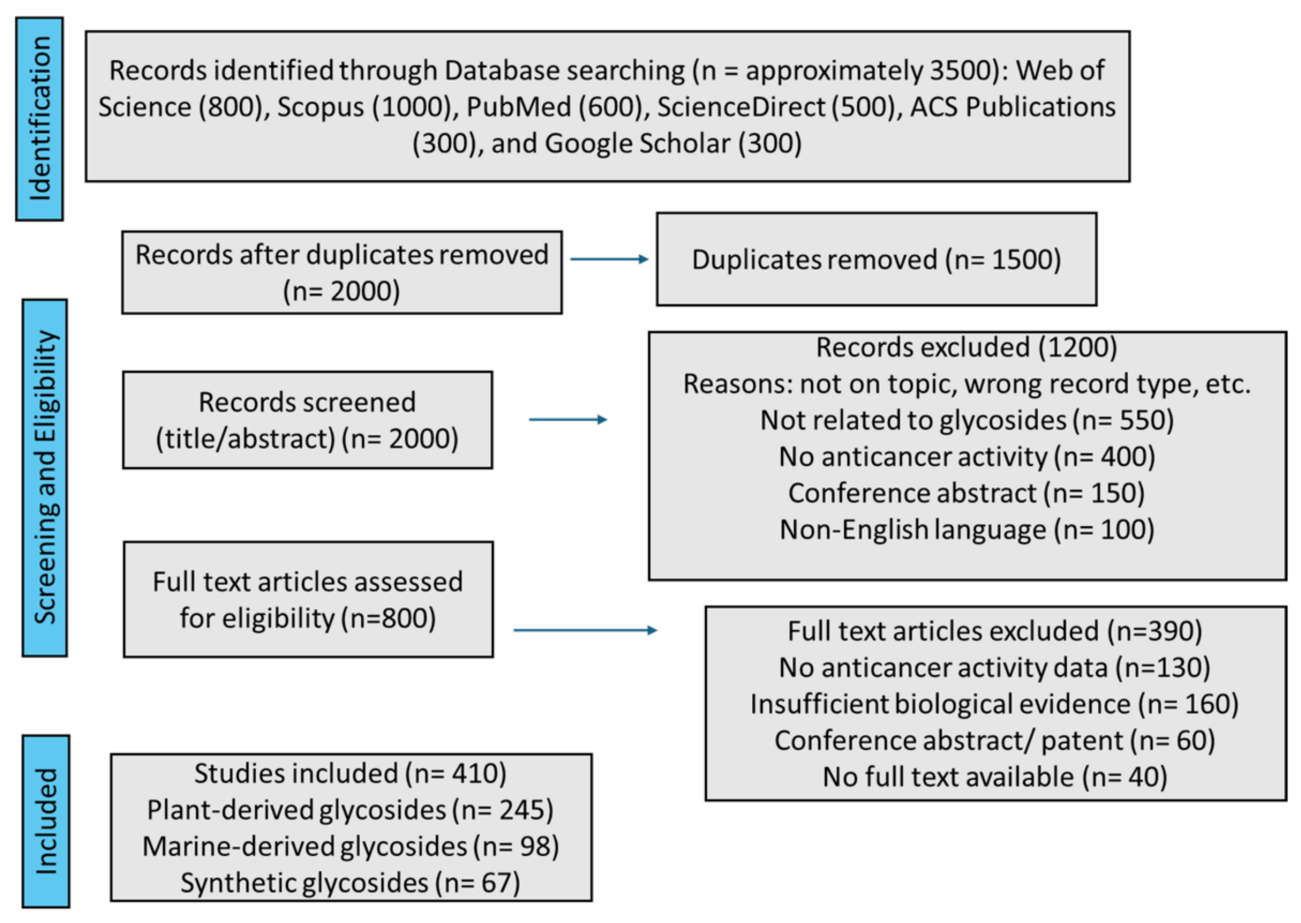
PRISMA flowchart of the systematic literature search and study selection for anticancer glycosides.

**Figure 3:**
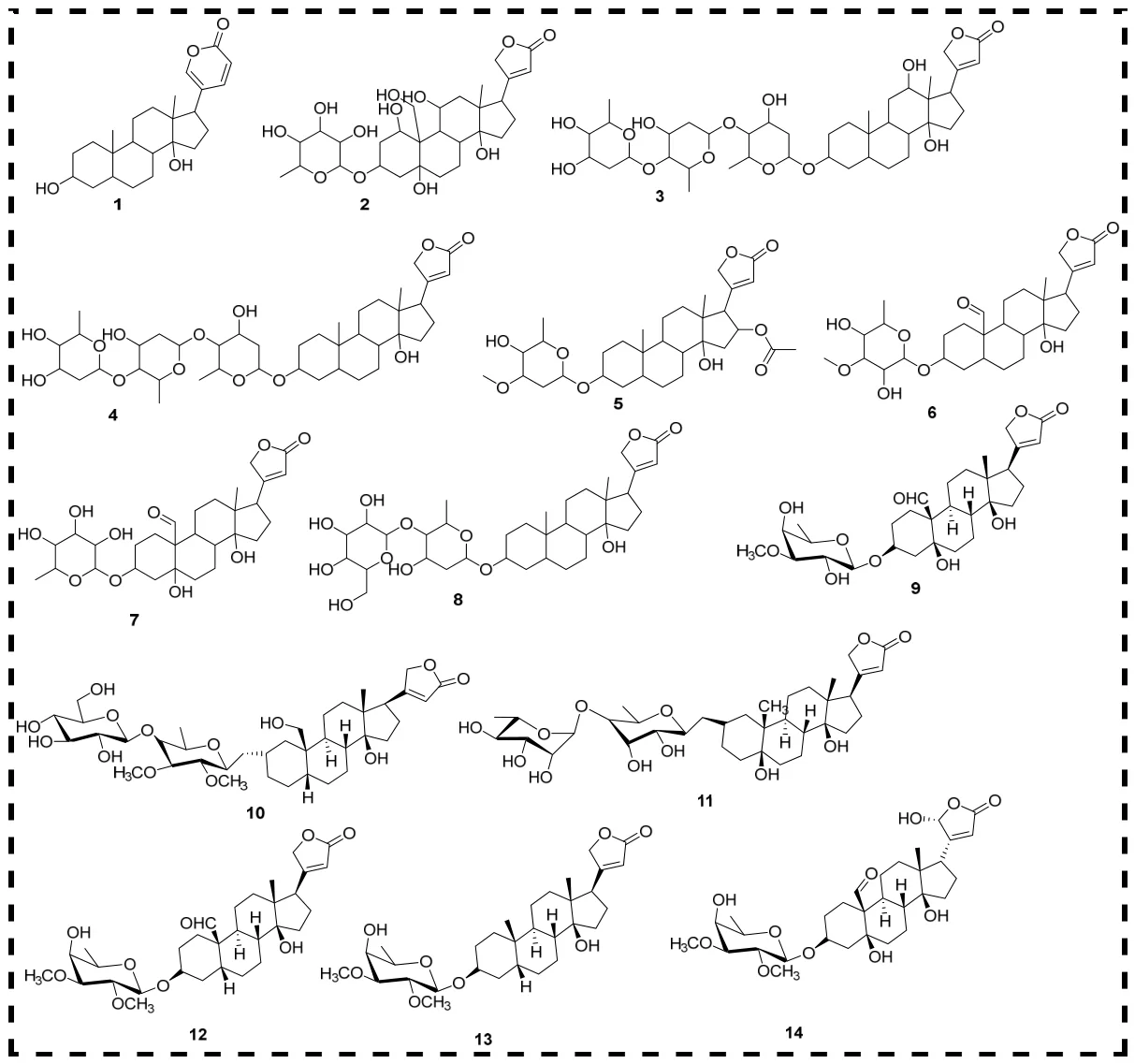
Anticancer steroid glycosides (Part 1).

**Figure 4:**
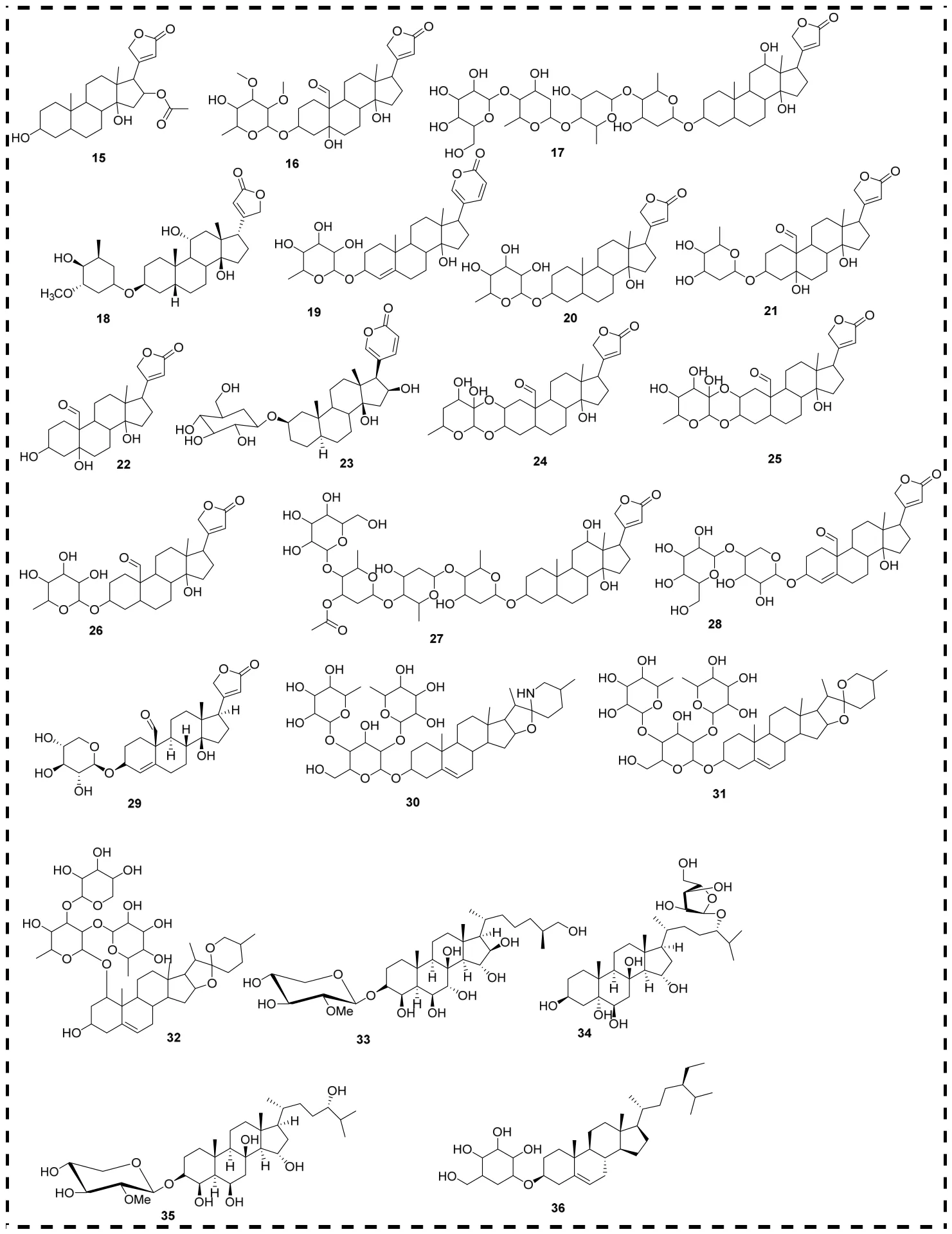
Anti-cancer steroid glycosides (Part 2).

**Figure 5:**
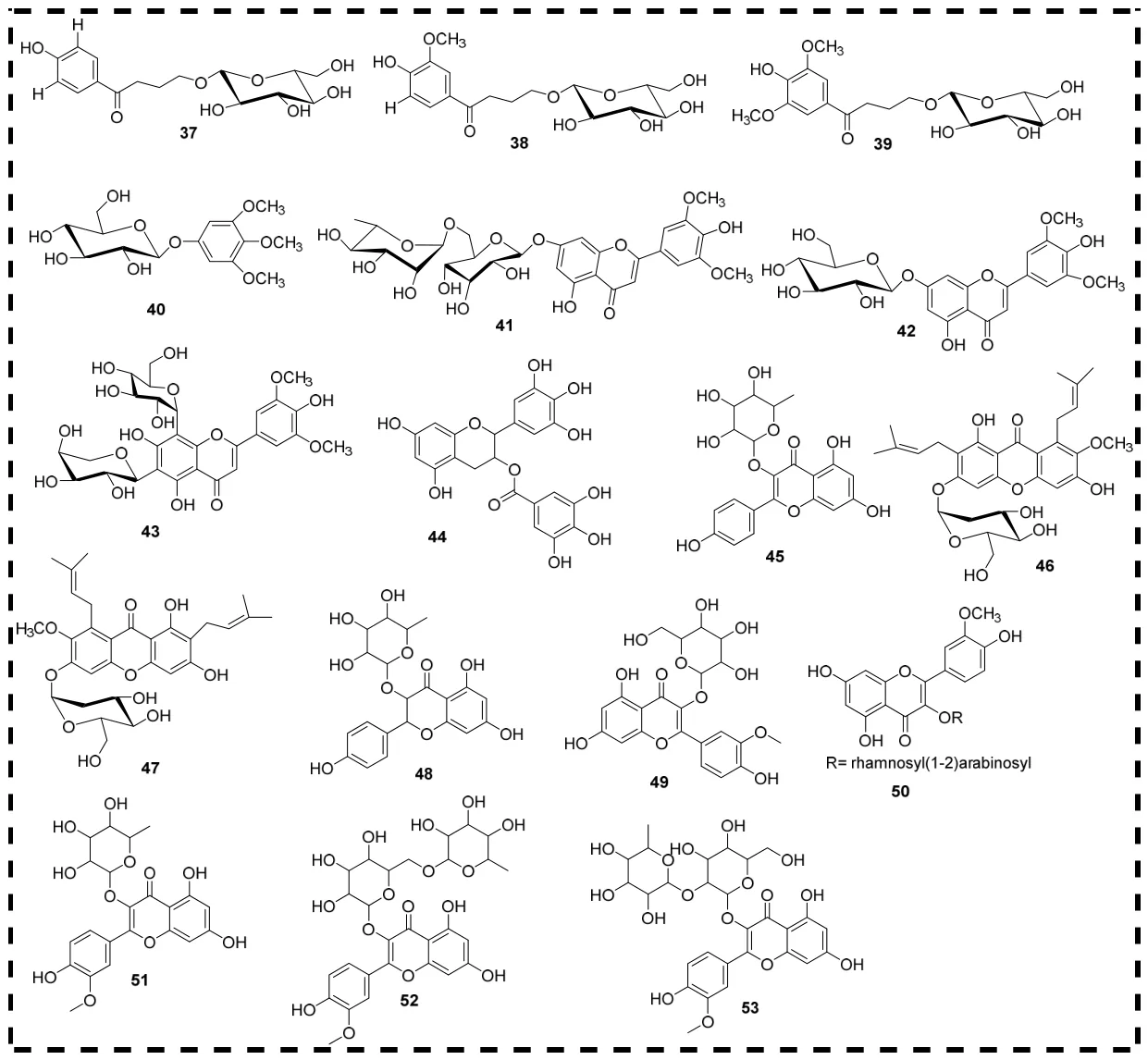
Anti-cancer Polyphenol glycosides (Part 1).

**Figure 6:**
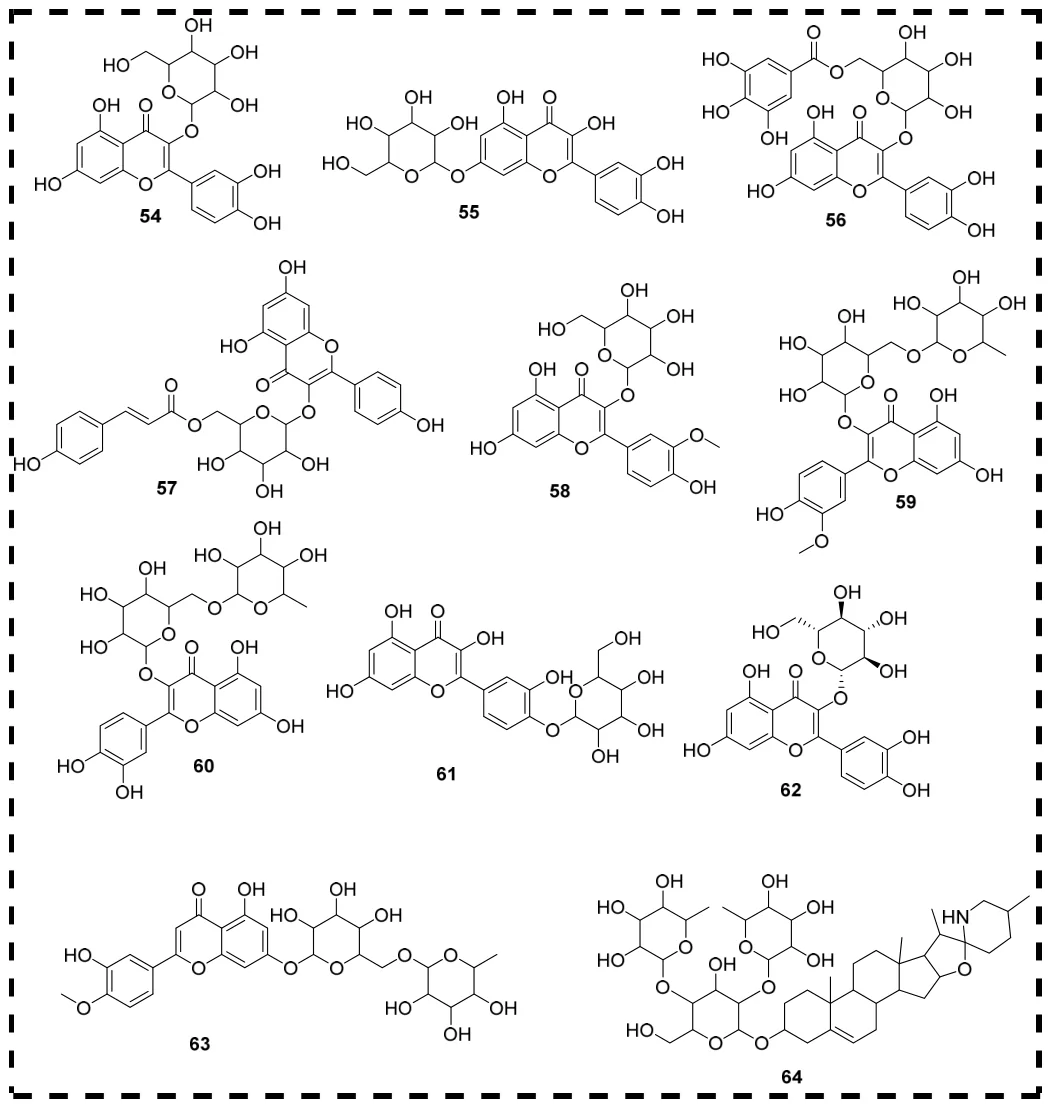
Anticancer polyphenol glycosides (Part 2).

**Figure 7:**
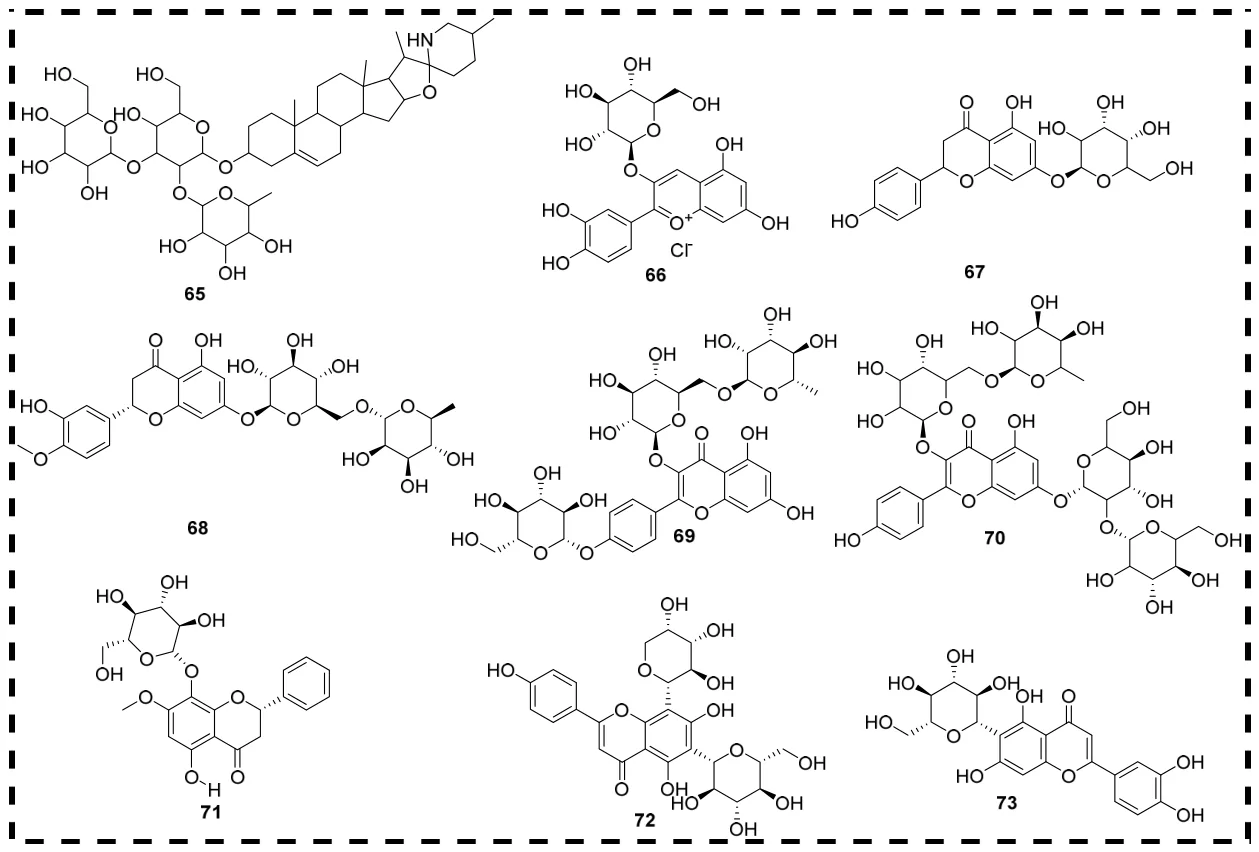
Anti-cancer Polyphenol glycosides (Part 3).

**Figure 8:**
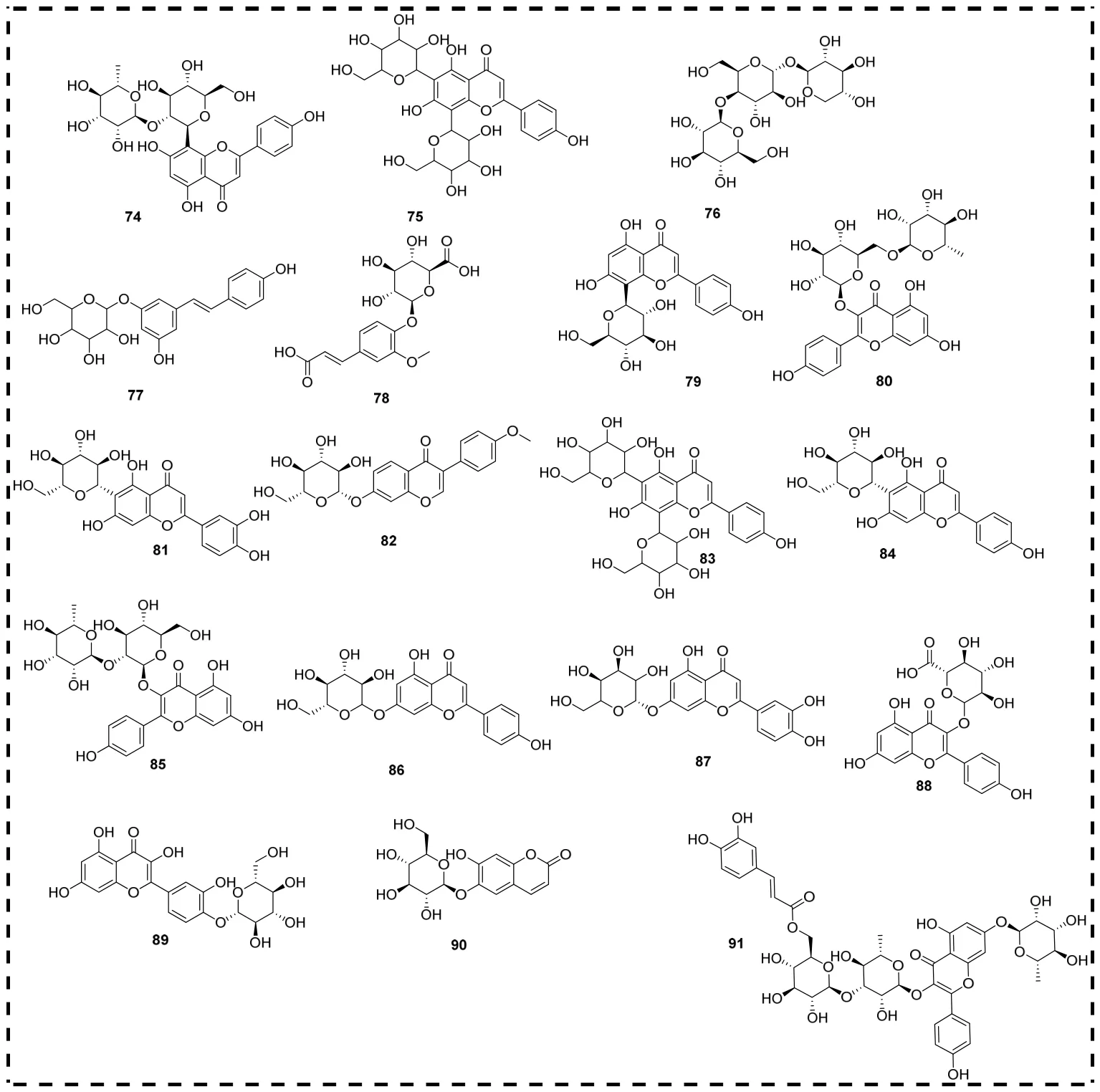
Anti-cancer Polyphenol glycosides (Part 4).

**Figure 9:**
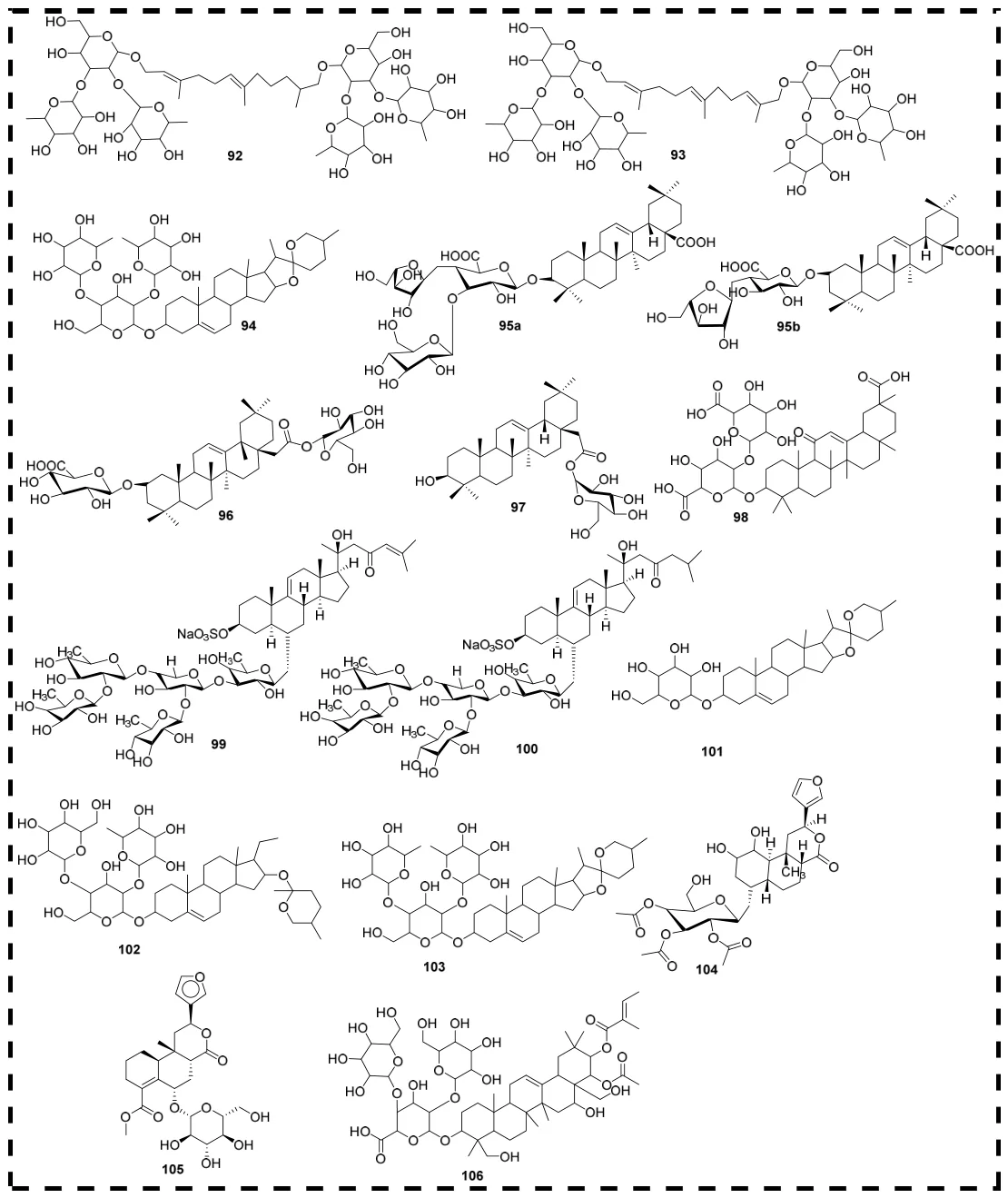
Anti-cancer terpene glycosides (Part 1).

**Figure 10:**
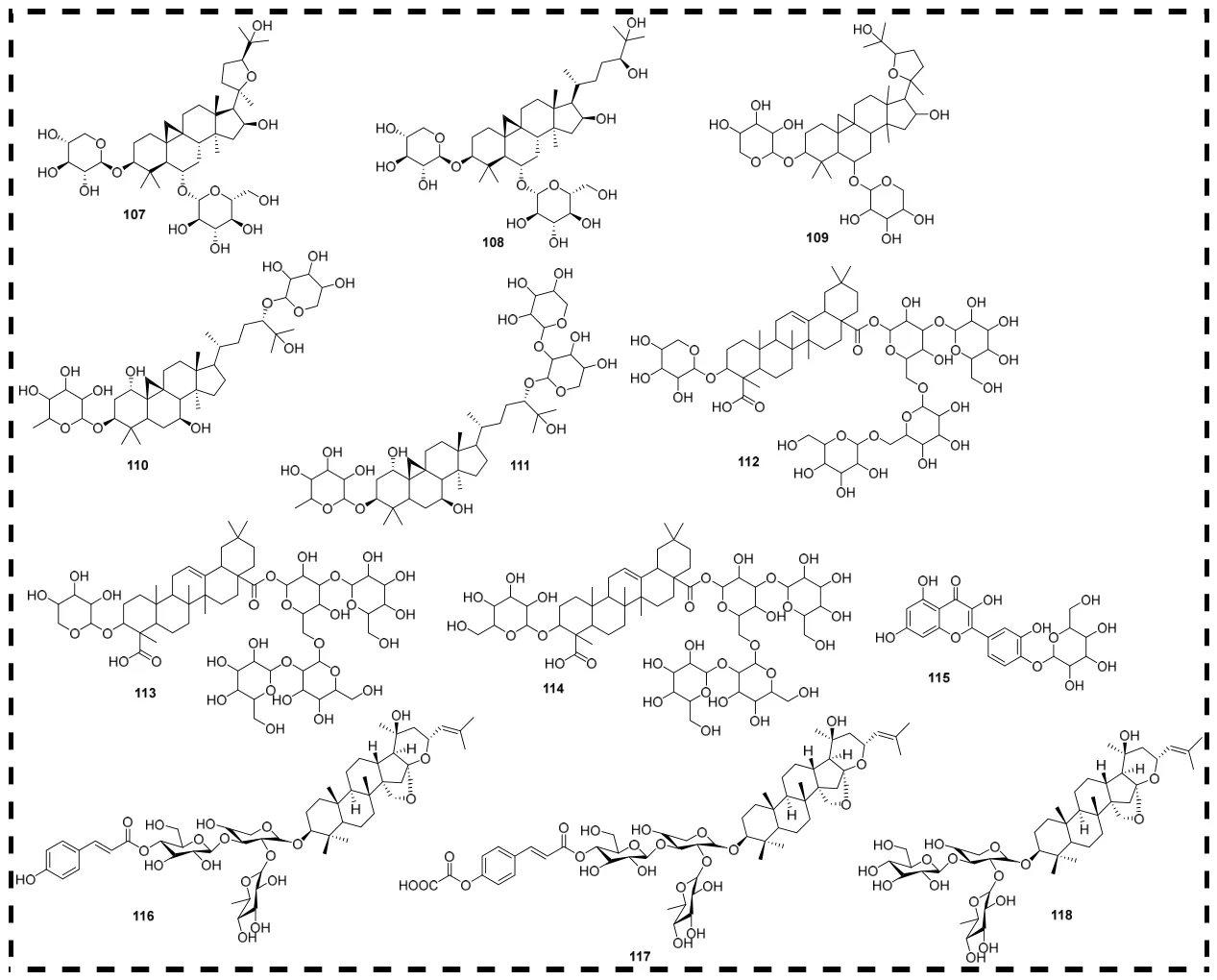
Anti-cancer terpene glycosides (Part 2).

**Figure 11:**
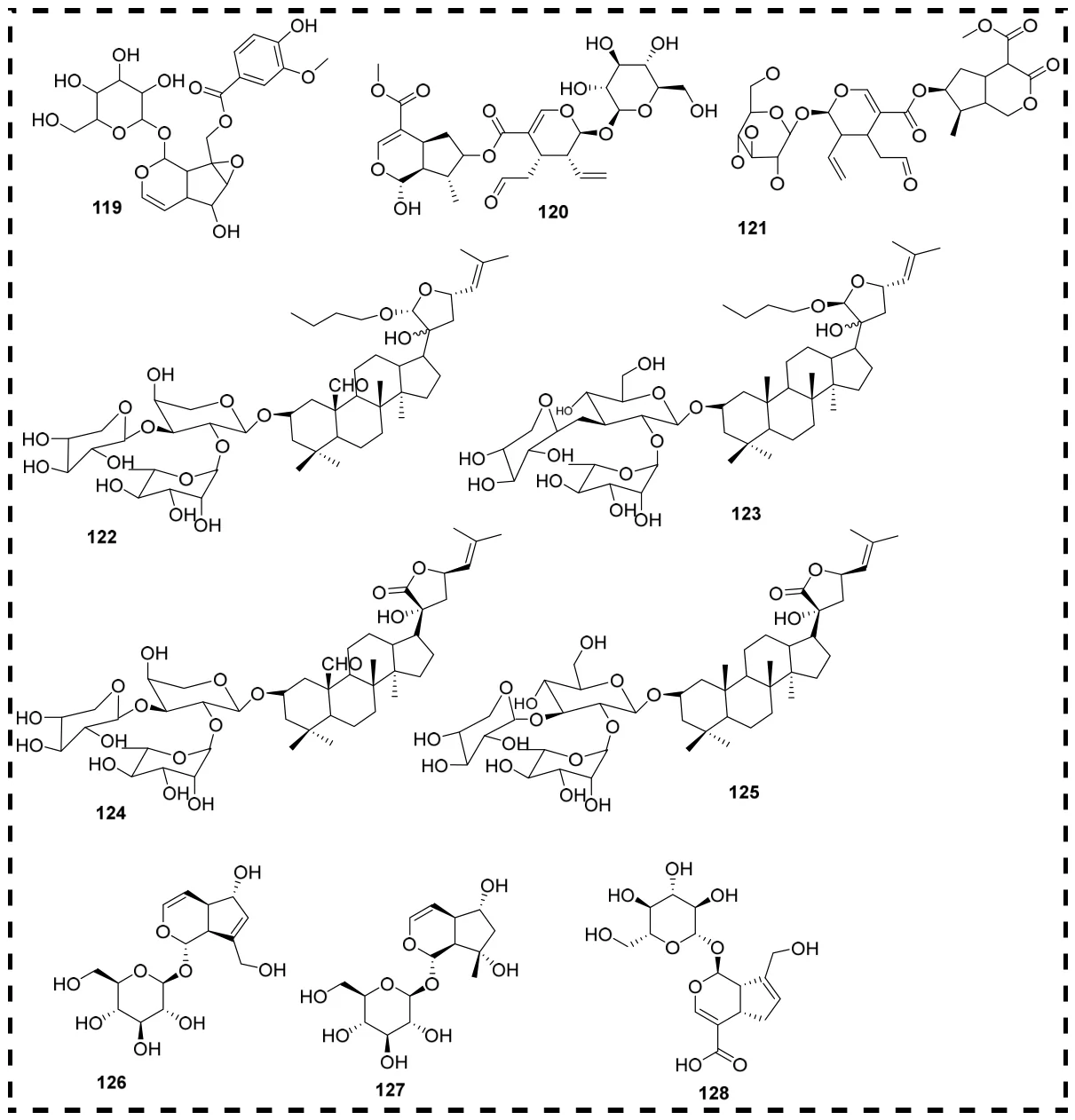
Anti-cancer terpenoid glycosides.

**Figure 12:**
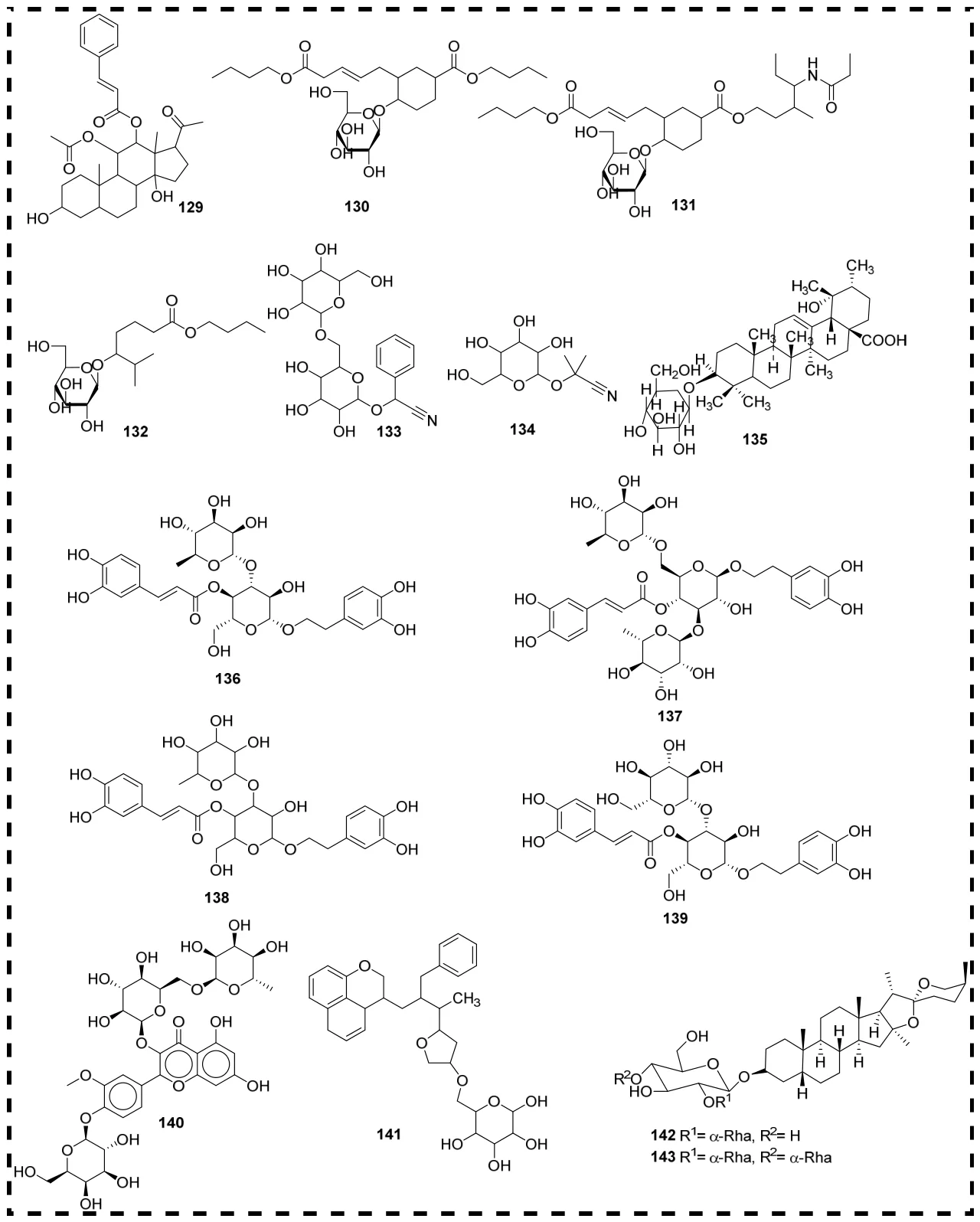
Other glycosides having anti-cancer properties (Part 1).

**Figure 13:**
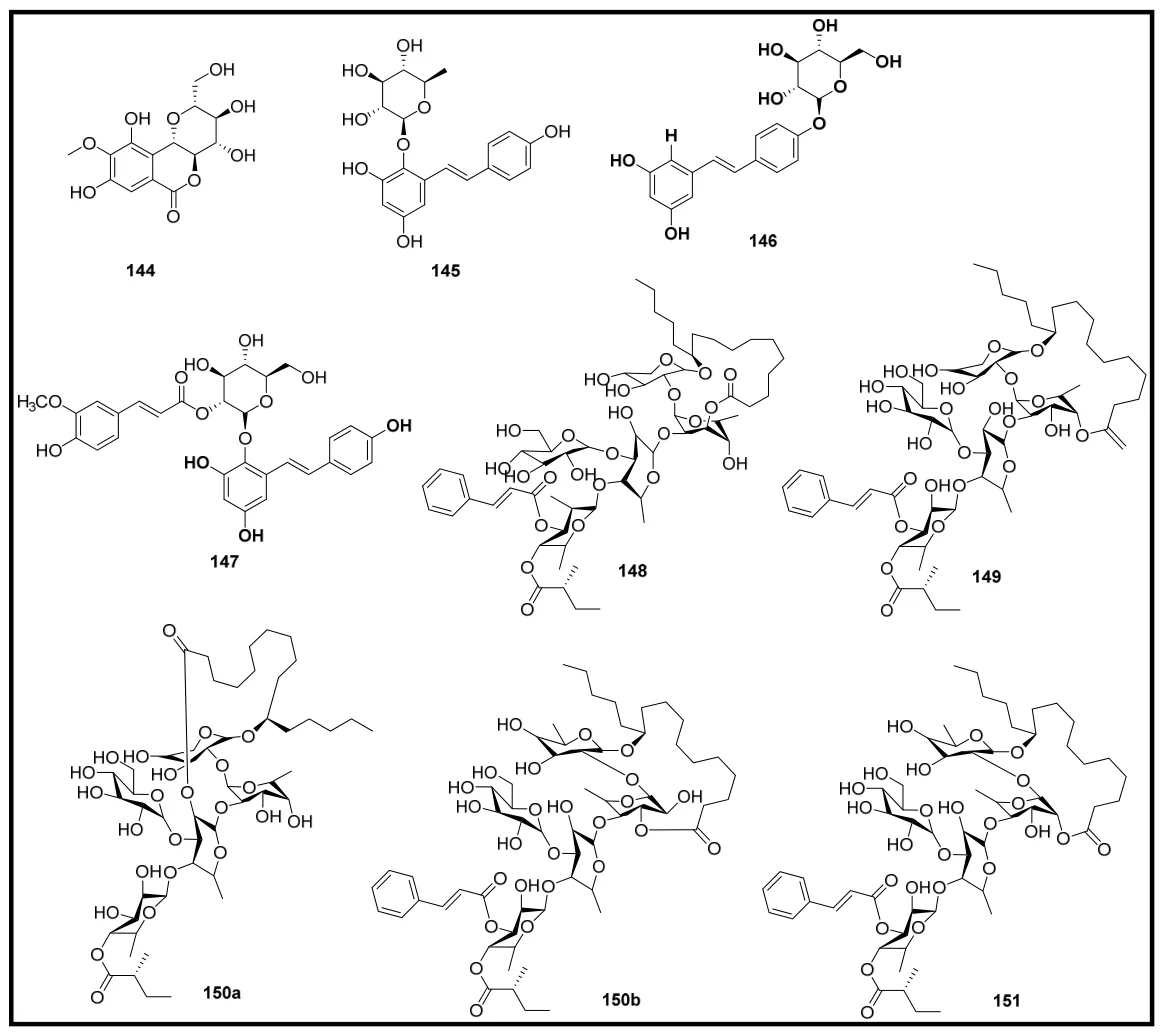
Other glycosides having anti-cancer properties (Part 2).

**Figure 14:**
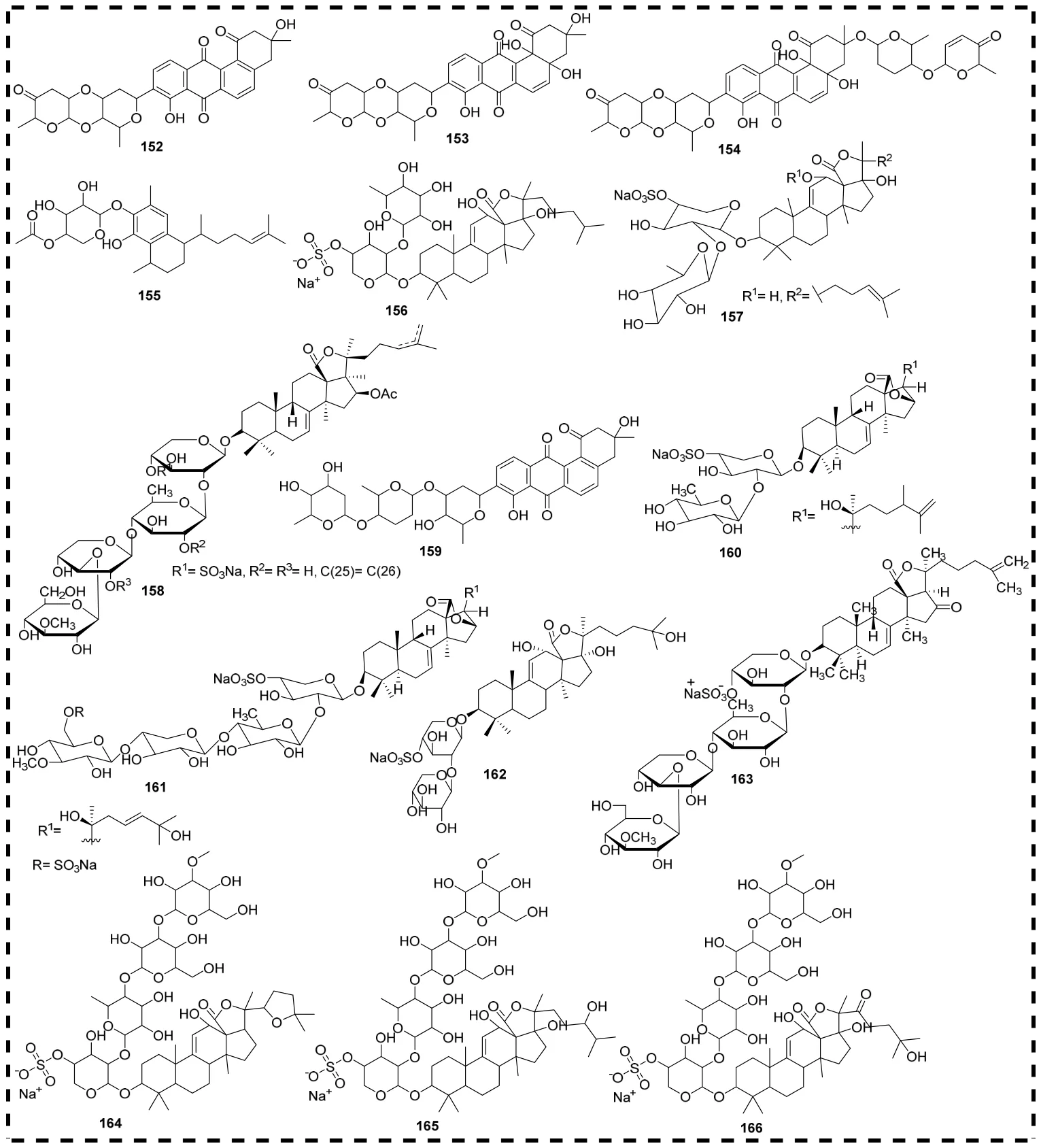
Marine-derived anti-cancer compounds (Part 1).

**Figure 15:**
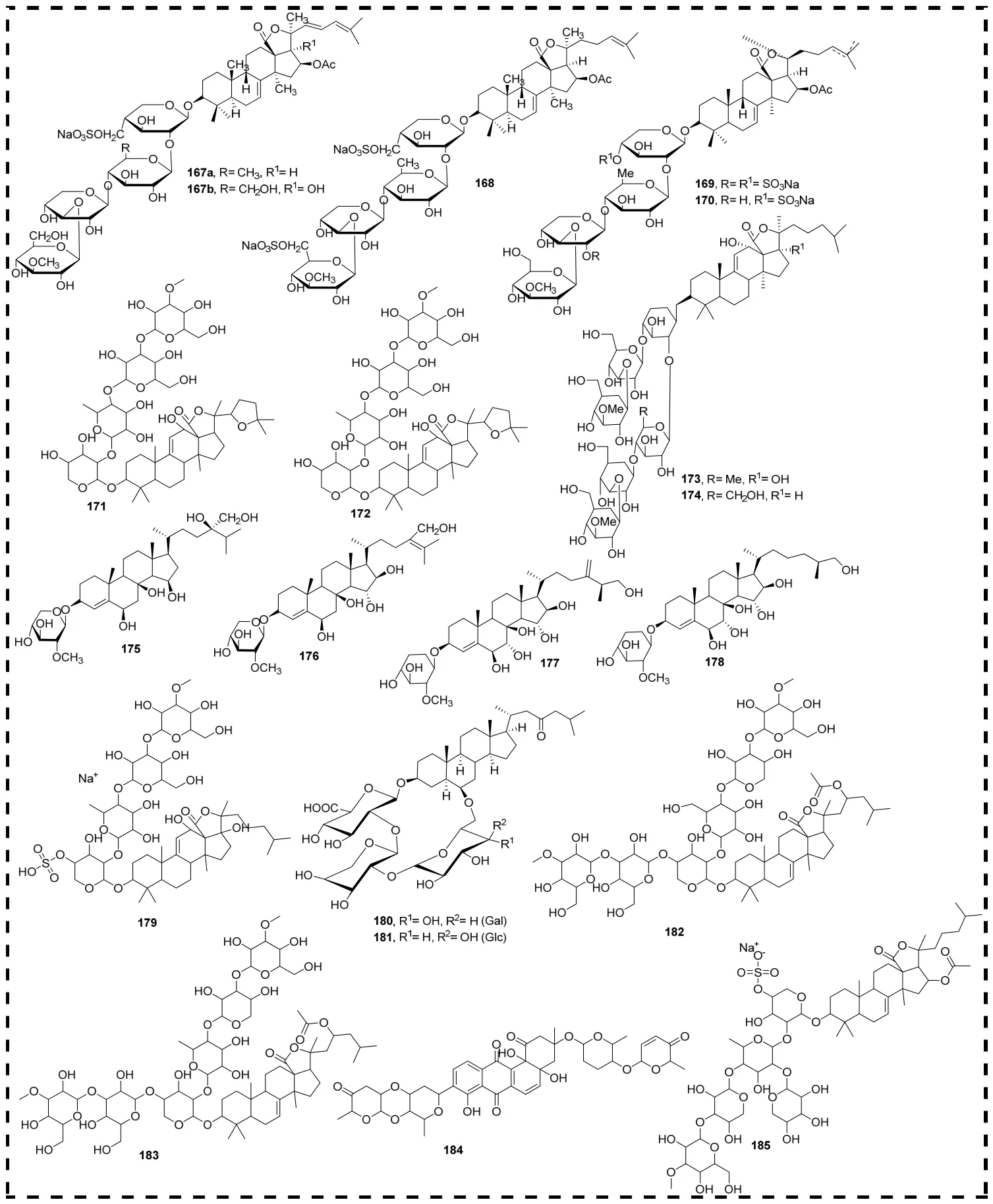
Marine-derived anti-cancer compounds (Part 2).

**Figure 16:**
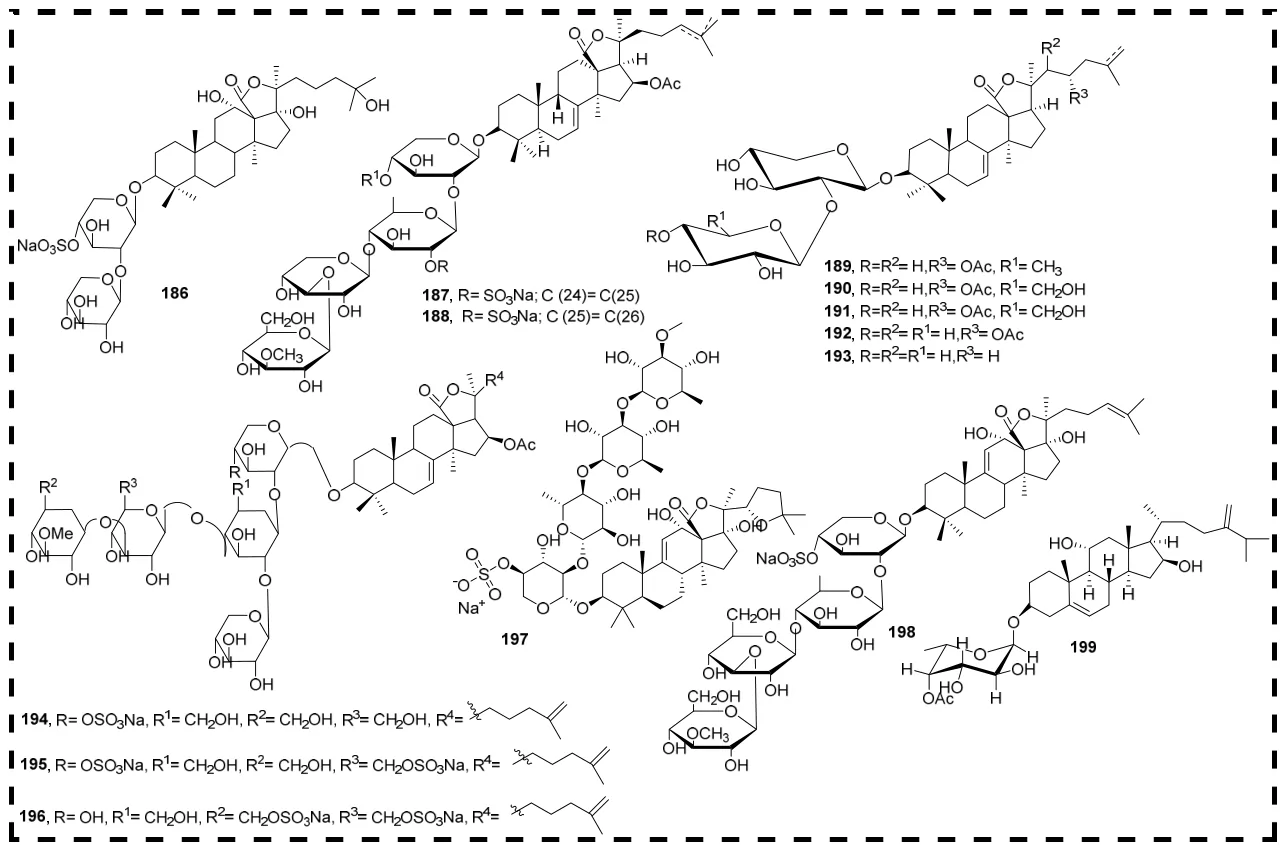
Marine-derived anti-cancer compounds (Part 3).

**Figure 17:**
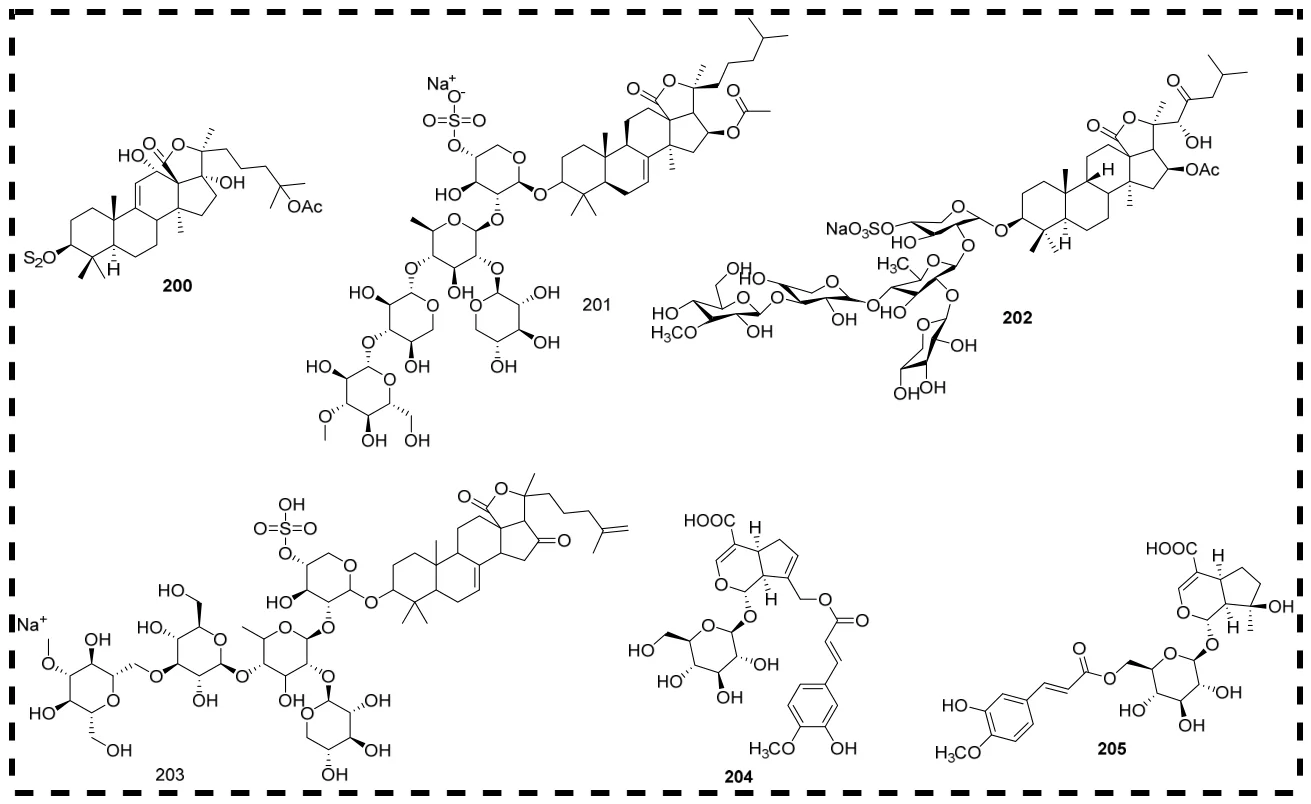
Marine-derived anti-cancer compounds (Part 4).

**Figure 18:**
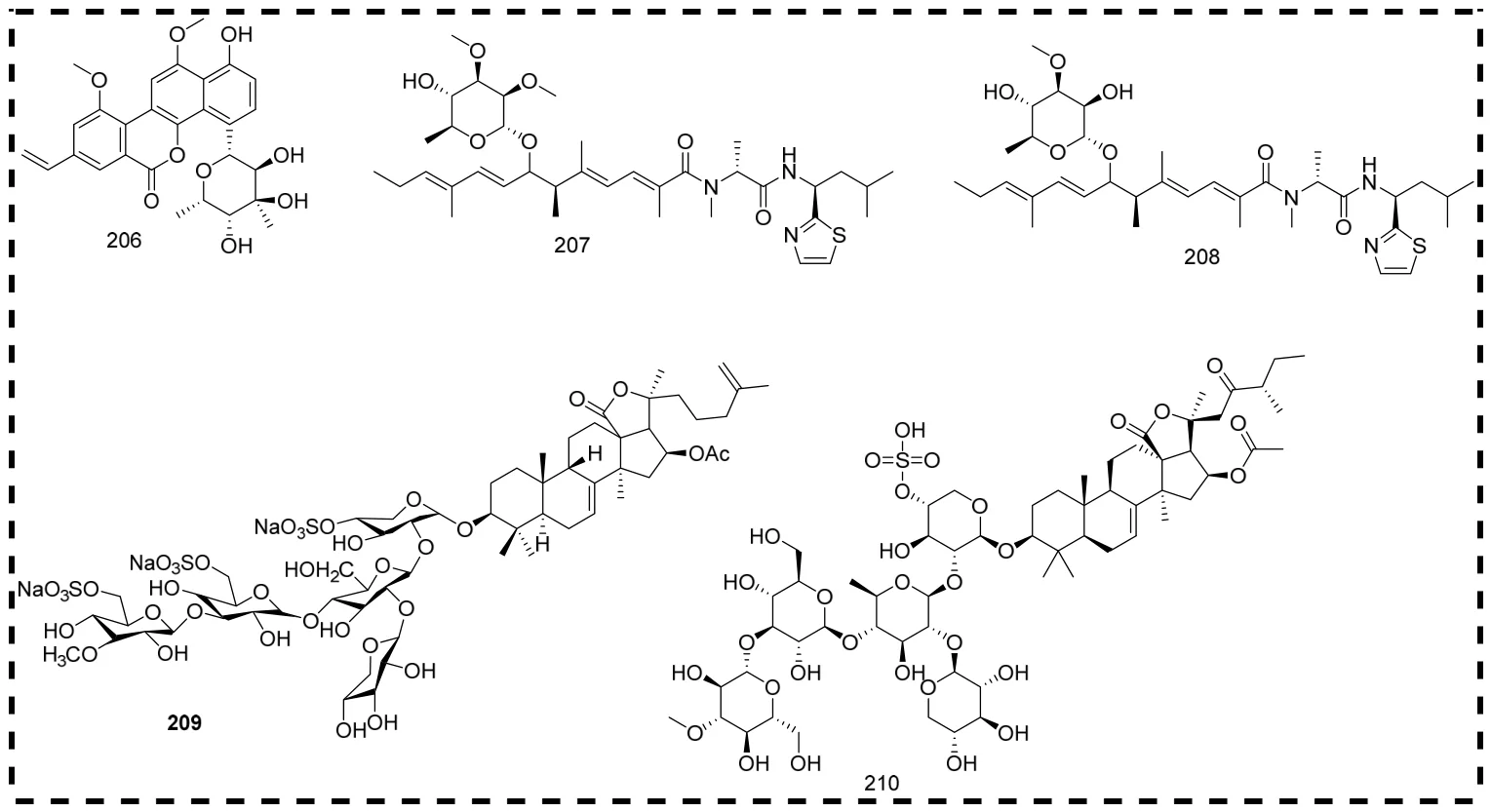
Marine-derived anti-cancer compounds (Part 5).

**Figure 19:**
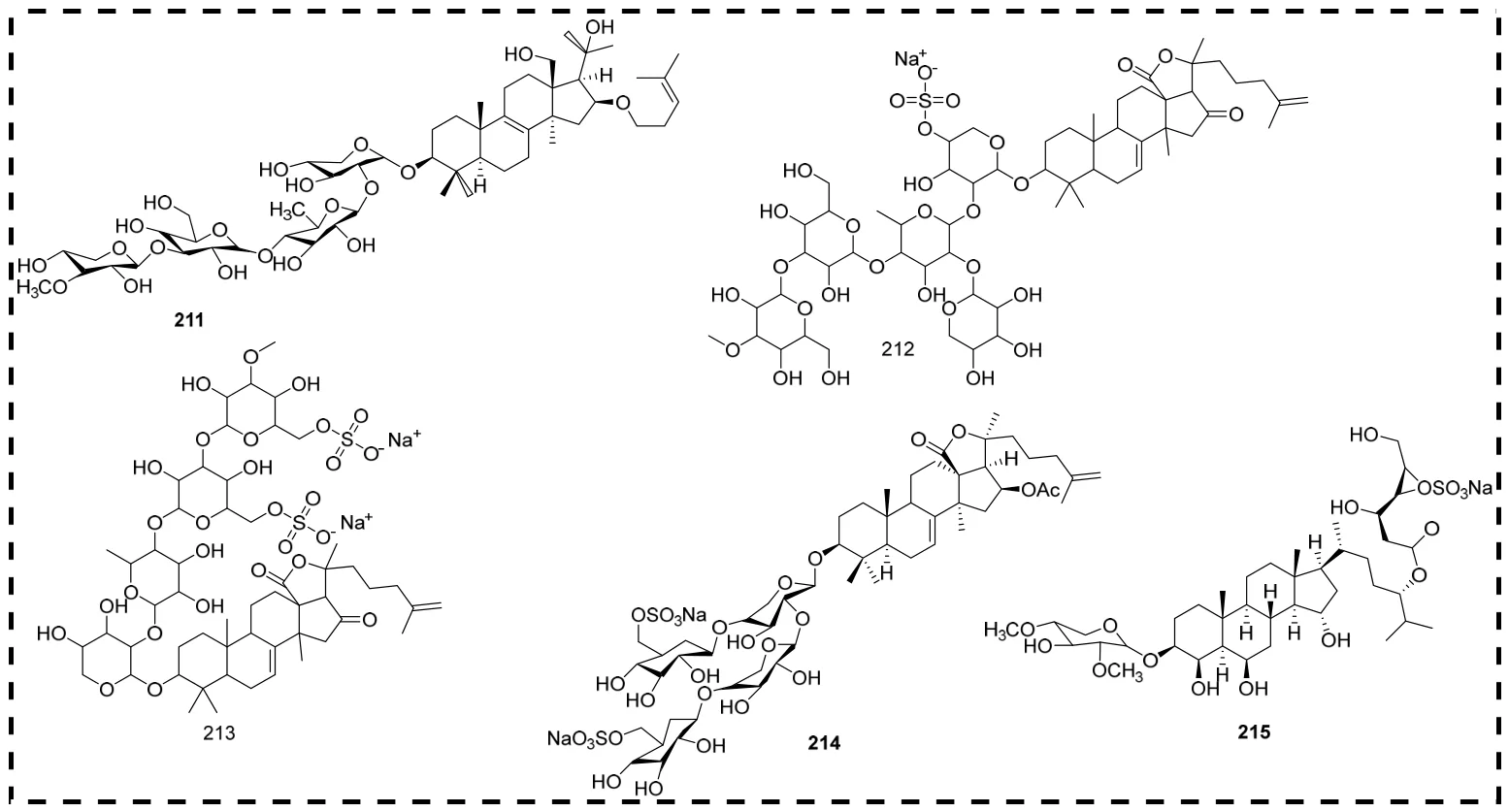
Marine-derived anti-cancer compounds (Part 6).

**Figure 20:**
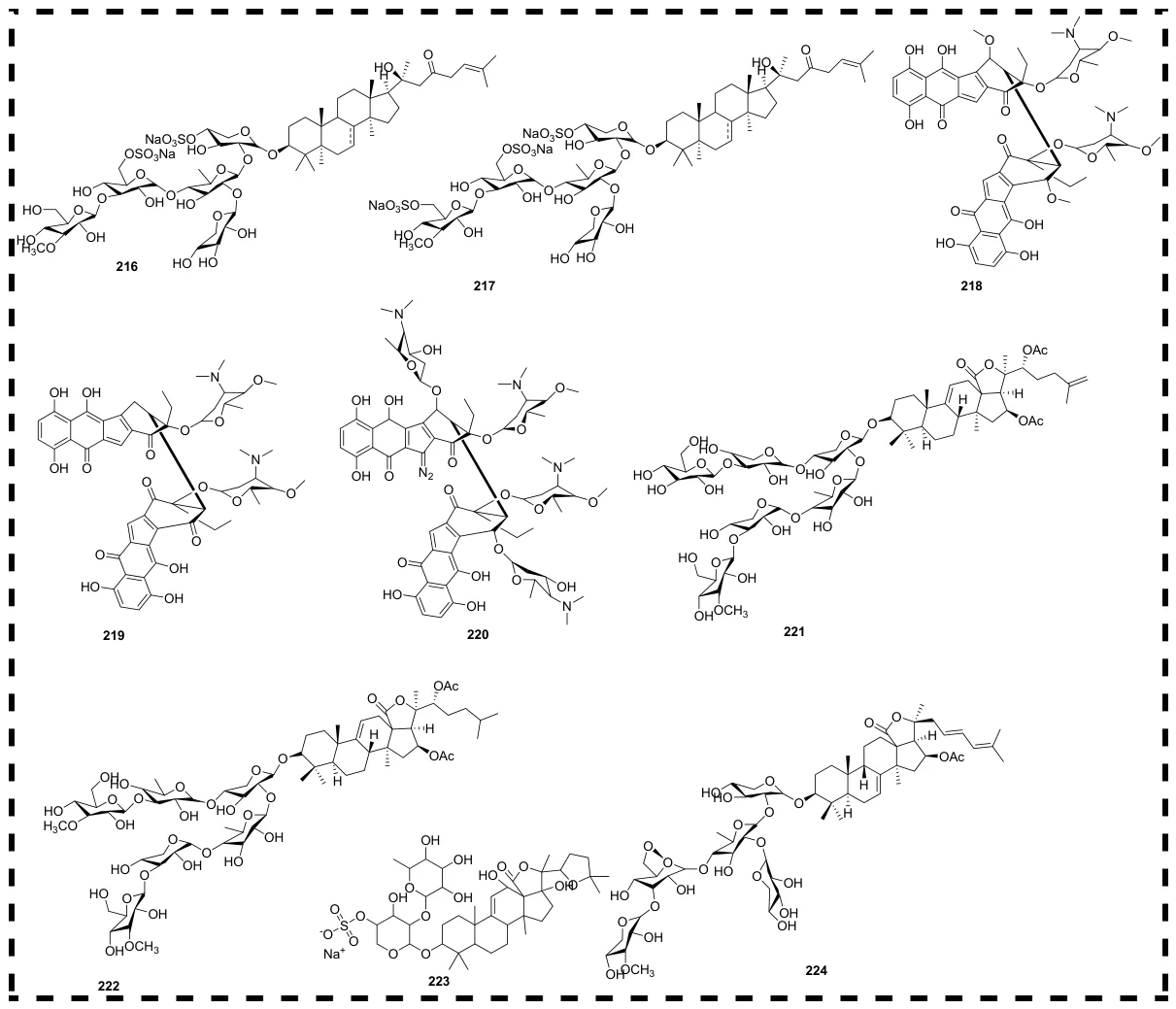
Marine-derived anti-cancer compounds (Part 7).

**Scheme 1:**
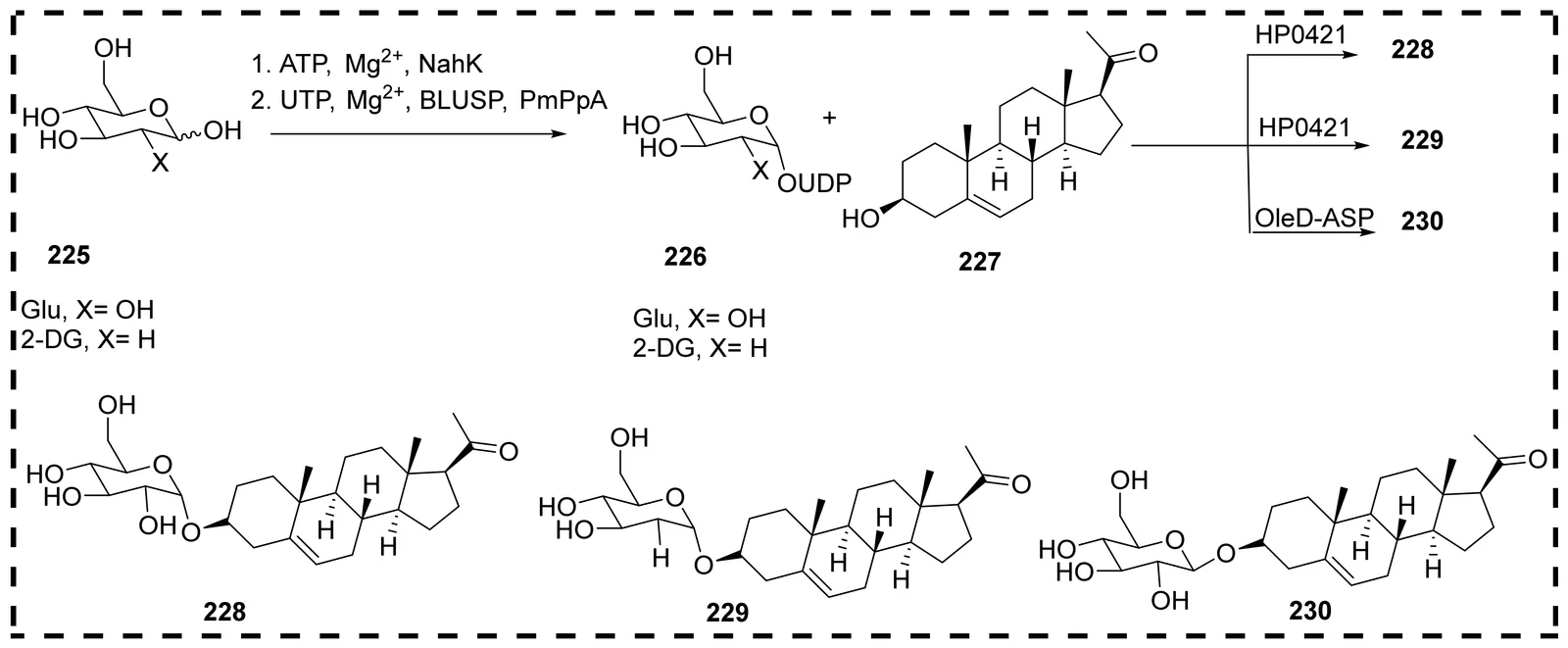
A multienzyme reaction catalyzed formation of Pregnenolonyl-*α*-glucoside.

**Scheme 2:**
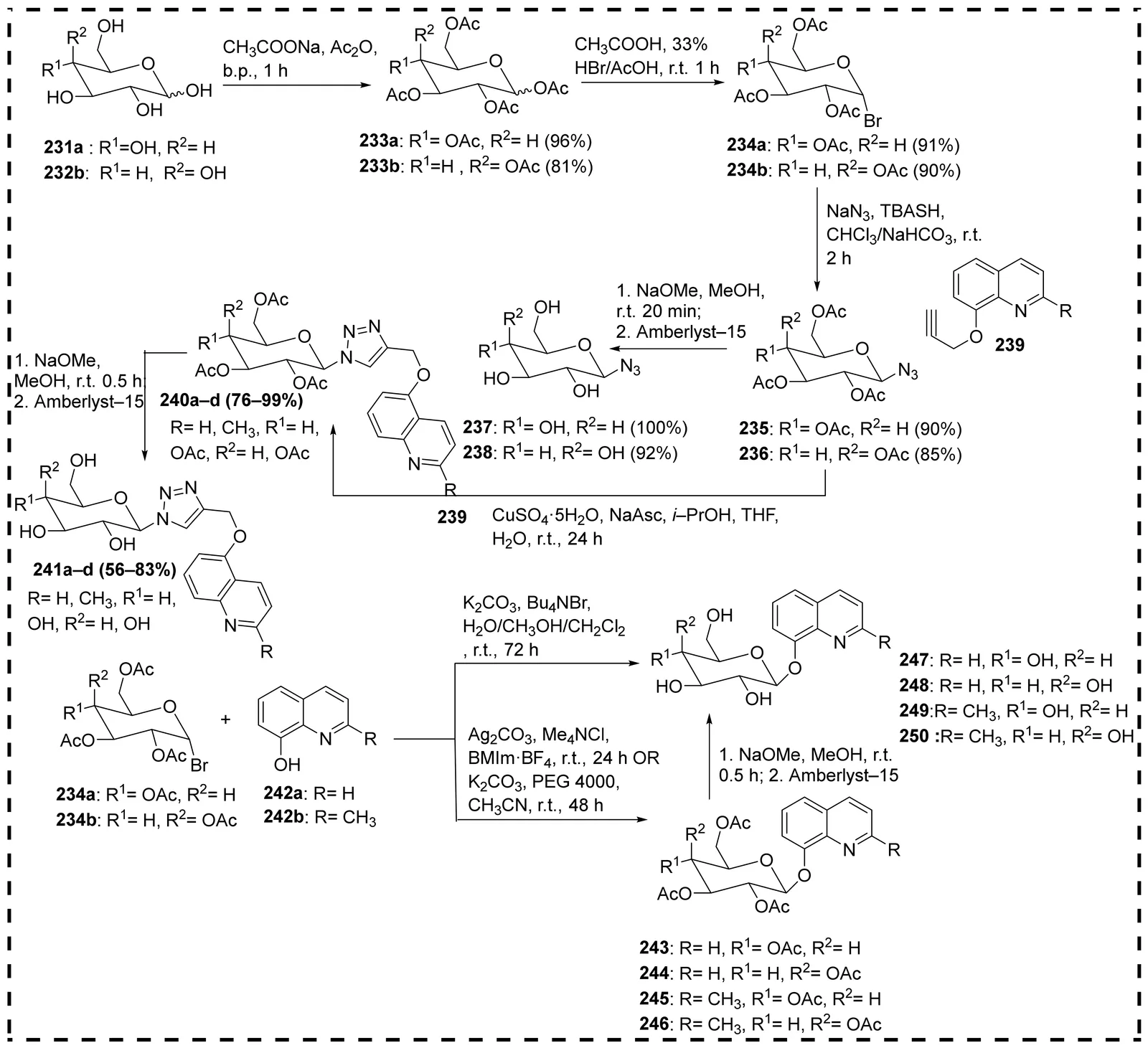
Synthesis of various 8-hydroquinone (8-HQ) containing glycoconjugates.

**Scheme 3:**
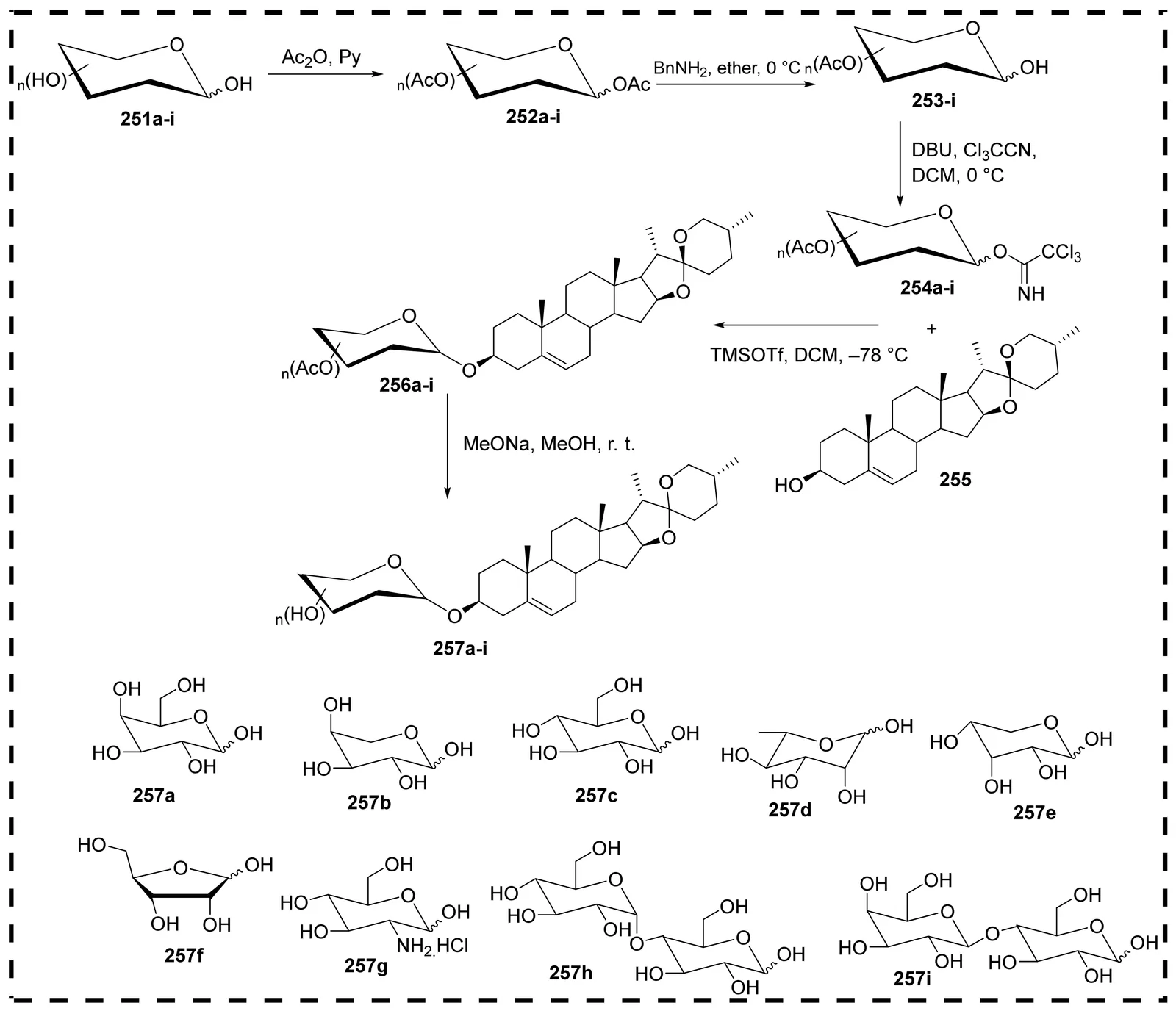
Synthesis of Diosgenin-derived glycosylated compounds.

**Scheme 4:**
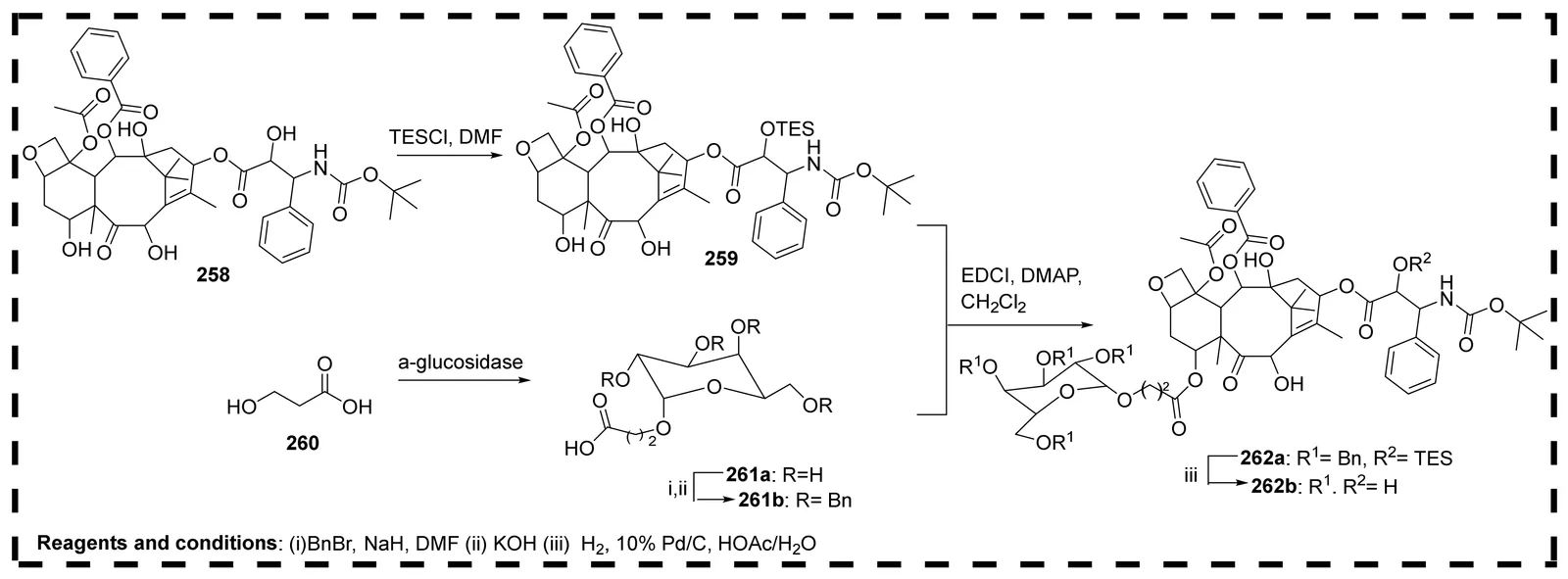
Formation of 7-propionyldocetaxel 3″-*O*-*α*-D-glucopyranoside *via* enzymatic pathway.

**Scheme 5:**
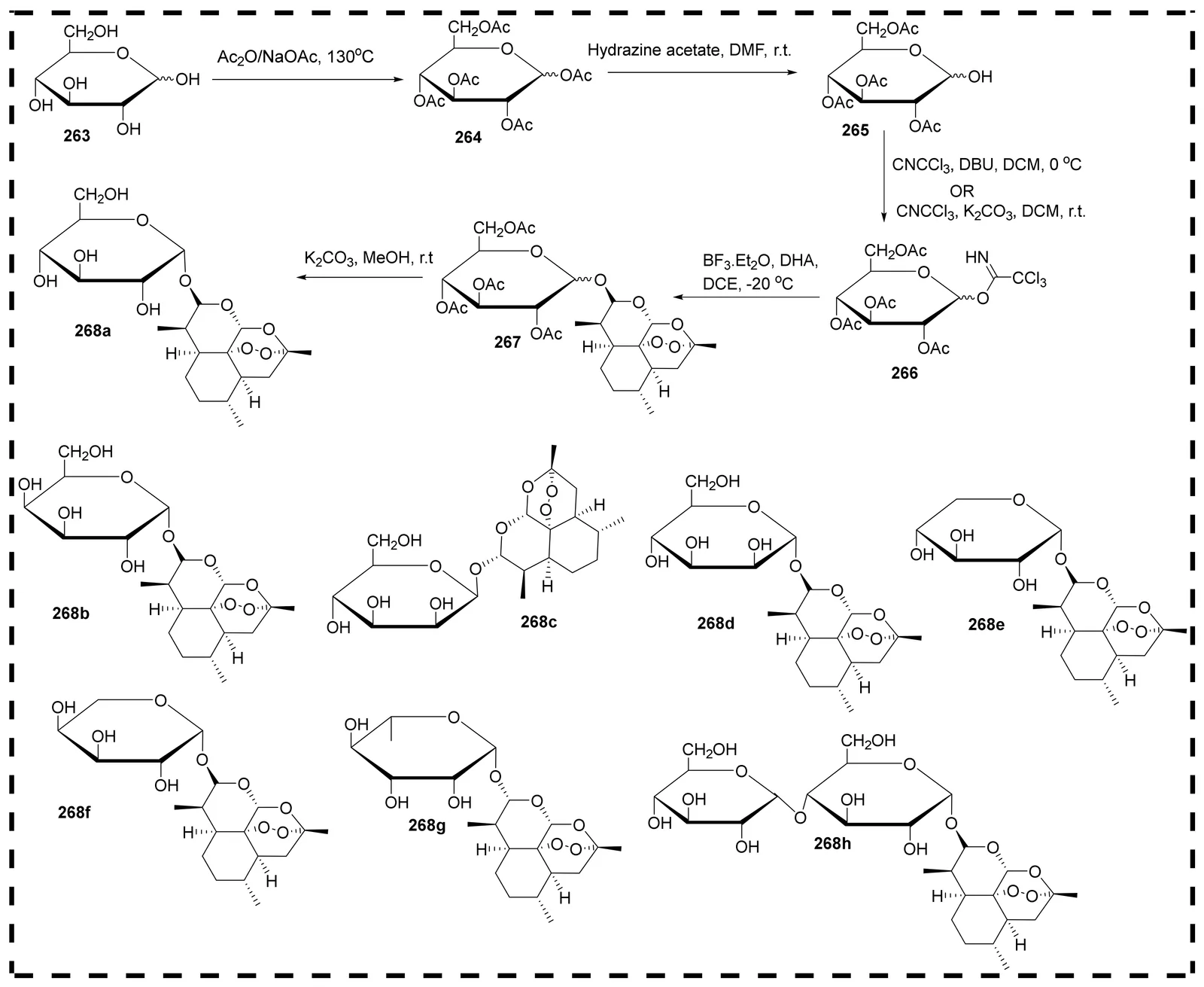
Synthesis of dihydroartemisinin-*O*-glycosides.

**Scheme 6:**
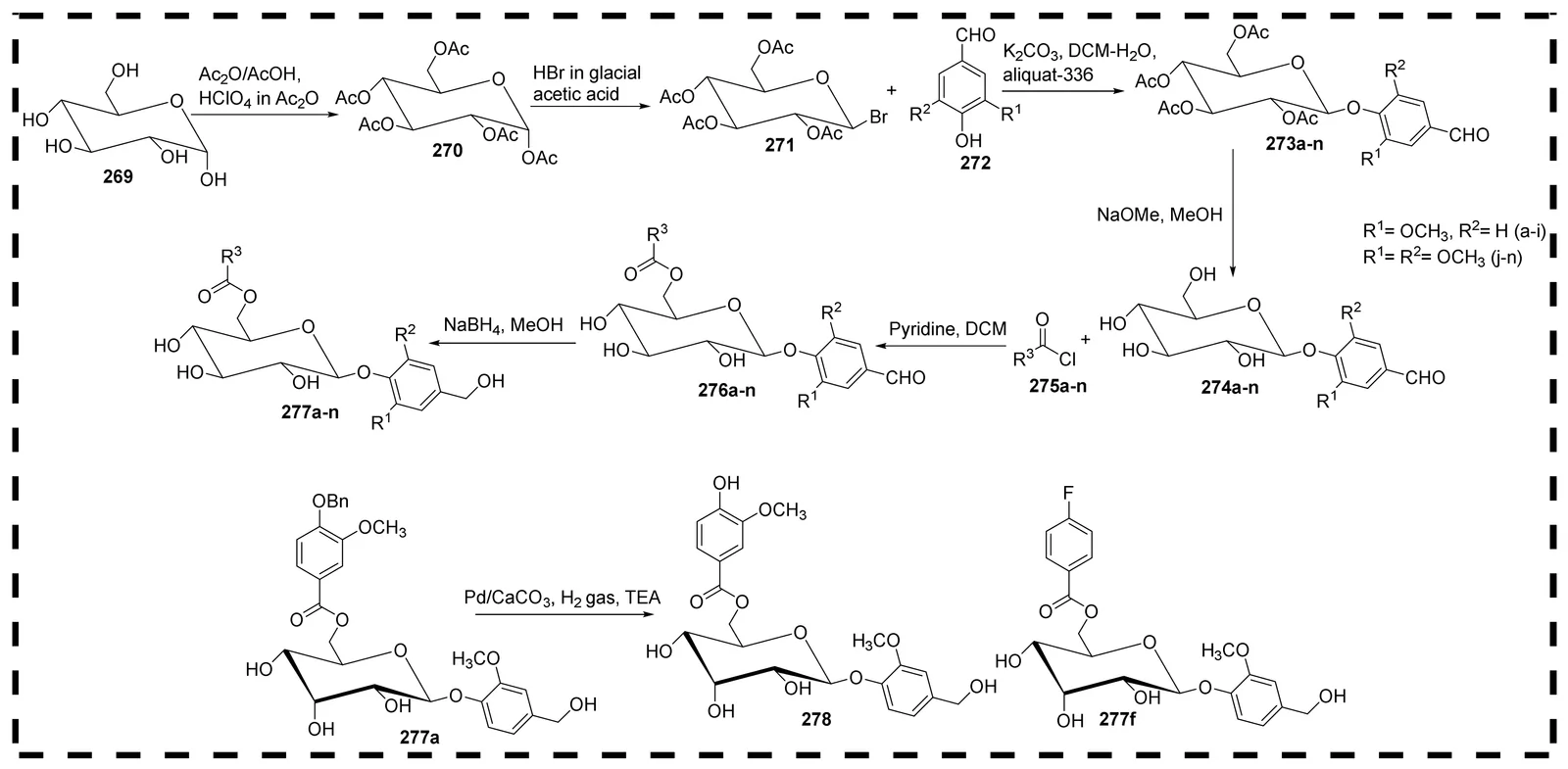
Formation of phenolic glycoside esters via Koenigs-Knorr reaction.

**Scheme 7:**
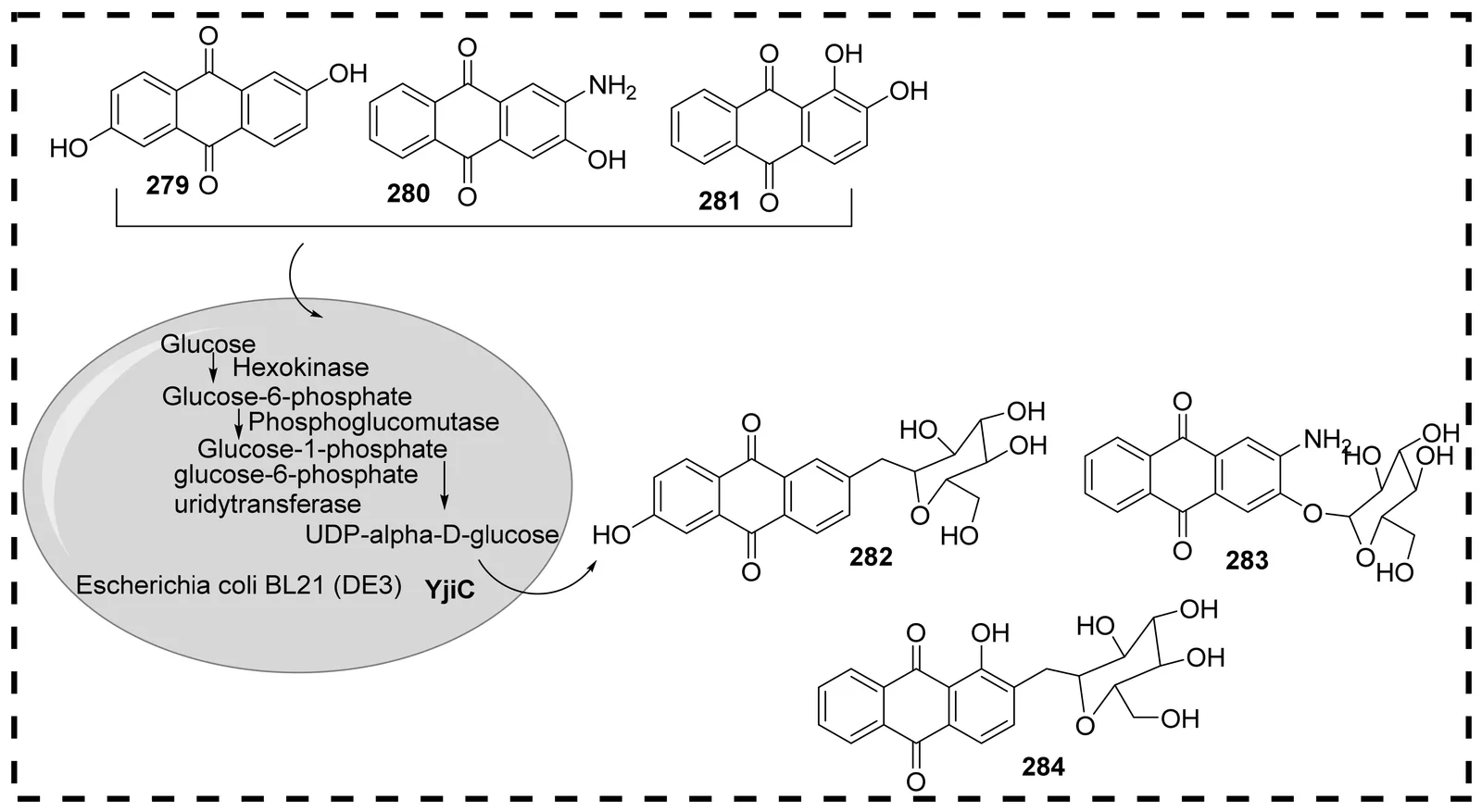
*O*-Glycoside derivatives of anthraquinones via multi-enzymatic synthetic route.

**Scheme 8:**
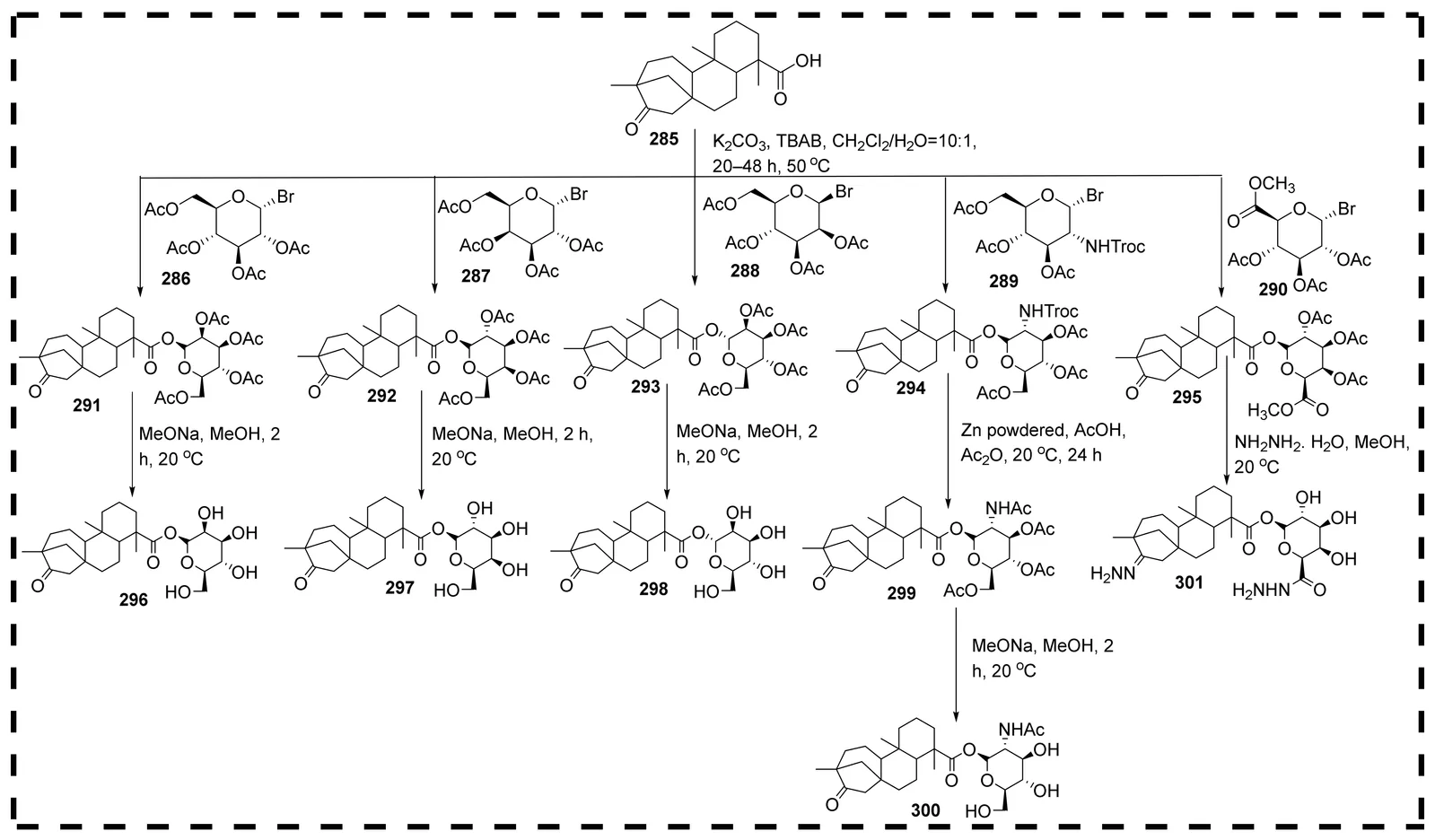
Synthesis of a range of diterpenoid isosteviol glycosides.

**Scheme 9:**
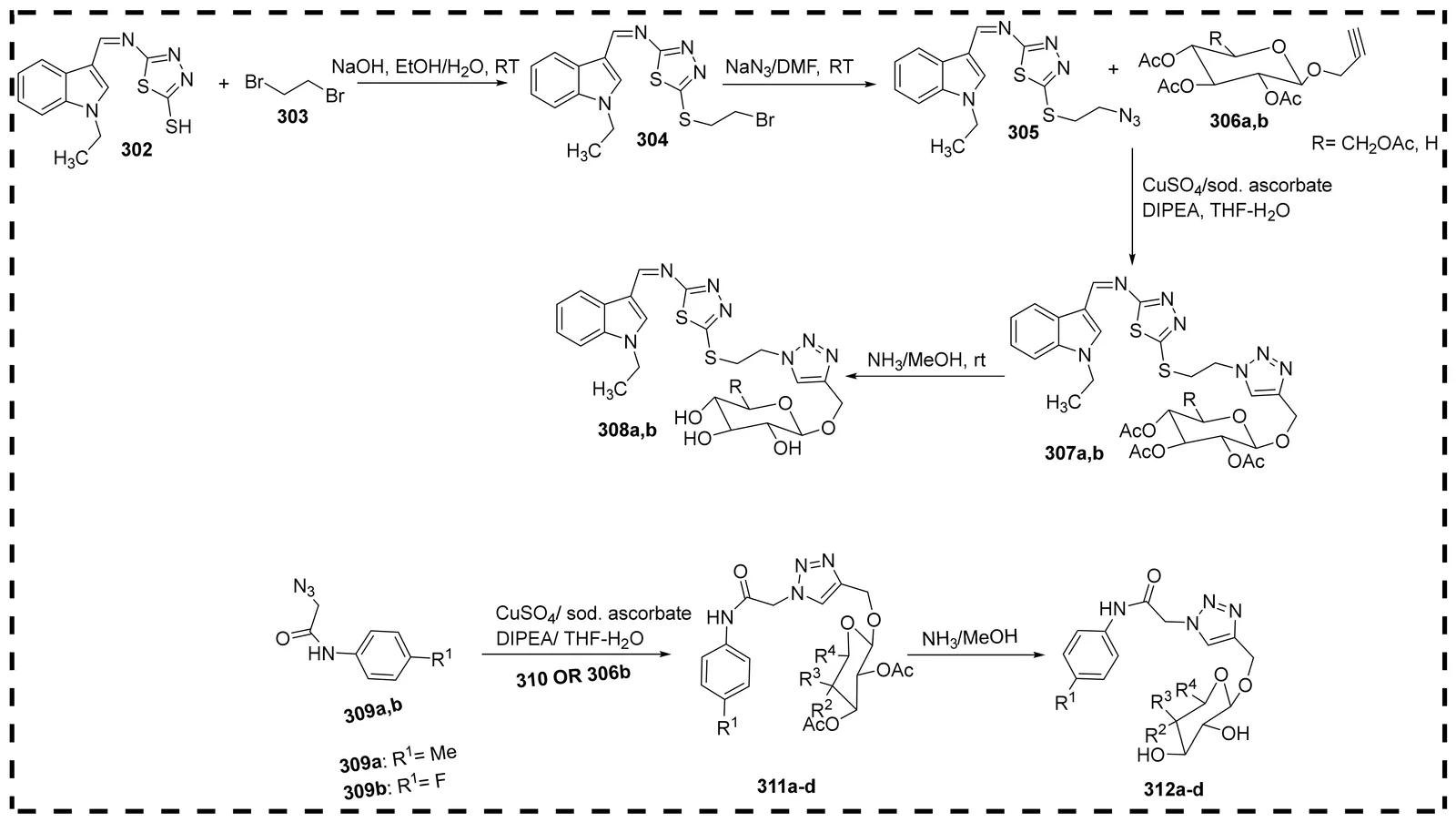
Formation of new 1,3,4-thiadiazole thioglycosides using 1,3-dipolar cycloaddition.

**Scheme 10:**
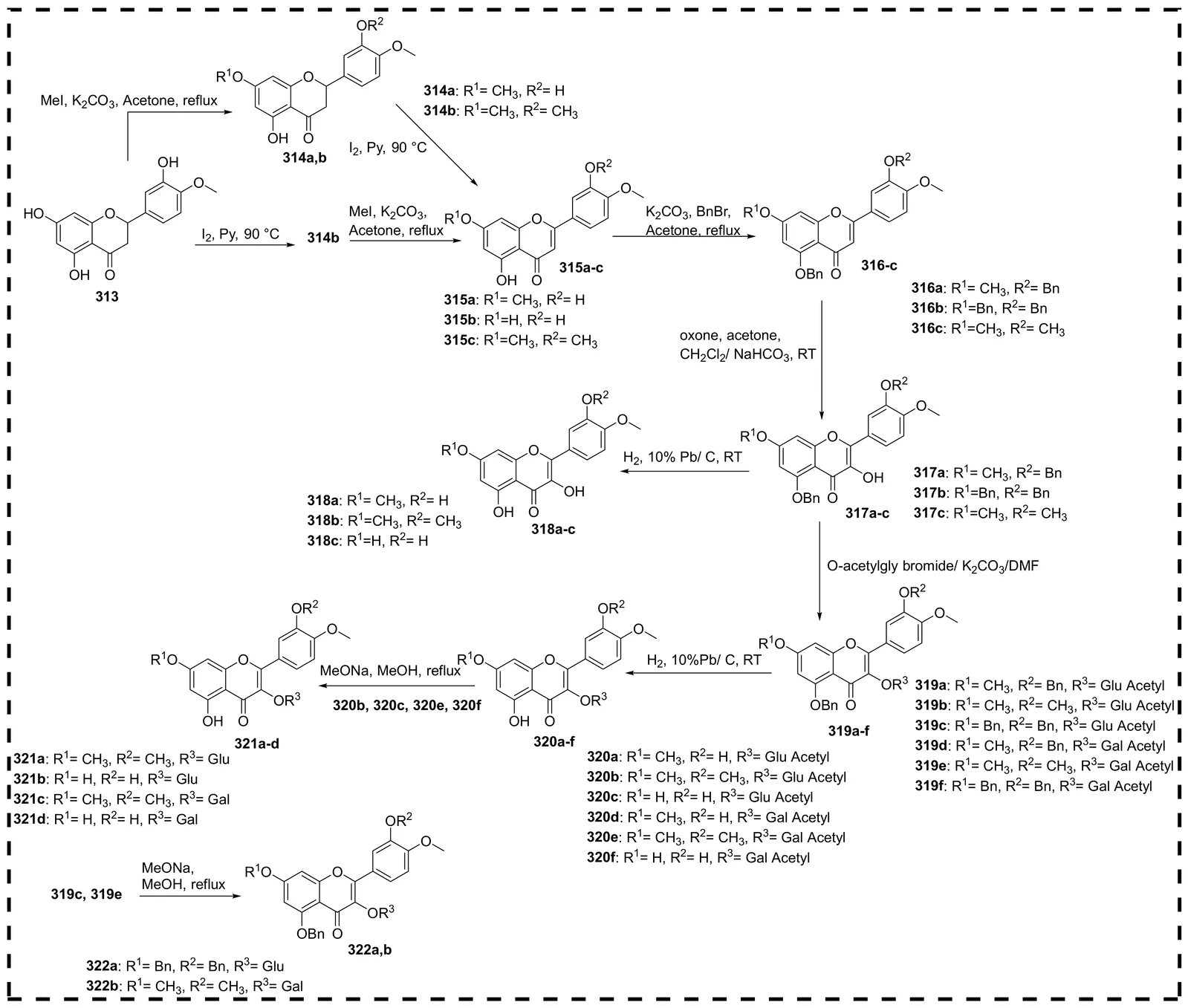
Synthesis of hesperetin-derived flavonoid glycosides.

**Scheme 11:**
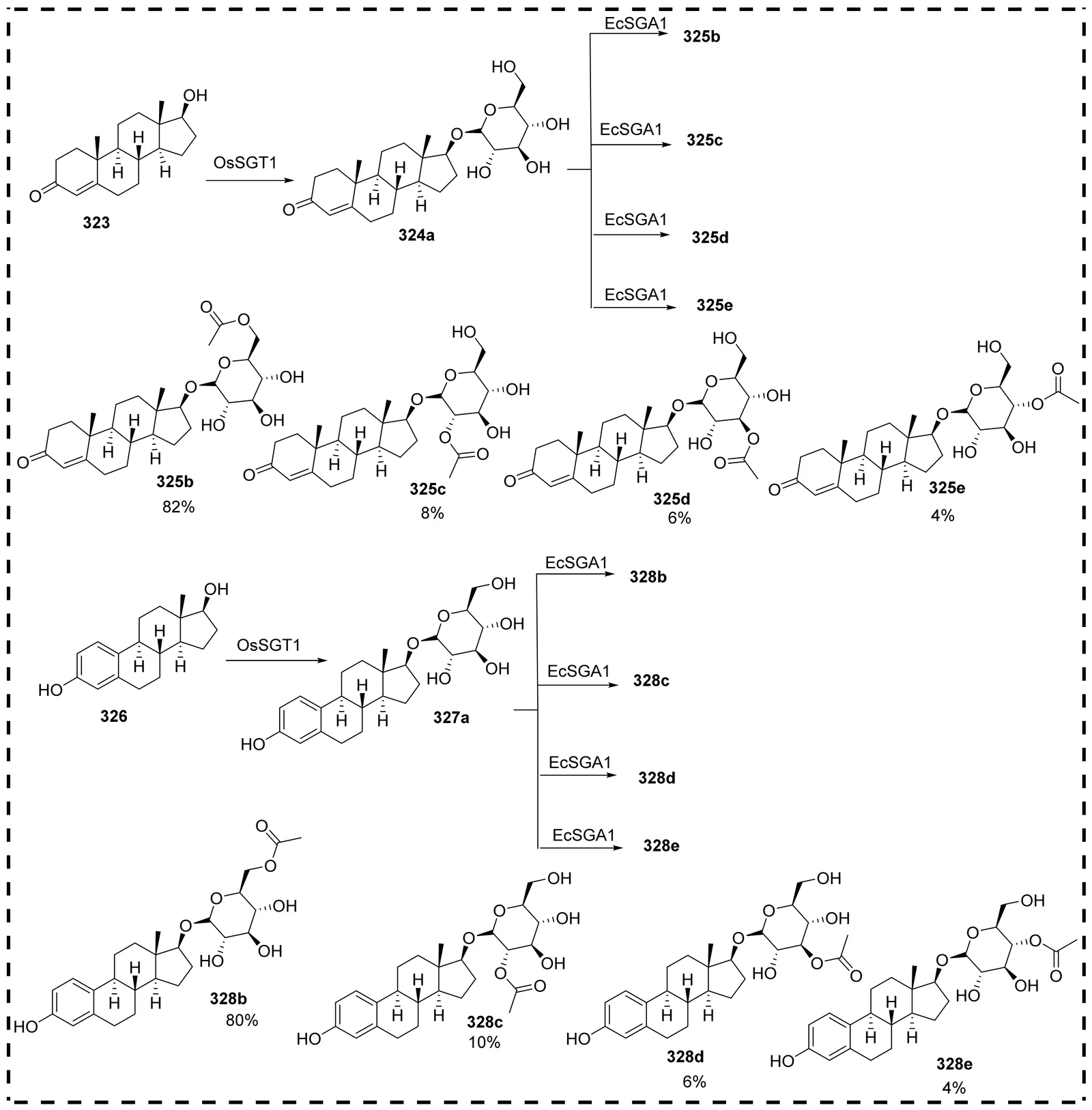
Formation of some biologically active acylated steroidal glycosides.

**Scheme 12:**
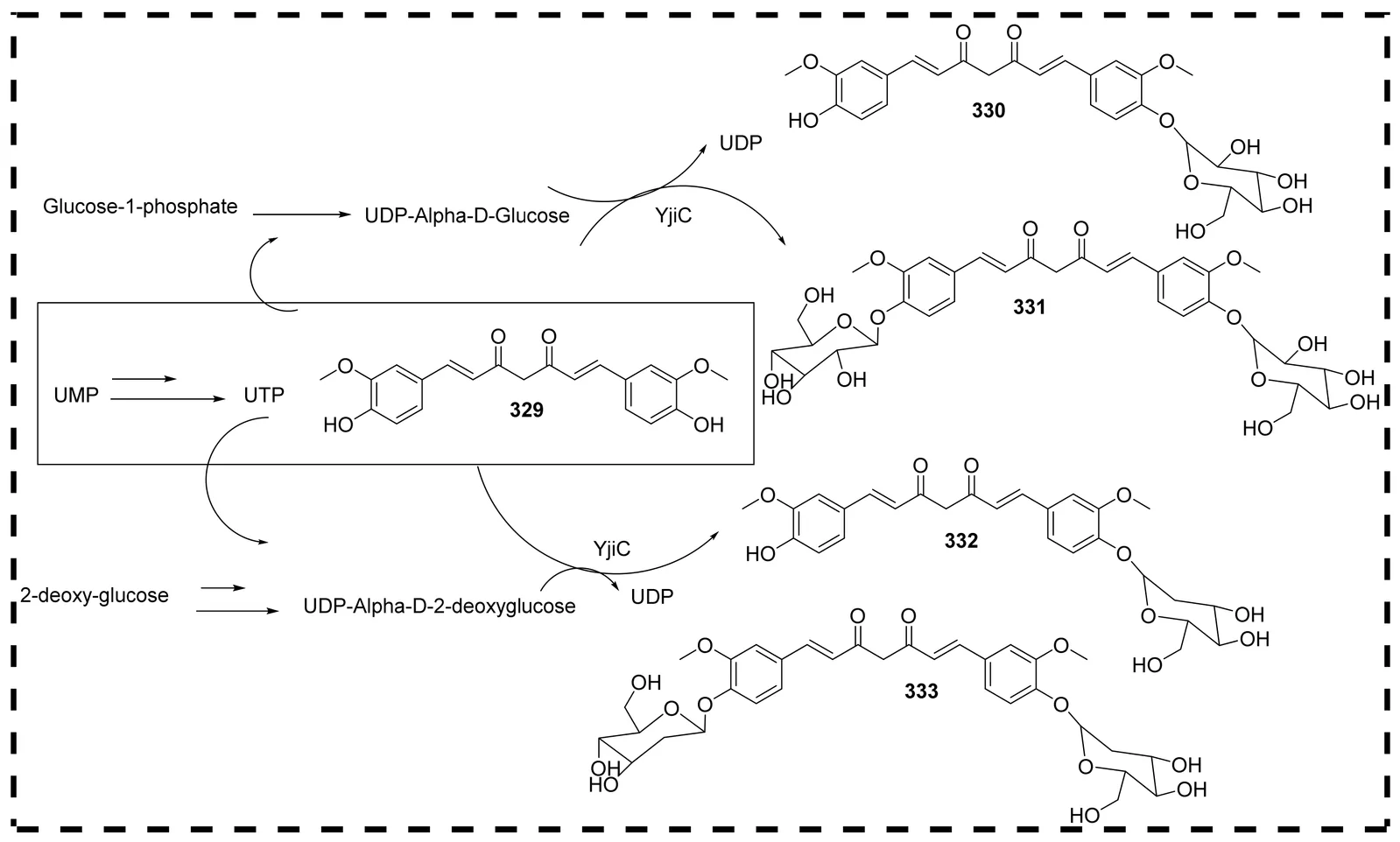
A facile Enzymatic formation of glycosylated curcumin derivatives.

**Scheme 13:**
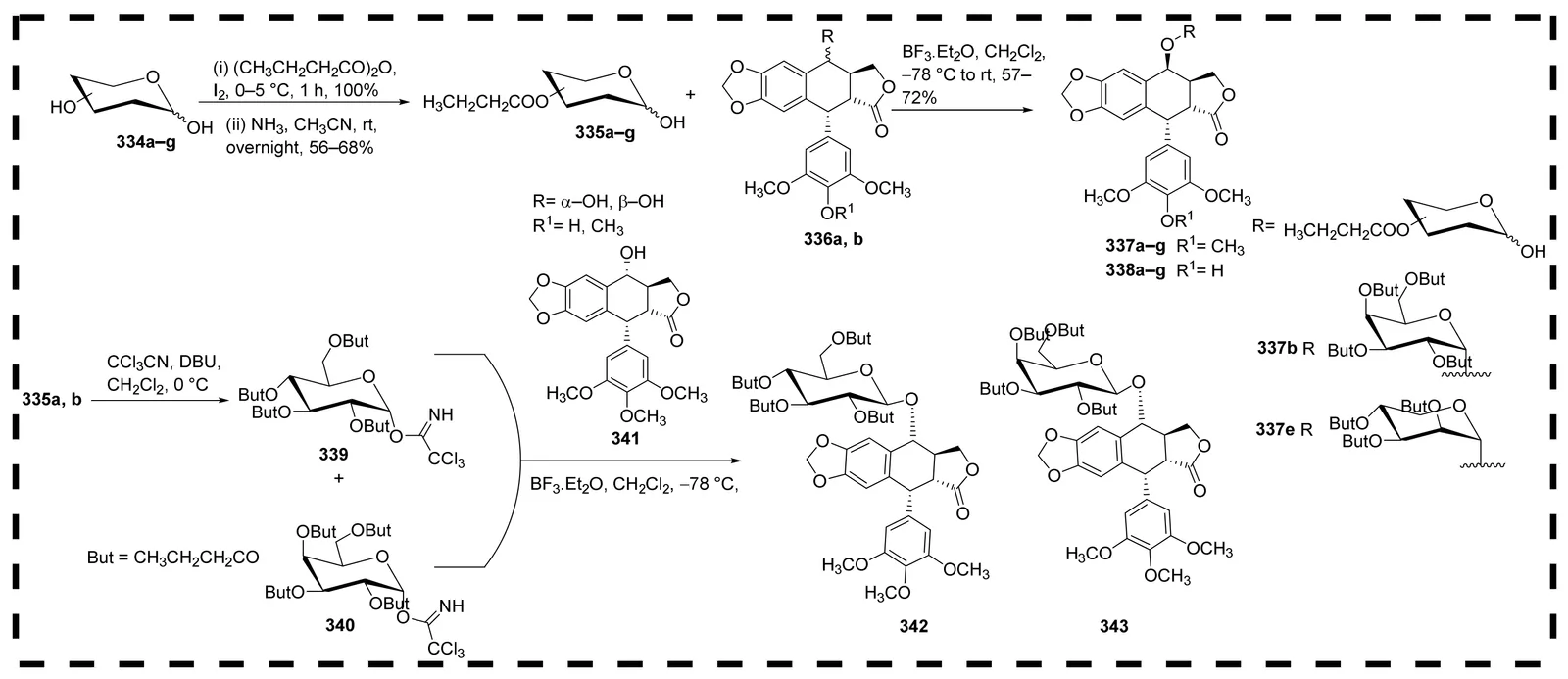
Synthesis of Podophyllotoxin glycosides.

**Scheme 14:**
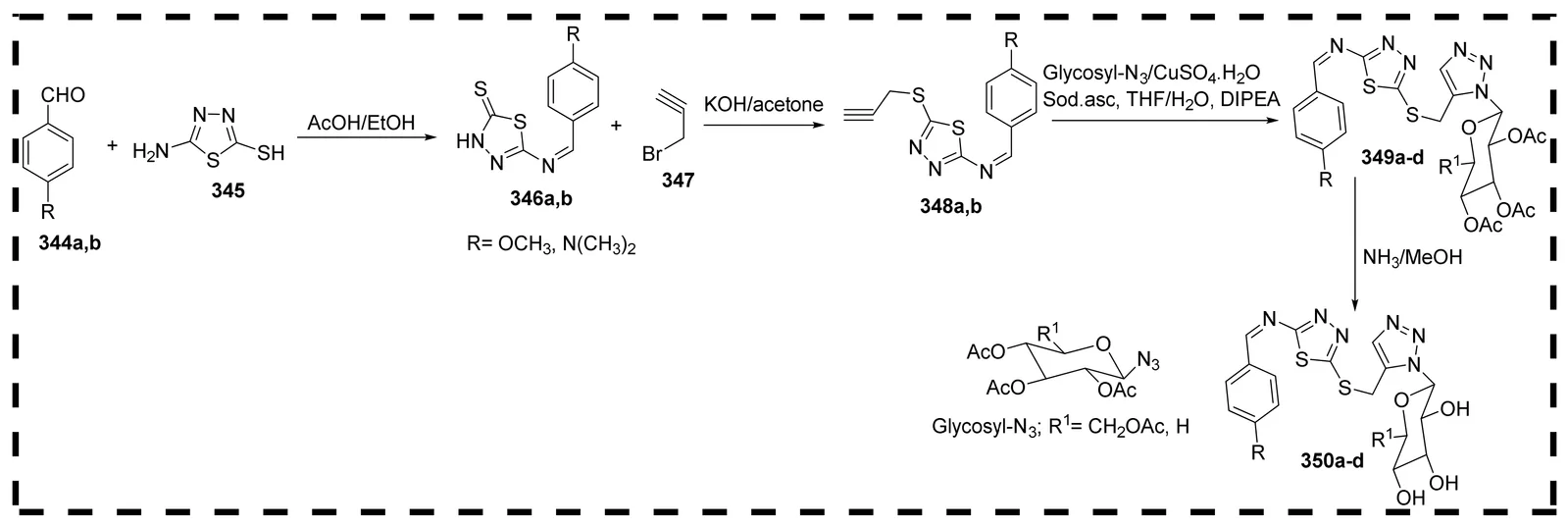
Formation of 1,3,4-thiadiazole and 1,2,3-triazole hybrid glycosides.

**Scheme 15:**
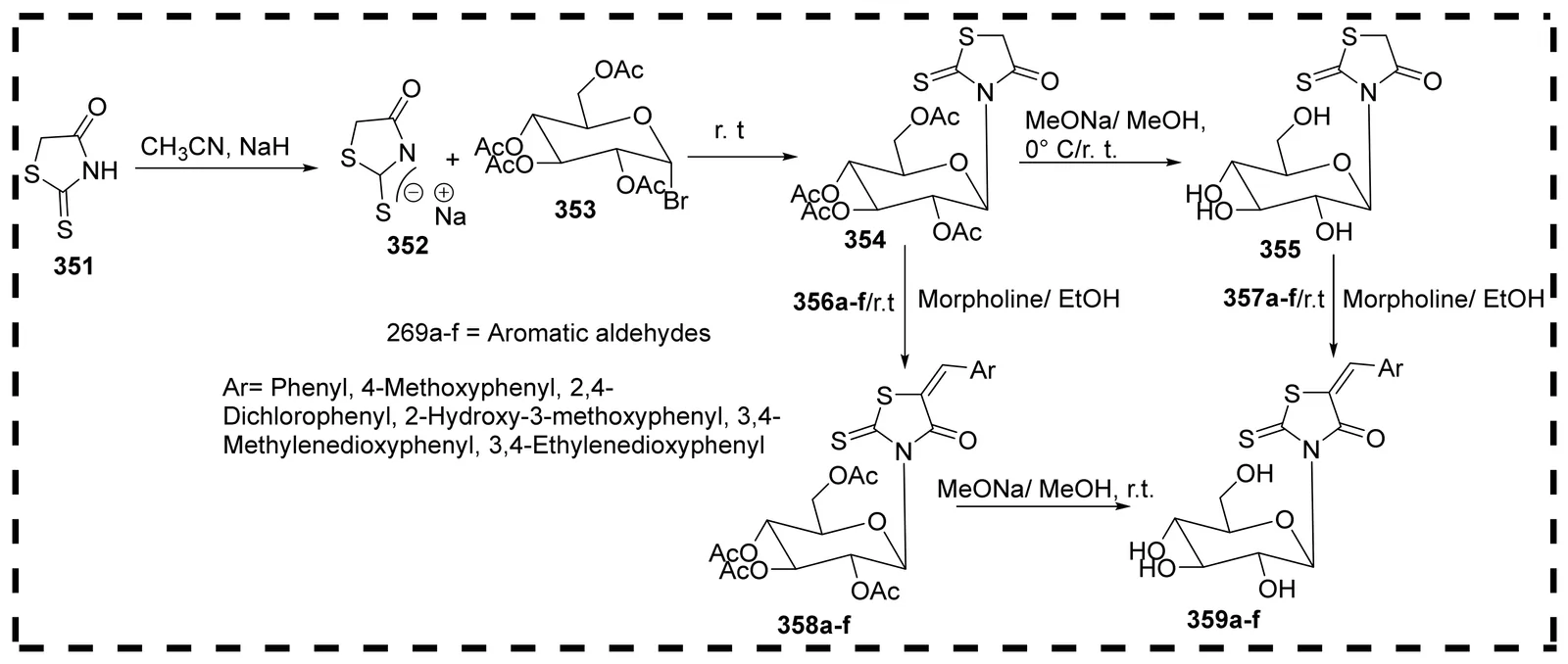
Formation of N-Rhodanine Glycoside Derivatives.

**Scheme 16:**
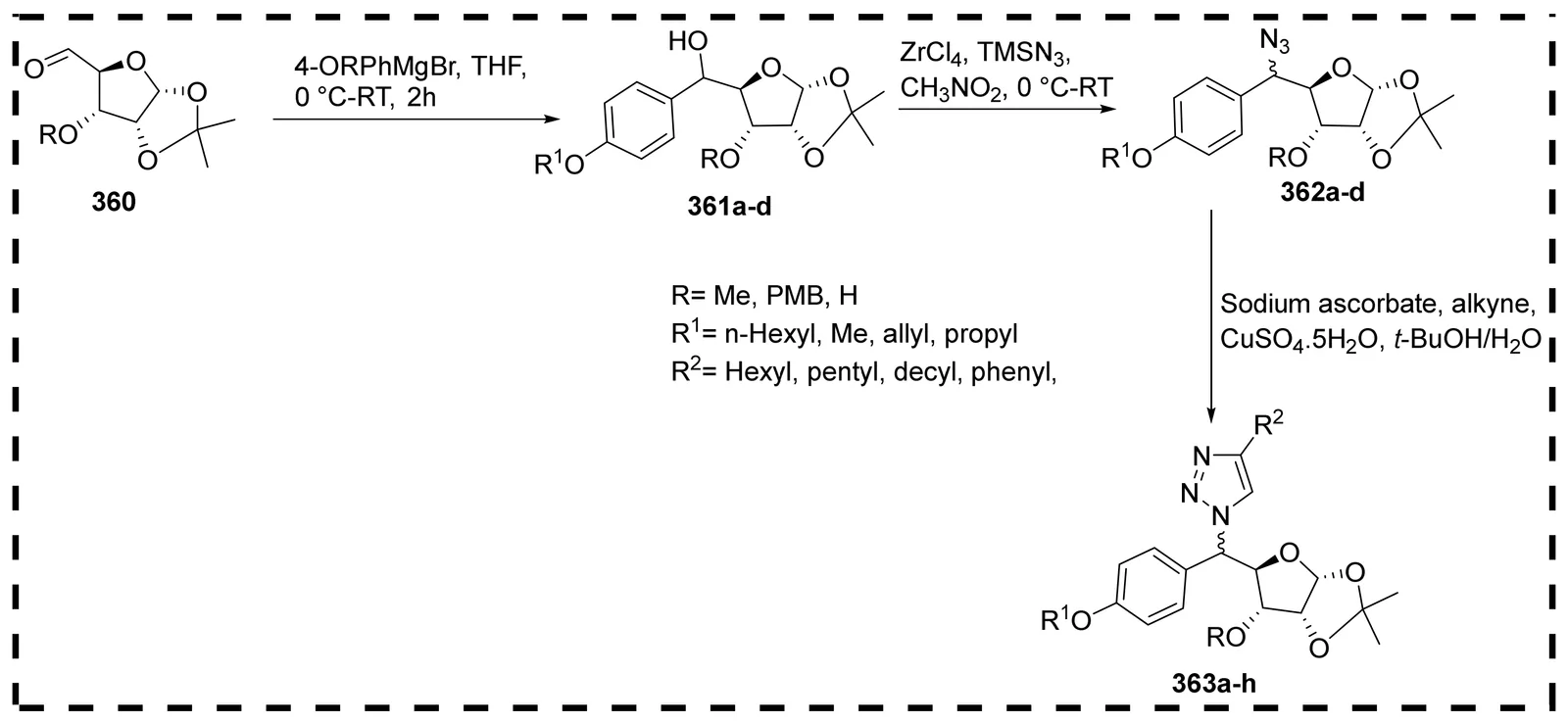
Synthesis of N-glycosides containing a triazole ring.

**Scheme 17:**
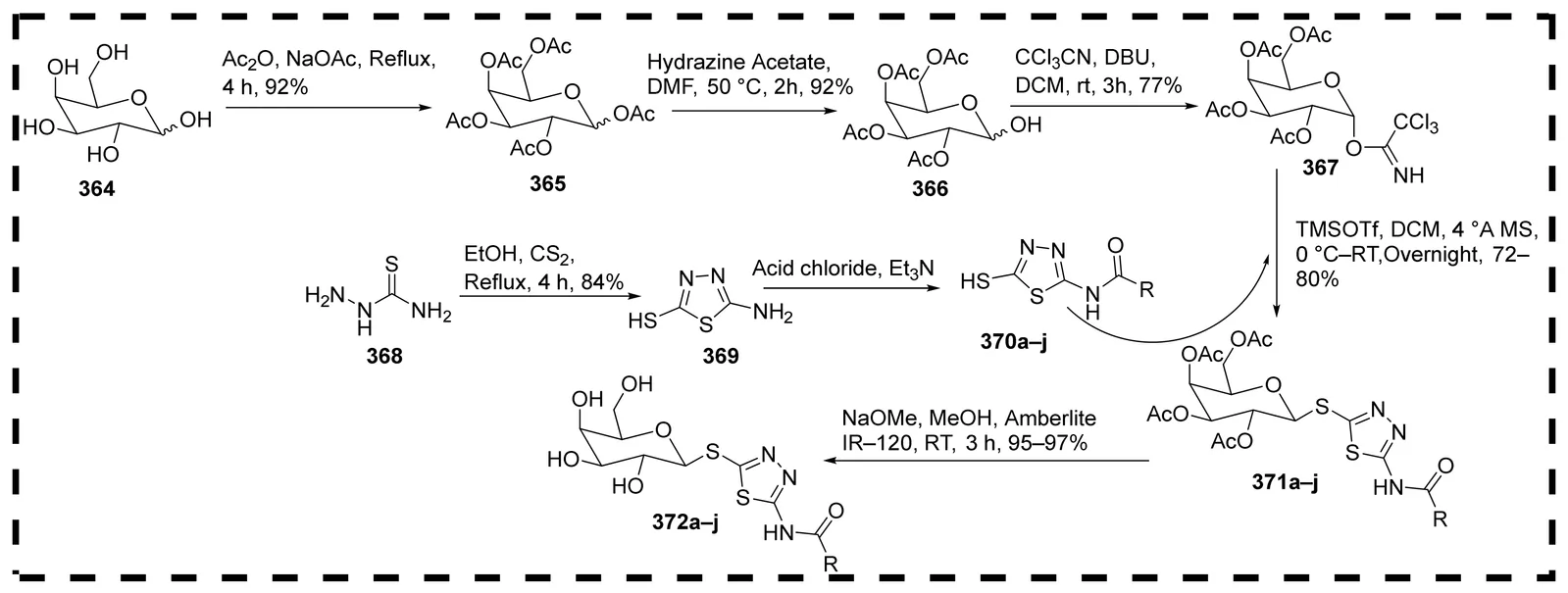
Synthesis of thiadiazole-based thioglycoside.

**Scheme 18:**
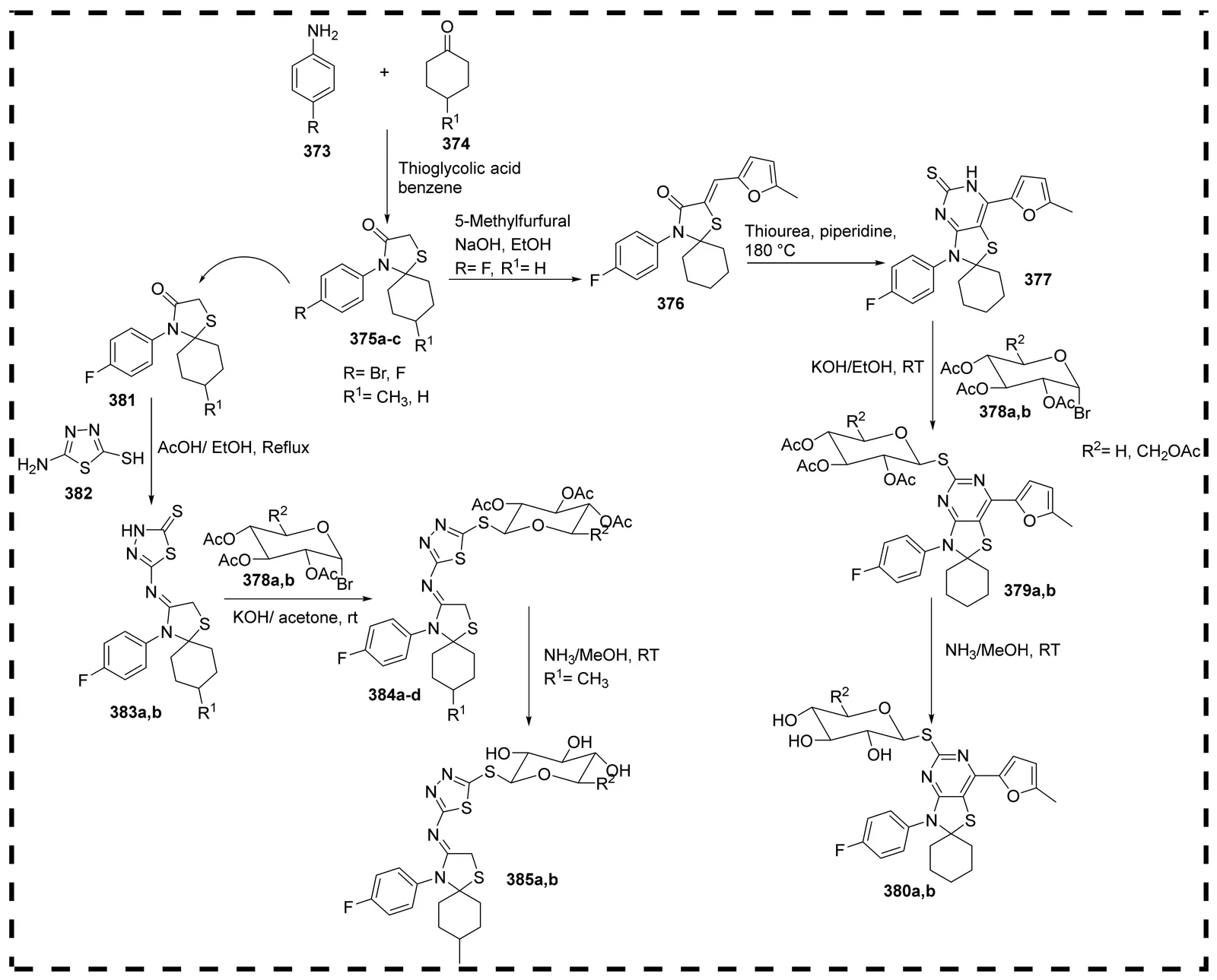
Formation of thioglycoside derivatives of (1,3,4thiadiazolyl)thiaazaspiro[4.5]decane.

**Scheme 19:**
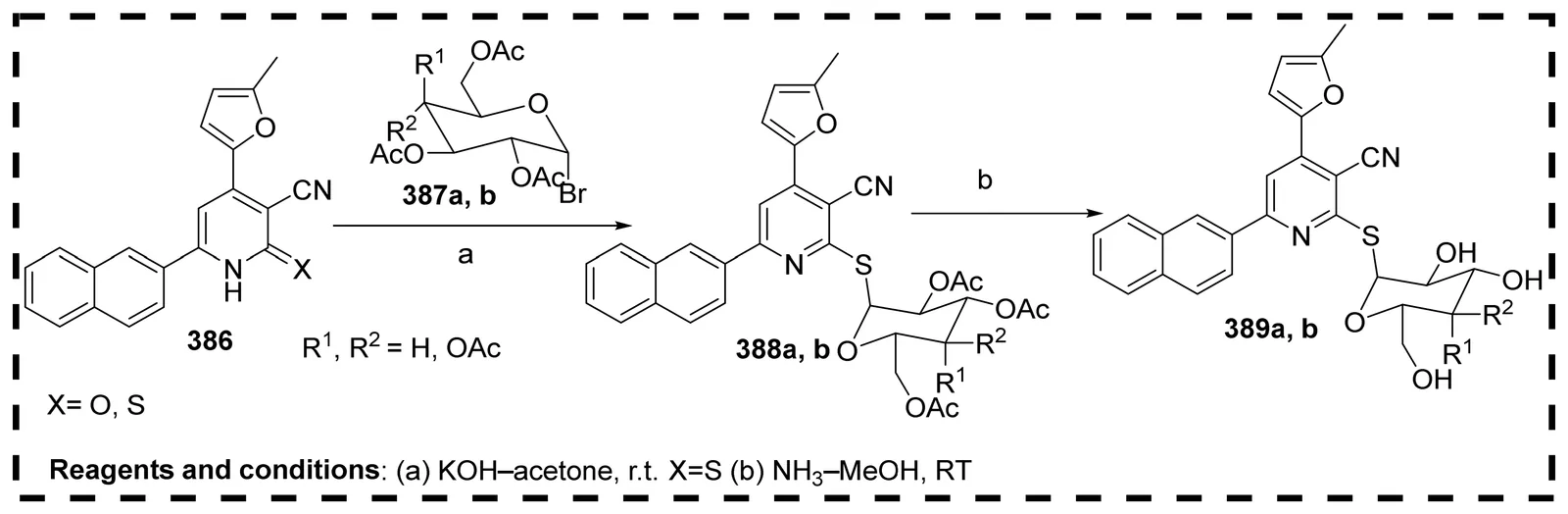
Synthesis of pyridine-containing thioglycosides.

**Scheme 20:**
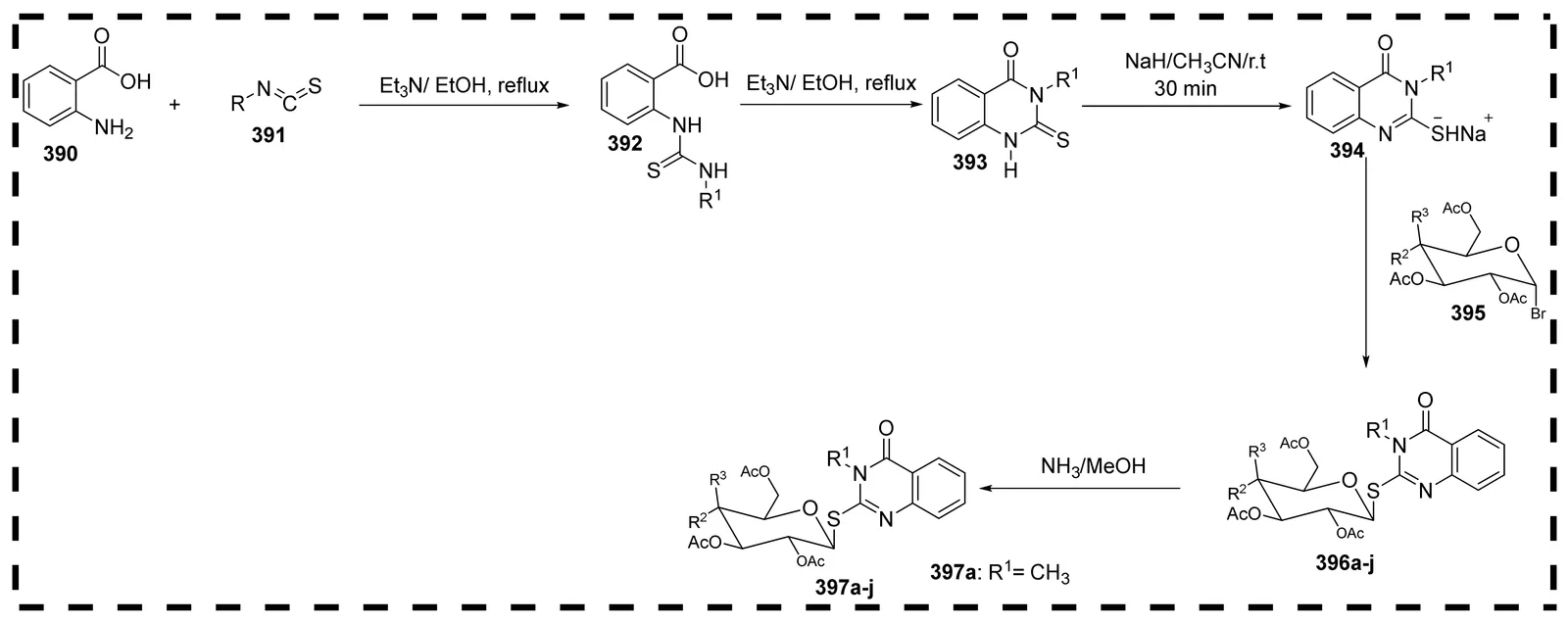
Formation of a series of quinazoline-based S-glycosides.

**Scheme 21:**
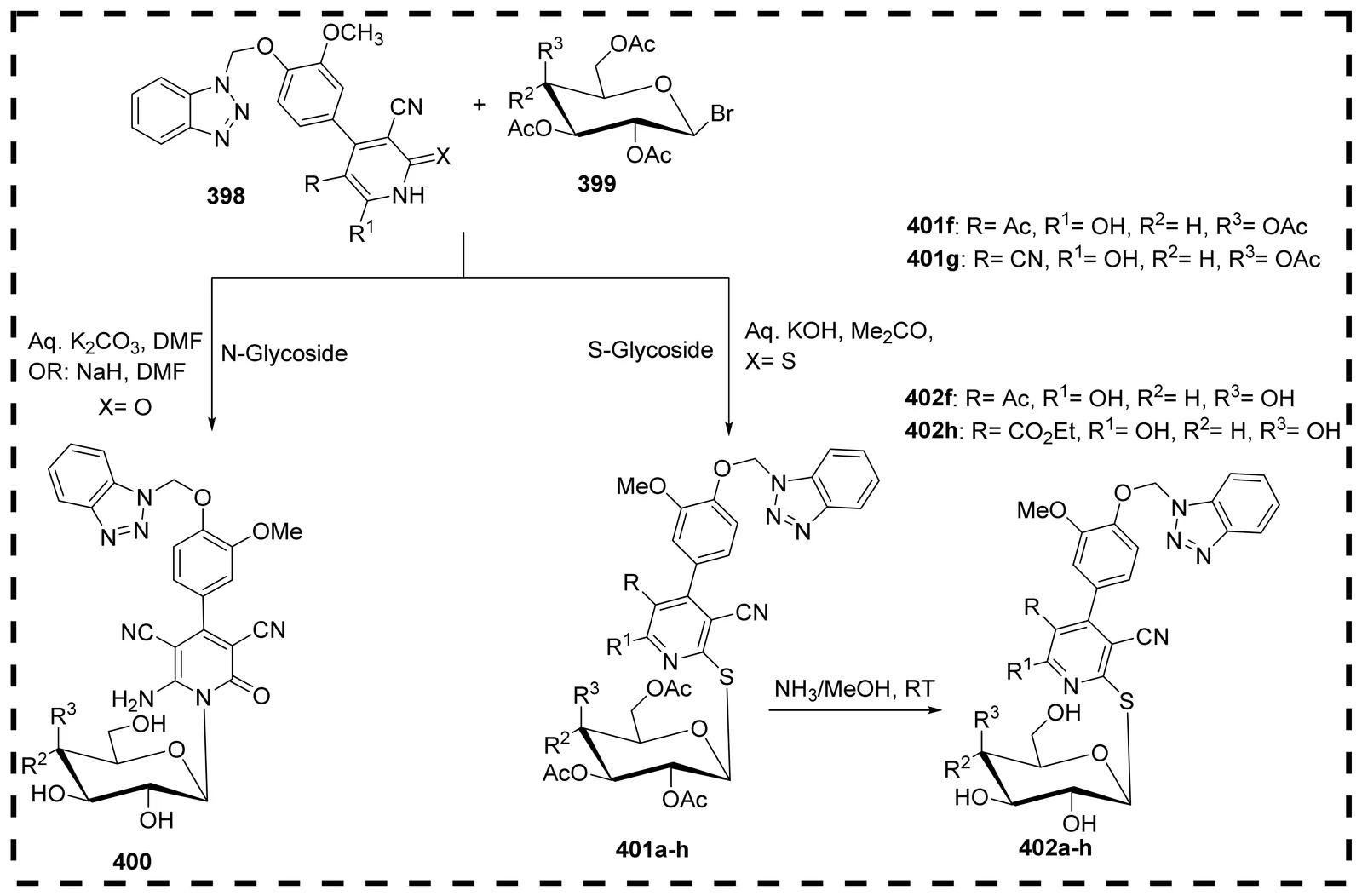
Synthesis of N- and S-glycosides of the polyfunctionalized pyridine-biphenyl system.

**Scheme 22:**
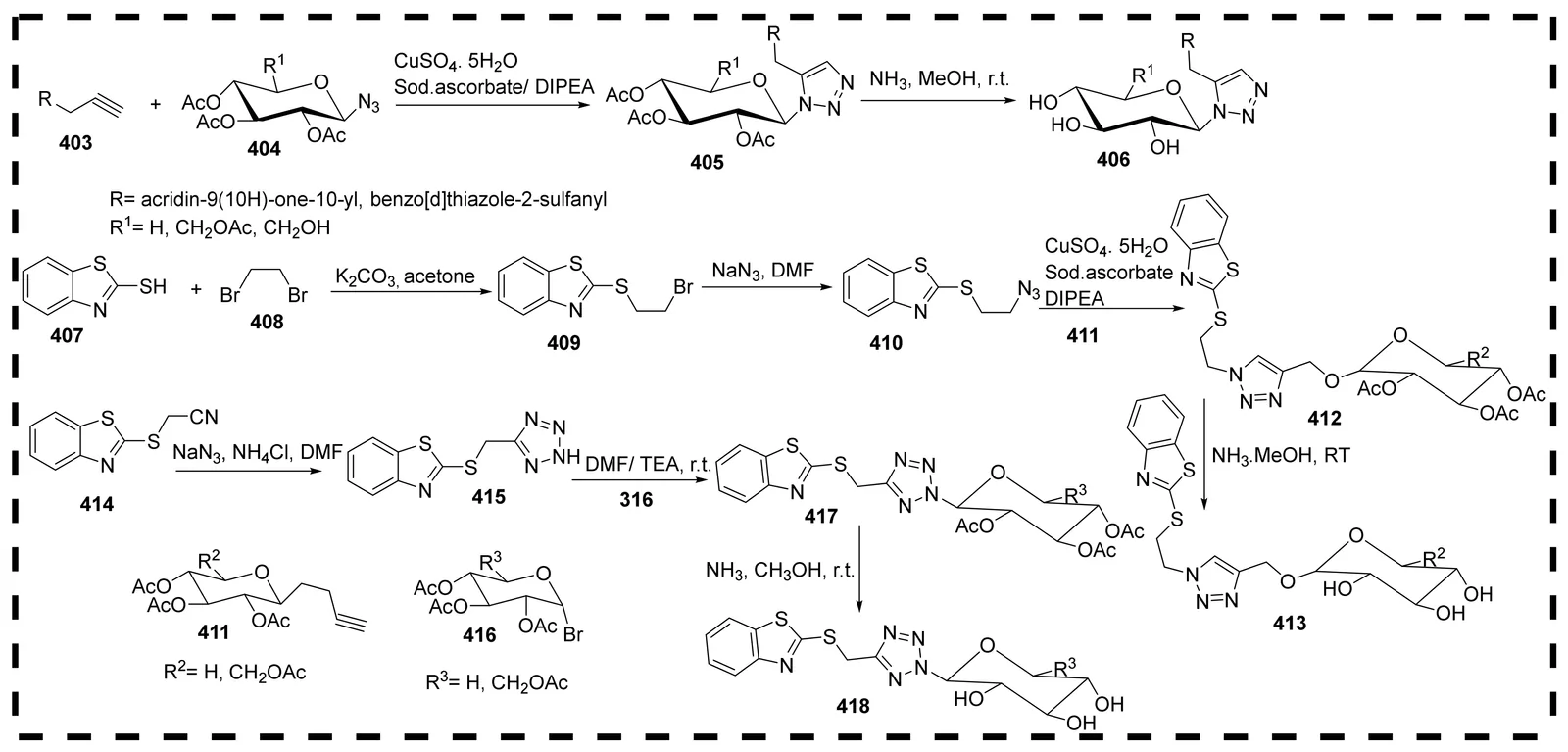
Synthesis of 1,2,3-triazole glycosides substituted with acridine and benzothiazole.

**Scheme 23:**
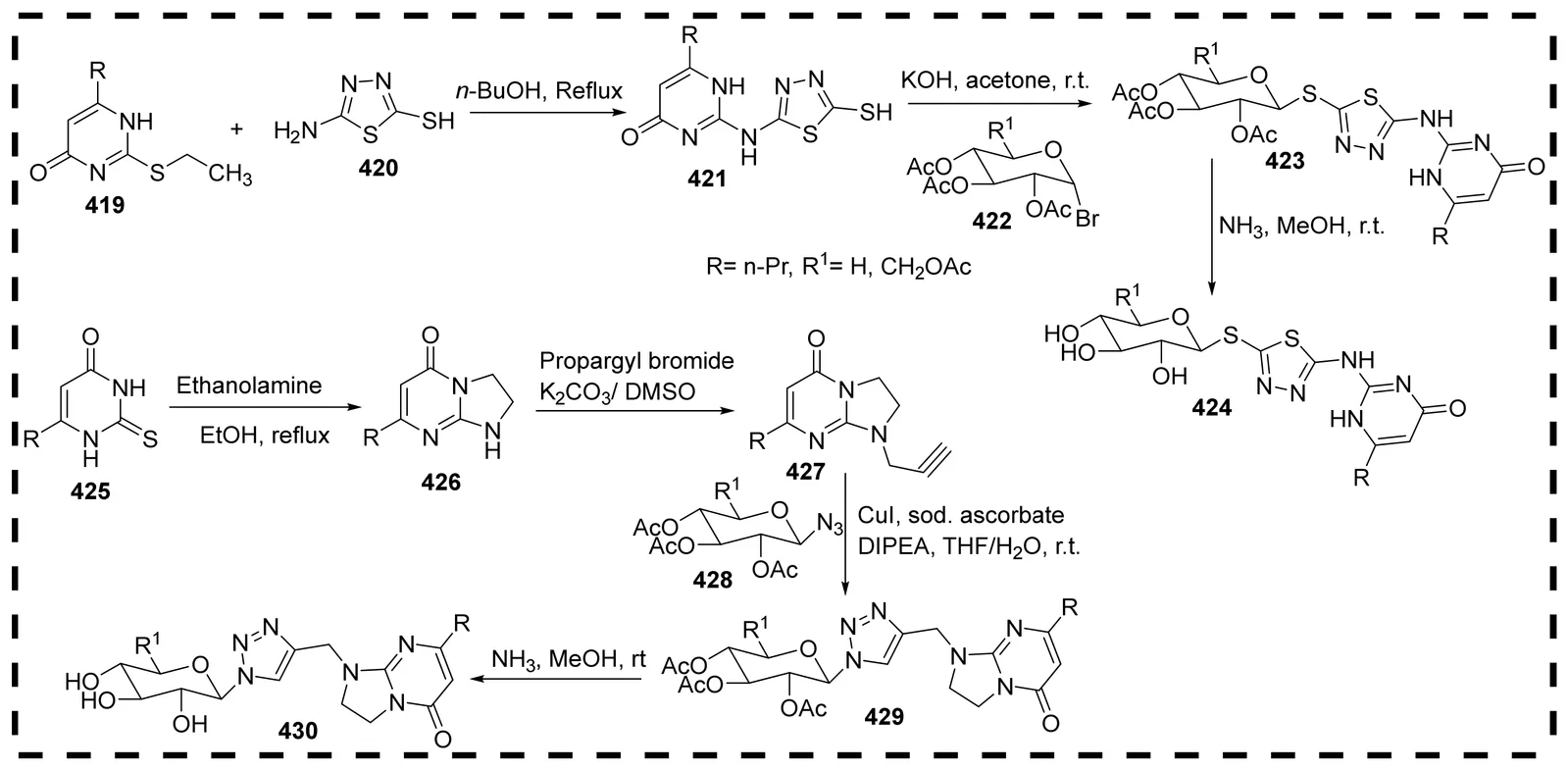
Formation of 1,3,4-thiadiazole thioglycosides containing substituted pyrimidines.

**Scheme 24:**
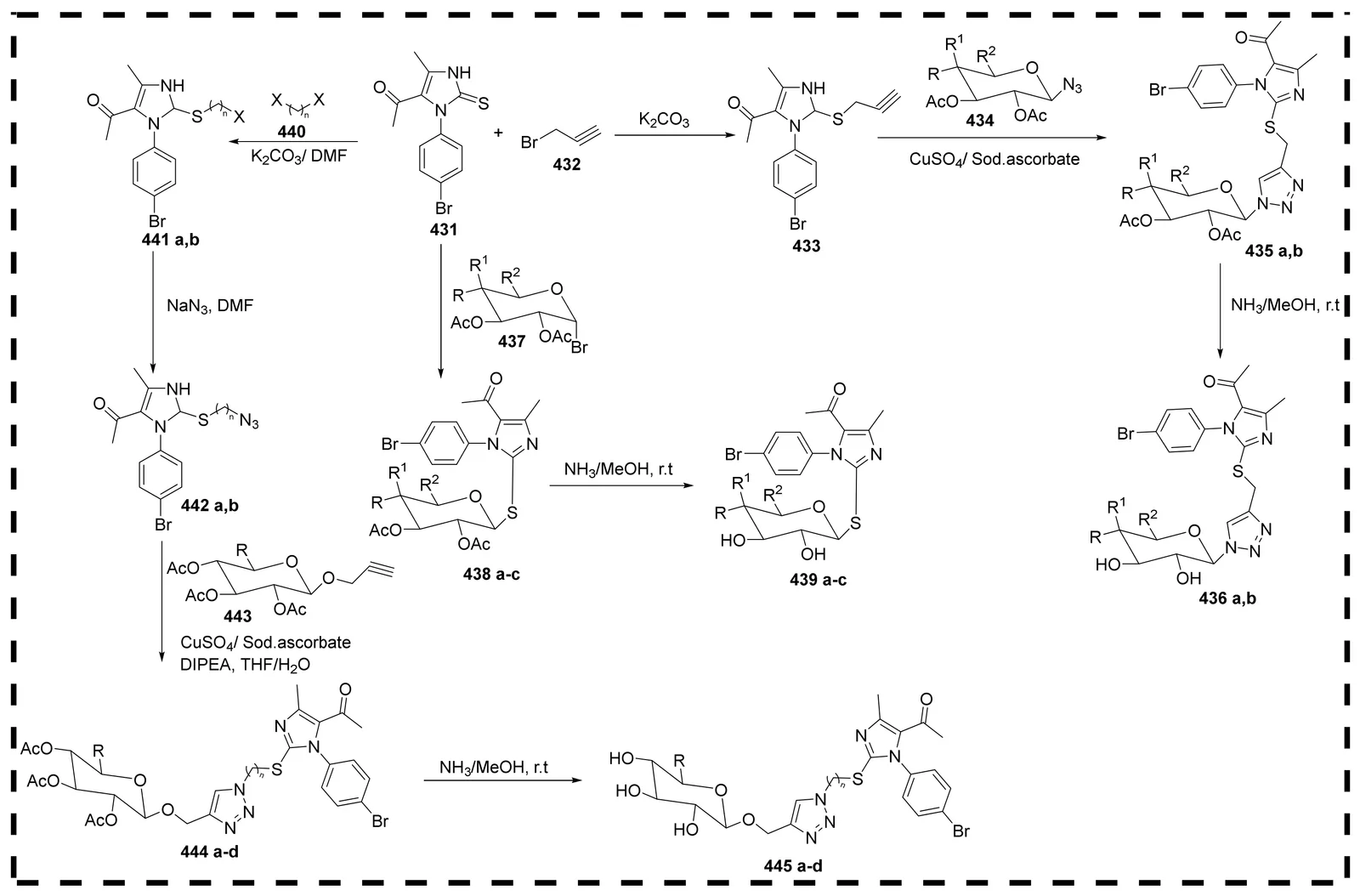
A multistep synthetic pathway to form imidazole-triazole-based glycosides.

**Scheme 25:**
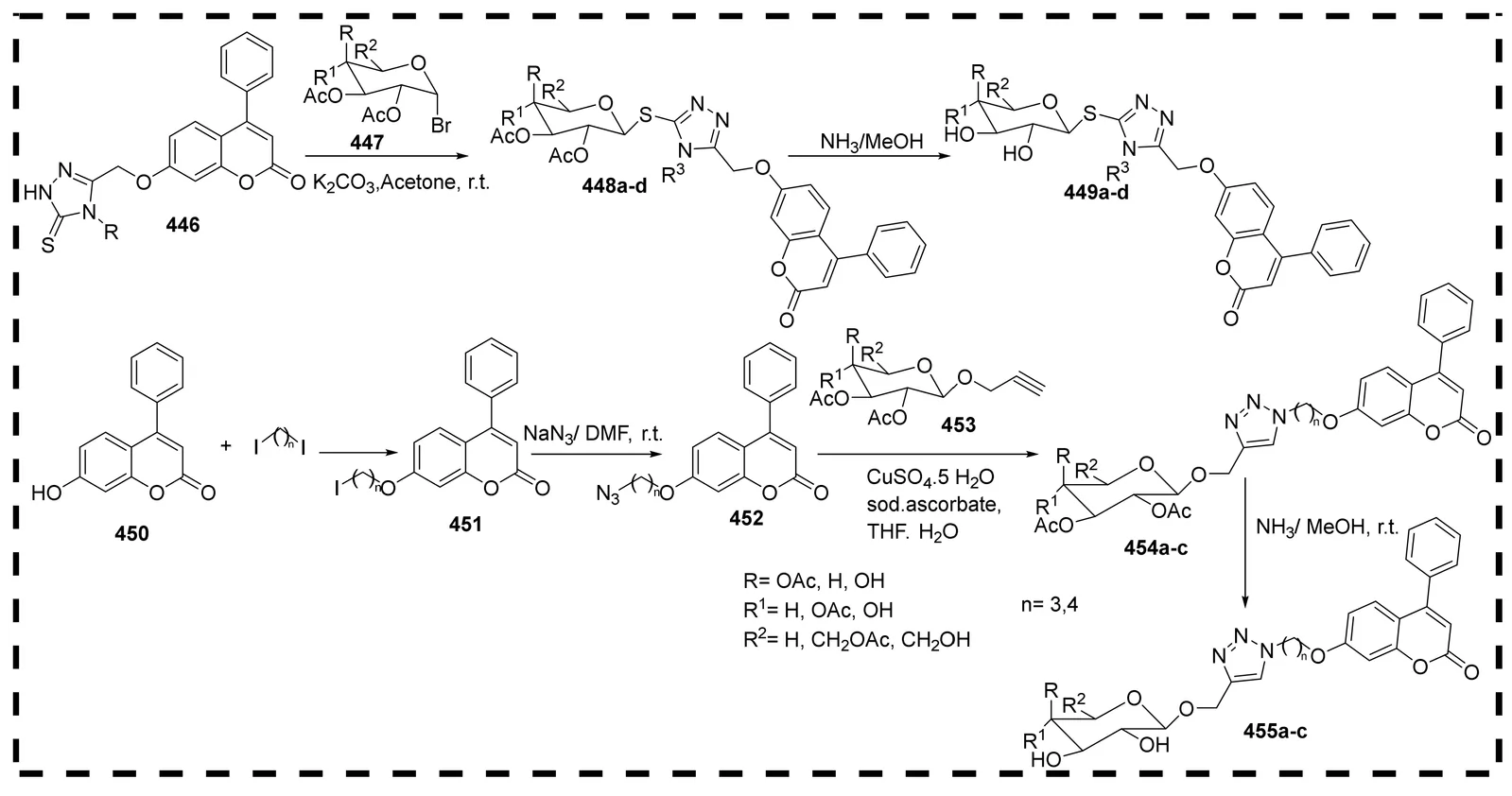
Formation of coumarin-based triazole-glycosyl hybrid and its thioglycoside.

**Scheme 26:**
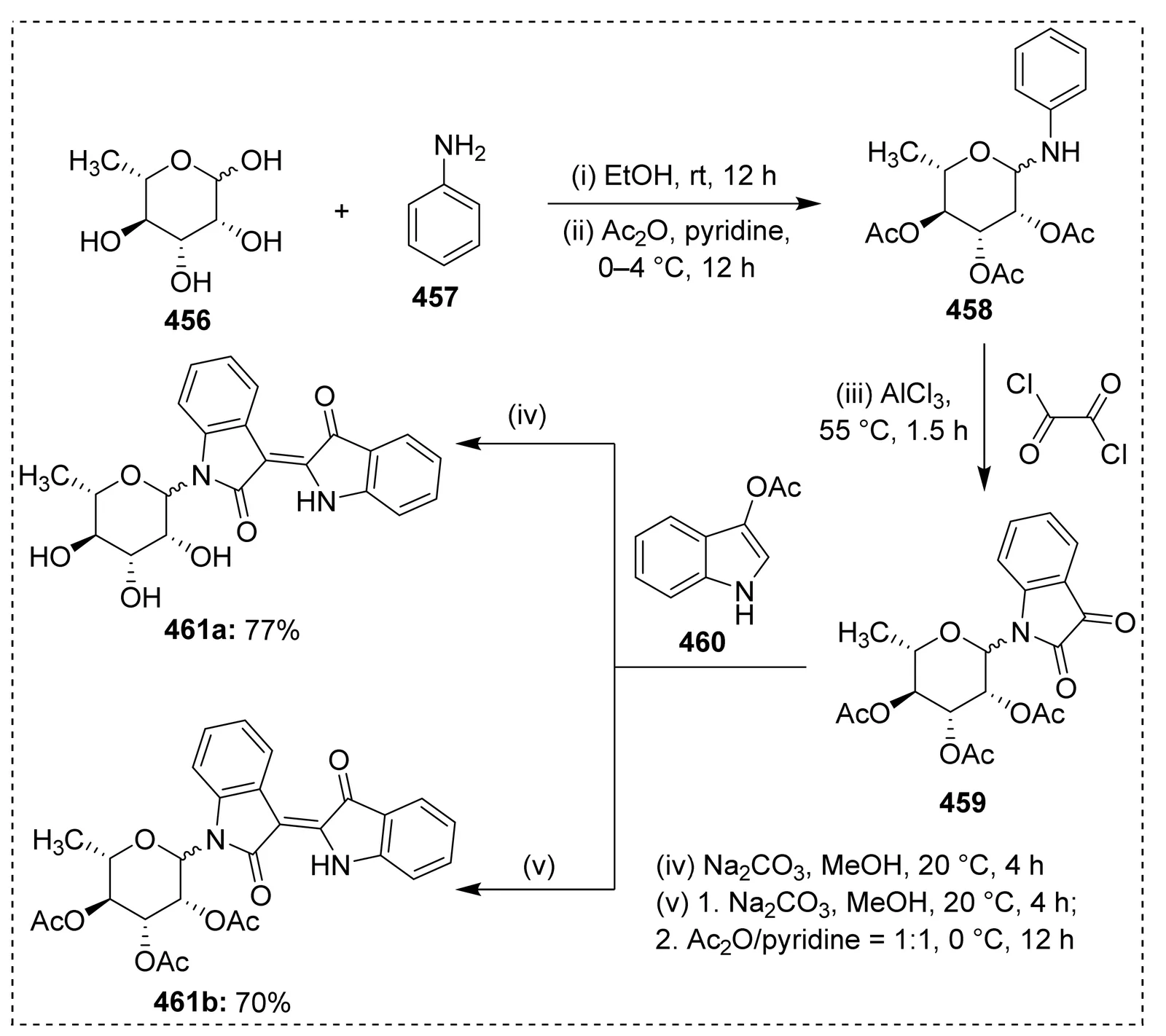
Synthesis of anticancer active indirubin-N′-glycosides.

**Scheme 27:**
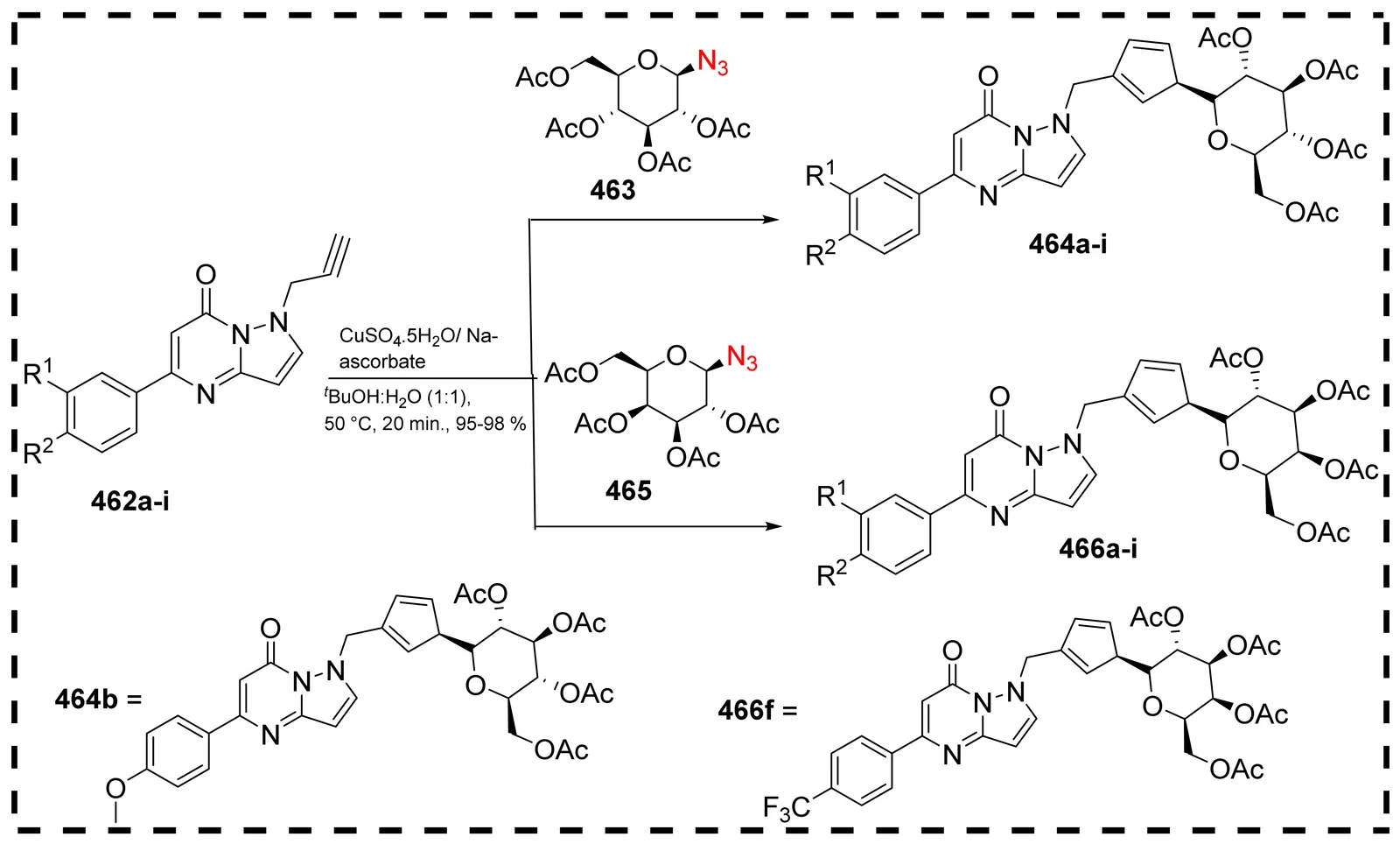
Microwave-assisted formation of triazole-bridged N-glycosides.

**Scheme 28:**
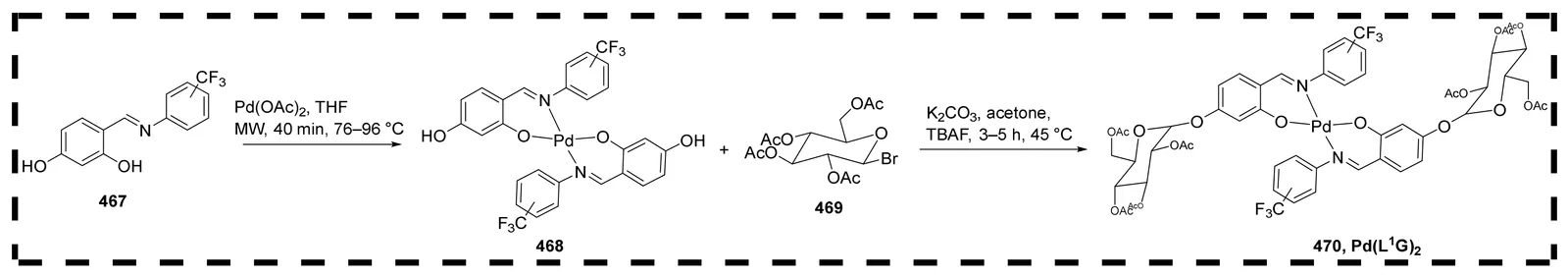
Synthesis of Pd and Cu glycoconjugate Schiff base complexes.

**Scheme 29:**
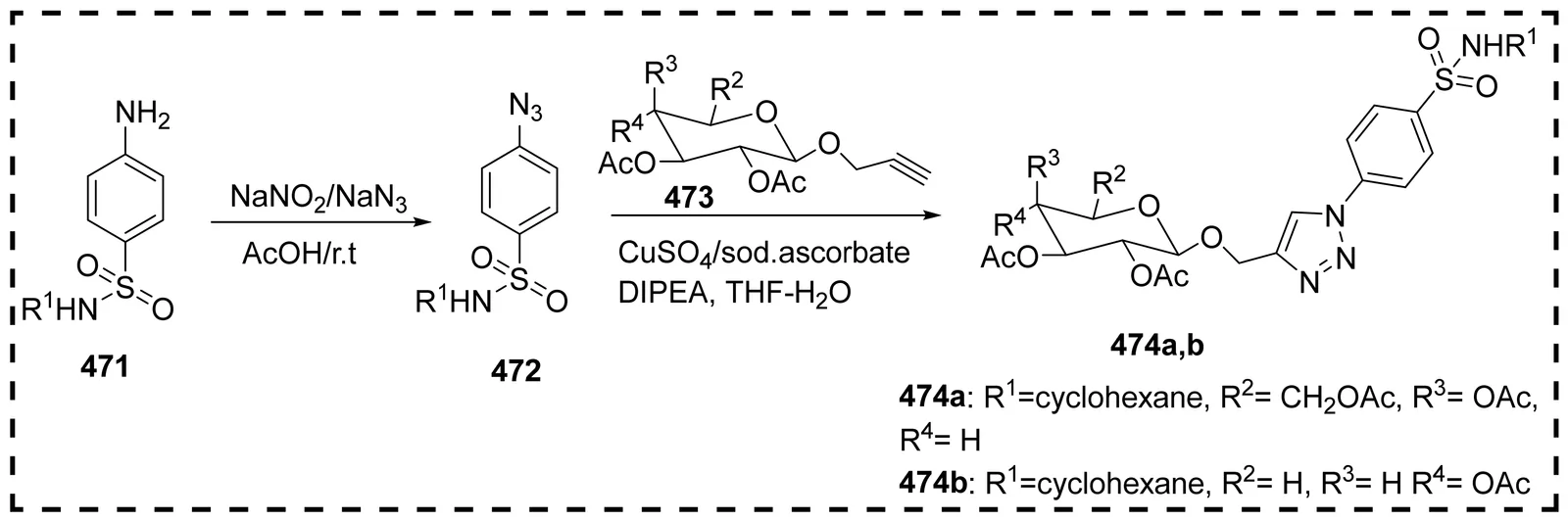
Synthesis of sulfonamide-triazole-glycoside hybrids.

**Scheme 30:**
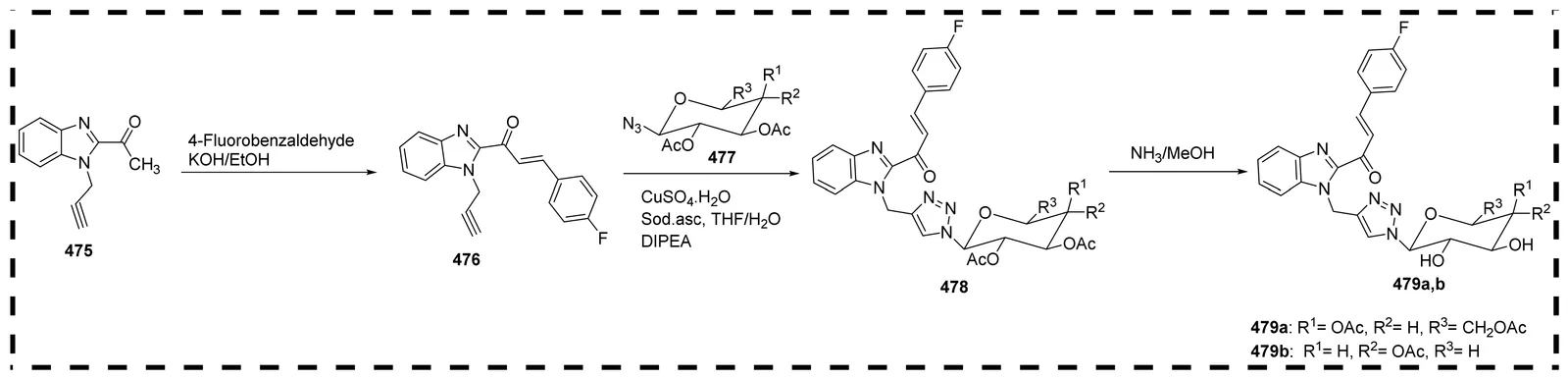
Synthesis of benzimidazole-triazole glycoconjugates.

**Scheme 31:**
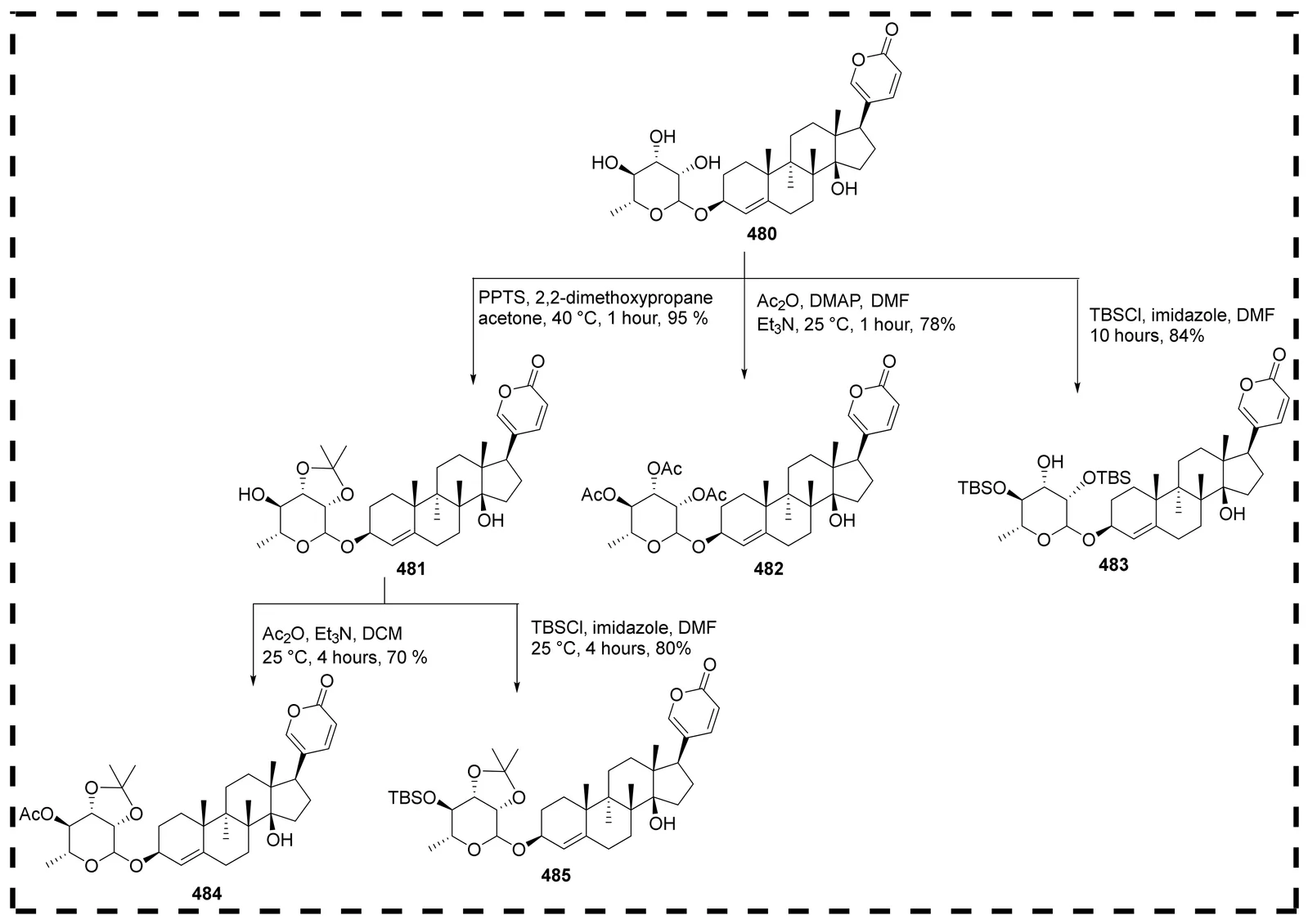
Synthesis of proscillaridin, a glycan analog.

**Scheme 32:**
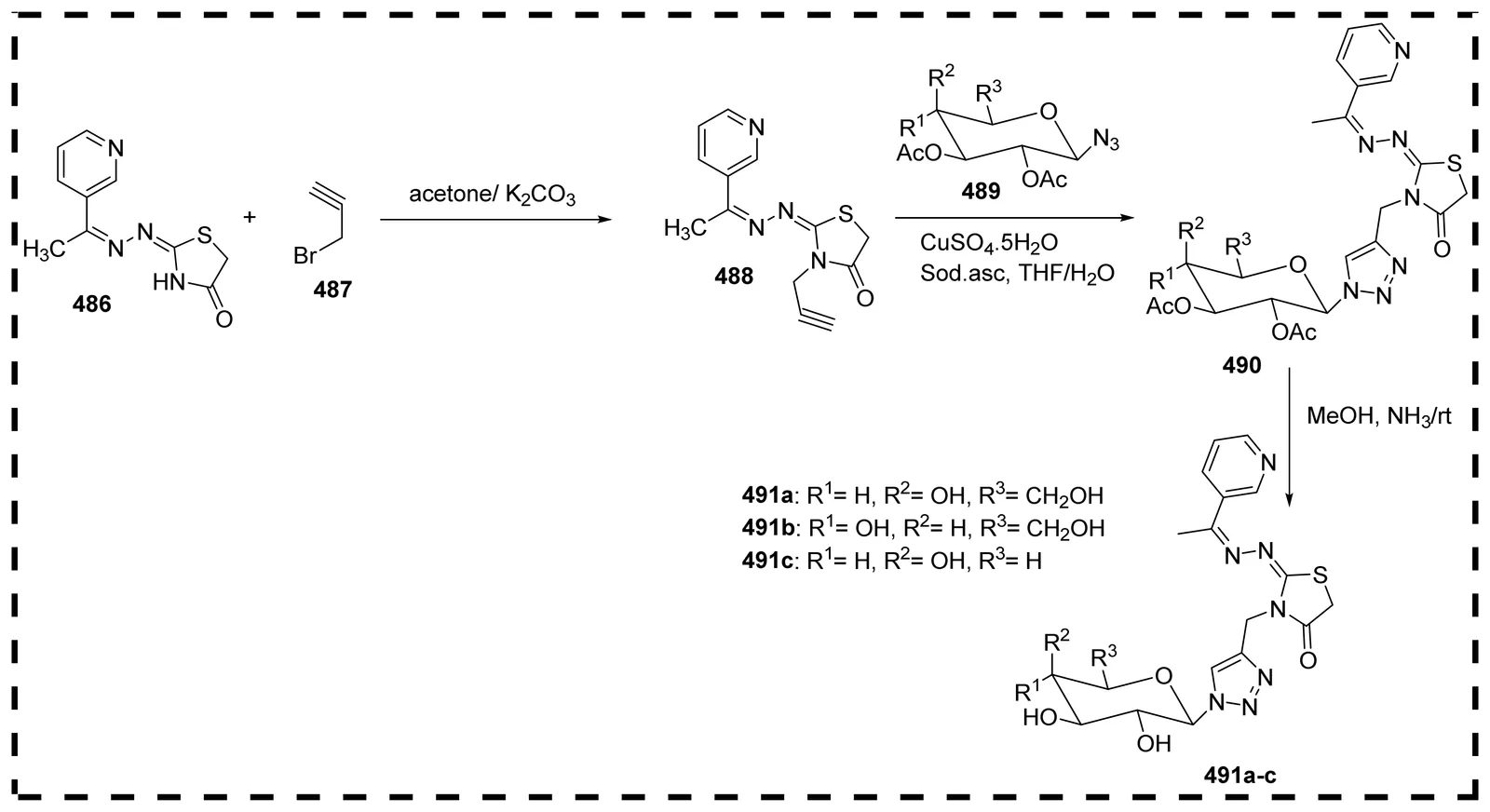
Synthesis of pyridine-thiazole-triazole glycosides.

**Scheme 33:**
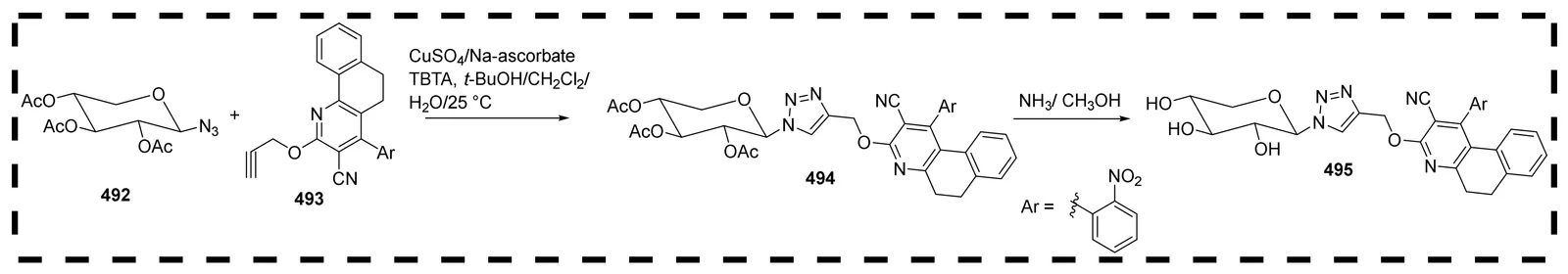
Synthesis of benzoquinoline-triazole N-glycosides.

**Scheme 34:**
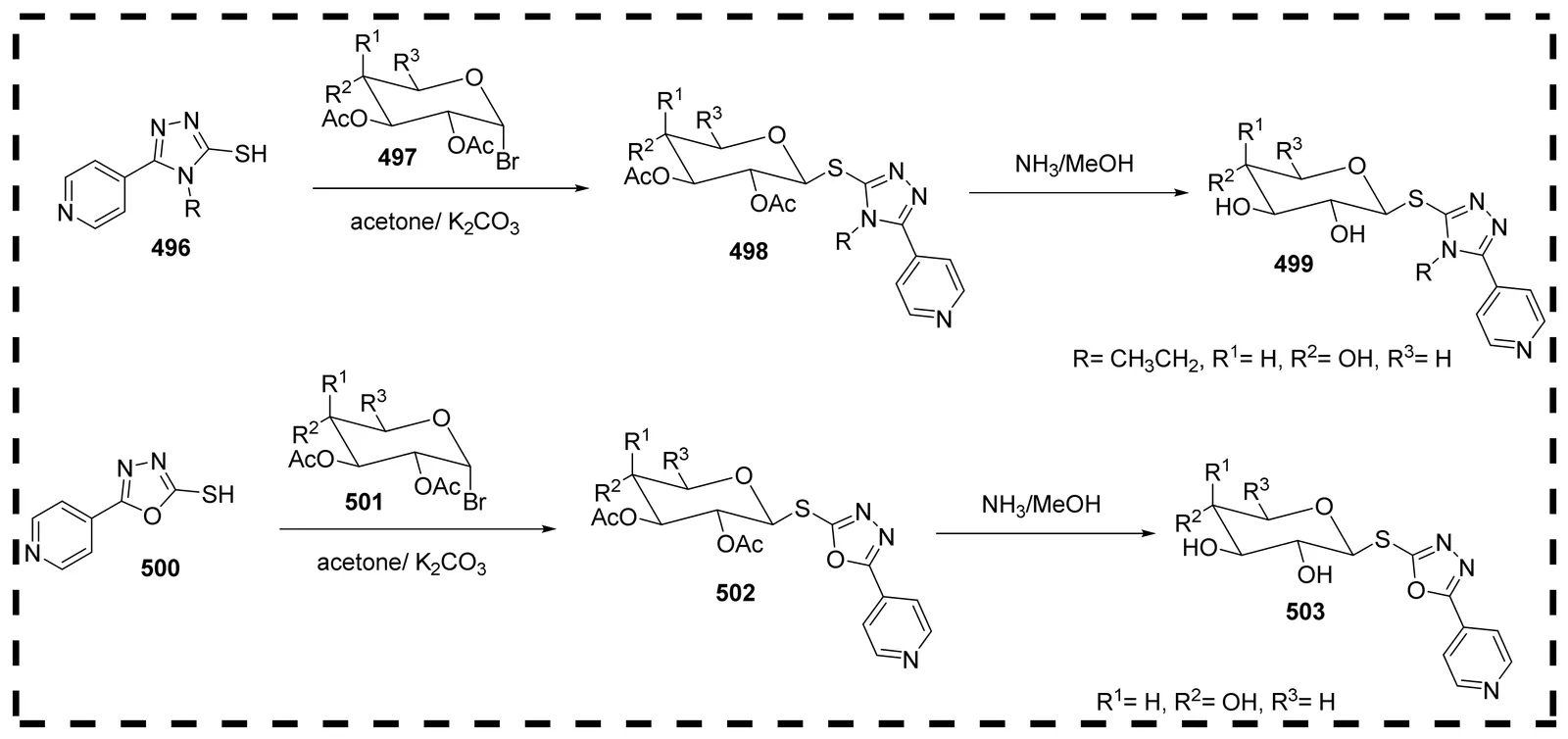
Synthesis of pyridine-triazole and oxadiazole glycosides.

**Table 1:** Summary of Steroid Glycosides with Anticancer Activity.

Most Potent Compounds	Source	Most Sensitive Cell Line	IC_50_ (µM)	Mechanism	Normal Cell Control	Ref.
Oleandrin (**5**)	*Nerium oleander*	SW480 (colon)	0.02	Mitochondrial pathway; ER stress	Yes (IC_50_ = 0.56)	[40]
Digitoxin (**4**)	*Digitalis*	NSCLC H1975	0.19	AMPK activation; Cell cycle arrest	Yes	[39]
Convallatoxin (**7**)	*Convallaria*	H460 (lung)	~0.1	Autophagy; EMT modulation	No	[28]
Peruvoside (**6**)	*Thevetia*	K562 (leukemia)	~0.1	Caspase pathway	Yes (no toxicity)	[43]
Proscillaridin A (**19**)	*Drimia maritima*	Caco-2 (colon)	0.9 µg/mL	ER stress	Yes (IC_50_ = 13.1)	[52]
Calotropin (**24**)	*Calotropis procera*	MDA-MB-231	0.046–0.072	G0/G1 arrest; Glycolysis inhibition	No	[57,58]
HTF-1 (**23**)	*Helleborus thibetanus*	Multiple	-	Caspase-9, MAPK, PI3K-Akt-mTOR	No	[56]
Ophiopogonin D (**32**)	*Ophiopogon*	NSCLC	-	STAT3 inhibition; GSH/GSSG imbalance	No	[63]

**Table 2:** Summary of Flavonoid and Polyphenol Glycosides with Anticancer Activity. N/A, not available.

Most Potent Compounds	Best IC_50_ Values	Cancer Types Targeted	Key Mechanisms	Normal Cell Control	Ref.
Hyperoside (**54**), Isoquercetin (**62**)	3.27 µM (MDA-MB-231)	Breast, lung, colon	EGFR inhibition; Apoptosis; G1/S arrest	No	[72,76]
Vicenin-II (**83**), Vitexin (**79**)	14.38 µg/mL (HepG2)	Liver, colon, cervical	Topoisomerase IIα; CDK2 inhibition	No	[100,102]
Naringenin-7-O-glucoside (**67**)	233.56 µg/µL (MDA-MB-231)	TNBC, gastric	Apoptosis; DNA fragmentation	Yes (Vero)	[94]
Cyanidin-3-glucoside (**66**)	10–15 mg/kg (in vivo)	Colorectal	NF-κB suppression; Bax/Bcl-2	N/A	[93]
Ononin (**82**)	N/A	Colorectal	Ferroptosis; PI3K/AKT/Nrf2	N/A	[101]
Resveratrol glycoside (**77**)	N/A	Multiple	EGFR/VEGFR inhibition	No	[99]

**Table 3:** Summary of Terpene and Terpenoid Glycosides with Anticancer Activity.

Compound	Source	Most Sensitive Cell Line	IC_50_ (µM)	Mechanism	Ref.
Narcissiflorin (**95b**)	*Aralia armata*	A549, HT29	2.01	Cytotoxicity	[109]
Dioscin (**94**)	*Polygonatum*	Multiple	-	Anti-tumor immunity; Macrophage polarization	[106]
Glycyrrhizin (**98**)	*Glycyrrhiza*	Colorectal	-	MMP-9/MMP-2 inhibition; Apoptosis; Autophagy	[110]
β-Escin (**106**)	*Aesculus*	Multiple	-	p21 upregulation; Cyclin D1 reduction; Bcl-2 inhibition	[118]
Kutkoside (**119**)	*Picrorhiza*	Oral cancer	32 µM (apoptosis)	PI3K/AKT inhibition; Anti-migration	[122]
Aucubin (**126**)	*Verbascum*	MDA-MB-453	-	PI3K/AKT inhibition; Early apoptosis	[125]

**Table 4:** Summary of Other Glycosides with Anticancer Activity. ↑, increasing; ↓, decreasing.

Compound	Type	Most Sensitive Cell Line	IC_50_	Mechanism	Ref.
Amygdalin (**133**)	Cyanogenic glycoside	Prostate, cervical	-	Bax ↑, Bcl-2 ↓, Caspase-3 activation	[128]
Linamarin (**134**)	Cyanogenic glycoside	HeLa, MCF-7, HT-29	-	Mitochondrial respiratory chain	[134,135]
Verbascoside (**136**)	Phenylethanoid glycoside	MDA-MB-453, A549	200 µM	-	[138]
Bergenin (**144**)	C-glycoside	Cervical	-	Anti-angiogenesis; MMP-9 ↓, Galectin-3 ↓	[141]
Condurangogenin A (**129**)	Esteric glycoside	H460	32 µg/mL	DNA damage; p21/p53 modulation	[126]

**Table 5:** Comprehensive list of marine-derived natural compounds with their associated anti-cancer properties. NA, Not Available.

Anti-Cancer Compound Name	Source	Structure	Cancer Cell Type	Mechanism of Action	Ref.
Moromycin B (**152**)	*Marine actinomycetes*-*Streptomyces* sp. OC1610.4	Angucycline glycoside	Breast cancer cells MDA-MB-231, MCF-7, and BT-474 (IC_50_ value = 0.16–0.67 µM)	Inhibition of migration and cell invasion	[147]
Saquayamycin B1 (**153**)	*Marine actinomycetes*-*Streptomyces* sp. OC1610.4	Angucycline glycoside	Breast cancer cells MDA-MB-231, MCF-7, and BT-474 (IC_50_ value = 0.16–0.67 µM)	Inhibition of migration and cell invasion	[147]
Saquayamycin B (**154**)	*Marine actinomycetes*-*Streptomyces* sp. OC1610.4	Angucycline glycoside	Breast cancer cells MDA-MB-231, MCF-7, and BT-474 (IC_50_ value = 0.16–0.67 µM)	Inhibition of migration and cell invasion	[147]
Pseudopterosin H (**155**)	Marine coral *Pseudopterogorgia elisabethae*	Diterpene glycoside	Prostate cancer cell line	Causes apoptosis and suppresses the generation of reactive oxygen species inside cells.	[148]
Echinoside B (**156**)	Sea cucumber *Holothuria atra*	Sulfated Triterpene Glycoside	Ehrlich ascites carcinoma cells	NA	[149]
24-Dehydroechinoside B (**157**)	Sea cucumber *Holothuria atra*	Sulfated Triterpene Glycoside	Ehrlich ascites carcinoma cells	NA	[149]
Coloquadranoside A (**158**)	Sea cucumber *Colochirus quadrangularis*	Sulfated triterpene glycoside	Eight human tumor cell lines with IC_50_ ranging from 0.46 to 2.03 μM. (MKN-28, HCT-116, A-549, HO-8910, MCF-7, BEL-7402, HL-60, SPC-A4)	NA	[150]
Urdamycins W (**159**)	Actinobacterium *Streptomyces ardesiacus*	Angucycline glycoside	NUGC-3 (stomach), PC-3 (prostate), ACHN (renal), MDA-MB-231 (breast), HCT-15 (colon), NCI-H23 (lung) GI_50_ = 0.019–0.104 µM	NA	[151]
Magnumosides A3 (**160**)	Sea cucumber *Neothyonidium*	Sulfated triterpene glycoside	Mouse Ehrlich carcinoma cells, human colorectal carcinoma DLD-1 cells	Reduced the size and prevented the development of DLD-1 cancer cell colonies at non-cytotoxic concentrations.	[152]
Magnumosides C1 (**161**)	Sea cucumber *Neothyonidium*	Sulfated triterpene glycosides	Mouse Ehrlich carcinoma cells, human colorectal carcinoma DLD-1 cells	Reduced the size and prevented the development of DLD-1 cancer cell colonies at non-cytotoxic concentrations.	[152]
Hillaside C (**162**)	Sea cucumber *Holothuria hilla Lesson*	Triterpene glycoside	A-549, CAKI-1, KB-VIN, HCT-8, MCF-7, KB, IA9, and PC-3 (IC_50_ = 0.15–3.20 mg/mL)	NA	[153]
Philinopside E (**163**)	Sea cucumber *Pentacta quadrangularis*	Sulfated triterpene glycoside	MKN28, P388, HL60, SPC-A4, A549, SGC7901, BEL7402, W138, HO8901, A431 (Ed_50_ = 0.75–3.50 mg mL^−1^)	NA	[154]
Fuscocineroside C (**164**)	Sea cucumber *Holothuria fuscocinerea*	Triterpene Glycoside	HL-60 (IC_50_ = 0.88 ± 0.32), BEL-7402 (IC_50_ = 0.58 ± 0.05)	NA	[155]
Holothurin A_4_ (**165**)	Sea Cucumber *Holothuria scabra*	Triterpene Glycoside	KB (IC_50_ = 0.87 µg/mL) and Hep-G2 (IC_50_ = 0.32 µg/mL)	NA	[156]
Holothurin A_3_ (**166**)	Sea Cucumber *Holothuria scabra*	Triterpene Glycoside	KB (IC_50_ = 1.12 µg/mL) and Hep-G2 (IC_50_ = 0.57 µg/mL)	NA	[156]
Intercedenside A & C (**167a**, **167b**)	Sea cucumber *Mensamria intercedens Lampert*	Triterpene Glycoside	MCF-7, IA9, KB, U-87-MG, HCT-8, SK-MEL-2, A549, CAKI-1, PC-3, KB-VIN (IC_50_ = 0.96–4.0 µg/mL)	NA	[157]
Intercedenside B (**168**)	Sea cucumber *Mensamria intercedens Lampert*	Triterpene Glycosides	A549, MCF-7, IA9, KB-VIN, CAKI-1, U-87-MG, KB, PC-3, HCT-8, SK-MEL-2 (IC_50_ = 0.61–2.0 µg/mL)	NA	[157]
Philinopside B (**169**)	Sea cucumber *Pentacta quadrangularis*	Sulfated triterpene glycoside	KB-VIN, CAK1, HOS, U87-MG, IA9, KB, SK-MEL-2, PC3, HCT-8, A549 (ED_50_ = 0.75–3.00 µg/mL)	NA	[158]
Philinopside A (**170**)	Sea cucumber *Pentacta quadrangularis*	Sulfated triterpene glycoside	KB, CAK1, IA9, MEL-2, HOS, KB-VIN, A549, SK- HCT-8, U87-MG, PC3 (ED_50_ = 1.70–3.50 µg/mL)	NA	[158]
Holothurinoside A (**171**)	Sea cucumber *Holothuria forskalii*	Triterpene glycoside	P388, A549, HeLa, B-16 (IC_50_ = 0.33–2.0 µg/mL)	NA	[159]
Holothurinoside C (**172**)	Sea cucumber *Holothuria forskalii*	Triterpene glycoside	P388, A549, HeLa, B-16 (IC_50_ = 0.16–5.00 µg/mL)	NA	[159]
Arguside B (**173**)	Sea cucumber *Bohadschia argus Jaeger*	Triterpene Glycoside	A549 (IC_50_ = 0.48 ± 0.04 µg/mL), HCT-116 (IC_50_ = 0.46 ± 0.01 µg/mL), HepG2 (IC_50_ = 2.60 ± 0.28 µg/mL), and MCF-7 (IC_50_ = 1.64 ± 0.09 µg/mL)	NA	[160]
Arguside C (**174**)	Sea cucumber *Bohadschia argus Jaeger*	Triterpene Glycoside	A549 (IC_50_ = 0.53 ± 0.03 µg/mL), HCT-116 (IC_50_ = 0.92 ± 0.04 µg/mL), HepG2 (IC_50_ = 2.56 ± 0.29 µg/mL), and MCF-7 (IC_50_ = 1.55 ± 0.10 µg/mL)	NA	[160]
Culcinosides A (**175**)	Starfish *Culcita novaeguineae*	Polyhydroxysteroidal glycoside	U87 (IC_50_ = 9.35 ± 0.46 μM), U251 (IC_50_ = 11.28 ± 0.65 μM), SHG44 (IC_50_ = 8.04 ± 0.32 μM)	NA	[161]
Culcinosides B (**176**)	Starfish *Culcita novaeguineae*	Polyhydroxysteroidal glycoside	U87 (IC_50_ = 33.52 ± 1.23 μM), U251 (IC_50_ = 40.76 ± 1.58 μM), SHG44 (IC_50_ = 36.54 ± 1.44 μM)	NA	[161]
Culcinosides C (**177**)	Starfish *Culcita novaeguineae*	Polyhydroxysteroidal glycoside	U87 (IC_50_ = 26.33 ± 1.16 μM), U251 (IC_50_ = 22.66 ± 1.28 μM), SHG44 (IC_50_ = 35.26 ± 1.51 μM)	NA	[161]
Culcinosides D (**178**)	Starfish *Culcita novaeguineae*	Polyhydroxysteroidal glycoside	U87 (IC_50_ = 43.25 ± 1.73 μM), U251 (IC_50_ = 28.93 ± 1.83 μM), SHG44 (IC_50_ = 26.22 ± 1.64 μM)	NA	[161]
Echinoside A (**179**)	*Holothuria scabra Jaeger* and *Cucumaria frondosa Gunnerus*, *Peasonothuria graeffei*, *Holothuria nobilis*	Triterpene glycoside	HeLa, K562, HepG2, HL-7702 (IC_50_ = 1.25 ± 0.16–3.12 ± 0.65 μg/mL) 2.7 μM (HepG2 cells), 2.4 μM (human cancer cell lines)	Decreased the Bcl-2/Bax mRNA expression ratio and Δψm, and also up-regulated the mRNA expression levels of Caspase (3, 8 and 9), apoptosis induction and cell cycle arrest	[162,163,164]
Luzonicoside A (**180**)	Starfish *Echinaster luzonicus*	Steroid glycoside	RPMI-7951 and SK-Mel-28	Regulation of the cell cycle and induction of apoptosis, controlling the activity of poly (ADP-ribose) polymerase (PARP), cyclin D1, Bcl-2, and p21 levels	[165]
Luzonicoside D (**181**)	Starfish *Echinaster luzonicus*	Steroid glycoside	RPMI-7951 and SK-Mel-28	Regulation of the cell cycle and induction of apoptosis, controlling the activity of poly(ADP-ribose) polymerase (PARP), cyclin D1, Bcl-2, and p21 levels	[165]
Stichoposide D (**182**)	*Thelenota anax*	Triterpene glycoside	1.5 μM (HL-60 cells)	Induction of apoptosis, Ceramide production, activation of ceramide synthase 6, and extrinsic and intrinsic pathways	[166]
Stichoposide C (**183**)	*Thelenota anax*	Triterpene glycoside	0.3 μM (HL-60 cells) 0.5 μM (K562 cells)	Induction of apoptosis, formation of ceramides, activation of acid SMase and neutral SMase, and the extrinsic and intrinsic pathways	[167]
Saquayamycin B (**184**)	Intertidal sediments-derived *Streptomyces* sp.	Angucycline glycoside	HepG-2 (IC_50_ = 0.135 μM), SMMC-7721 (IC_50_ = 0.033 μM) and plc-prf-5 (IC_50_ = 0.244 μM)	NA	[168]
Frondoside A (**185**)	*Okinawa propolis Cucumaria frondosa*	Triterpene glycoside	Cancer cell lines of different cancer types (breast, leukemia, lung, prostate, colorectal, and pancreas)	NA	[169]
Nobiliside D (**186**)	*Holothuria nobilis*	Triterpene glycoside	K562 (IC_50_ = 0.83 ± 0.14 µg/mL), U937, A-549, HeLa, MCF-7 (IC_50_ = 0.82 ± 0.11 µg/mL), and HepG2	NA	[170]
Pentactaside B (**187**)	*Pentacta quadrangularis*	Disulfated triterpene glycoside	MCF-7, HCT-116, P-388, A-549, MKN-28 (IC_50_ = 0.09 ± 0.02–1.56 ± 0.22 µM)	NA	[171]
Pentactaside C (**188**)	*Pentacta quadrangularis*	Disulfated triterpene glycoside	MCF-7, HCT-116, P-388, A-549, MKN-28 (IC_50_ = 0.58 ± 0.05–2.30 ± 0.20 µM)	NA	[171]
Stichoposide A (**189**)	Vietnamese sea cucumber *S. horrens*	Triterpene diglycosides	LNCaP, MCF7, KB, Hep-G2, SK-Mel2 (IC_50_ = 2.12 ± 0.30–3.02 ± 0.33 µM)	NA	[172]
Stichoposide B (**190**)	Vietnamese sea cucumber *S. horrens*	Triterpene diglycoside	LNCaP, MCF7, KB, Hep-G2, SK-Mel2 (IC_50_ = 5.28 ± 0.25–8.86 ± 0.24 µM)	NA	[172]
Stichorrenoside C (**191**)	Vietnamese sea cucumber *S. horrens*	Triterpene diglycoside	LNCaP, MCF7, KB, Hep-G2, SK-Mel2 (IC_50_ = 7.03 ± 0.88–11.00 ± 0.20 µM)	NA	[172]
Stichorrenoside D (**192**)	Vietnamese sea cucumber *S. horrens*	Triterpene diglycoside	MCF7, LNCaP, SK-Mel2, KB, Hep-G2, (IC_50_ = 1.92 ± 0.61–3.13 ± 0.40 µM)	NA	[172]
3β-O-[β-D-xylopyranosyl-(1 → 2)-β-D-xylopyranosyl]-23S-acetoxyholost-7-ene (**193**)	Vietnamese sea cucumber *S. horrens*	Triterpene diglycosides	LNCaP, MCF7, KB, Hep-G2, SK-Mel2 (IC_50_ = 2.04 ± 0.73–3.11 ± 0.32 µM)	NA	[172]
Okhotosides B1 (**194**)	Sea cucumber (*Cucumaria okhotensis*)	Oligoglycoside	HeLa (IC_50_ = 34.0 µg/mL)	NA	[173]
Okhotosides B2 (**195**)	Sea cucumber (*Cucumaria okhotensis*)	Oligoglycoside	HeLa (IC_50_ = 13.0 µg/mL)	NA	[173]
Okhotosides B3 (**196**)	Sea cucumber (*Cucumaria okhotensis*)	Oligoglycoside	HeLa (IC_50_ = 17.8 µg/mL)	NA	[173]
Holothurin A1 (**197**)	Sea cucumber *Pearsonothuria graeffei*	Sulfated triterpene glycoside	HepG2, ECV-304	Suppression of MMP-9 and VEGF, and an increase in TIMP-1 expression	[174]
24-Dehydroechinoside A (**198**)	Sea cucumber *Pearsonothuria graeffei*	Sulfated triterpene glycoside	HepG2, ECV-304	Suppression of MMP-9 and VEGF, and an increase in TIMP-1 expression	[174]
Crassarosteroside C (**199**)	Formosan soft coral *Sinularia crassa*	Steroidal glycoside	HepG2 (IC_50_ = 17.6 μM), HepG3 (IC_50_ = 18.9 μM)	NA	[175]
Inornatoside B (**200**)	Sea cucumber *Holothuria inornata*	Sulfated triterpene glycoside	MCF-7, SKLU-1 (IC 50 = 0.47–0.50 Mm)	NA	[176]
Frondoside A **(201)**	Sea cucumber (*Cucumaria frondose*)	Triterpene glycoside	HepG2, Panc02, UM-UC-3	(Inhibition of CDK1, TOP2A, CDC20, KIF20A), Increases FLNC expression	[177]
Okhotoside A1–1 (**202**)	Far Eastern Sea cucumber *Cucumaria djakonovi*	monosulfated triterpene glycoside	MDA-MB-231 (IC_50_ = 2.34 ± 0.53 µM)	Inhibited migration, colony formation, and growth in MDA-MB-231 cells	[178]
Cucumarioside A0–1 (**203**)	Far Eastern Sea cucumber *Cucumaria djakonovi*	monosulfated triterpene glycosides	MDA-MB-231(IC_50_ = 6.04 ± 0.47 µM)	Inhibited migration, colony formation, and growth in MDA-MB-231 cells	[178]
Avicenoside A (**204**)	*Avicennia marina*	iridoid glycoside	HepG2 and A549	NA	[179]
Avicenoside B (**205**)	*Avicennia marina*	iridoid glycoside	HepG2 and A549	NA	[179]
Chrysomycin A (**206**)	Marine bacterium *Streptomyces* sp.	C-glycoside polyketides	U251 and U87-MG	Apoptosis induction (Bax/Bcl-2 balance, caspase 3/7 activation), Akt/GSK-3β pathway downregulation.	[180]
Iezoside (**207**)	Marine cyanobacterial assemblage	-	A549 (IC_50_ = 1.5 Mm), HeLa (IC_50_ = 1.0 μM)	Increase in cytosolic calcium, SERCA Inhibition.	[181]
Iezoside B (**208**)	Marine cyanobacterial assemblage	-	A549 (IC50 = 3.0 μM), HeLa (IC_50_ = 2.4 μM)	Increase in cytosolic calcium, SERCA Inhibition.	[181]
Djakonovioside E1 (**209**)	Sea cucumber *Cucumaria djakonovi*	trisulfated triterpene glycoside	ER-positive MCF-7, triple-negative MDA-MB-231	NA	[182]
Cucumarioside A2–5 (**210**)	Sea cucumber *Cucumaria djakonovi*	trisulfated triterpene glycoside	-	Inhibition of colony formation, reduction in tumor cell migration.	[178]
Pacificusoside M (**211**)	Pacific sun star *Solaster pacificus*	triterpene glycoside	HCT 116 cells	Suppresses colony formation	[183]
Cucumarioside A_2_–_2_ (**212**)	sea cucumber	triterpene glycoside	PC-3 human prostate cancer cells	G2/M cell cycle arrest, caspase-dependent intrinsic apoptosis, inhibition of colony formation/growth.	[183]
Psolusoside A (**213**)	sea cucumber *Psolus peronii*	sulfated triterpene glycoside	MDA-MB-231 cell	Inhibited colony growth and migration	[184]
Psolusoside L (**214**)	sea cucumber *Psolus peronii*	sulfated triterpene glycoside	T-47D cells, MDA-MB-231 cells	Inhibited colony growth and migration	[185]
Spiculiferoside C (**215**)	starfish *Henricia leviuscula spiculifera*	polyhydroxysteroid glycoside	HCT 116 cells	Induced G2/M cell cycle arrest; regulated CDK2, CDK4, cyclin D1, p21; inhibited phosphorylation of c-Raf, MEK1/2, ERK1/2	[186]
Conicospermiumoside A_3_–3 (**216**)	sea cucumber *Cucumaria conicospermium*	sulfated triterpene glycoside	MCF-7, T-47D, and MDA-MB-231	Colony formation/growth inhibition	[184]
Conicospermiumoside A_7_–1 (**217**)	sea cucumber *Cucumaria conicospermium*	sulfated triterpene glycoside	MCF-7, T-47D, and MDA-MB-231	Colony formation/growth inhibition	[184]
Lomaiviticin F (**218**)	*Micromonospora* sp. bacterium	benzofluorene glycoside	LNCaP, K562, NHDF, A549, MCF7, HCT-116, HeLa (IC_50_ = 5.4 to 20 μM)	NA	[187]
Lomaiviticin G (**219**)	*Micromonospora* sp. bacterium	benzofluorene glycoside	LNCaP, K562, NHDF, A549, MCF7, HCT-116, HeLa (IC_50_ = 5.75 to 20 μM)	NA	[187]
Lomaiviticin H (**220**)	*Micromonospora* sp. bacterium	benzofluorene glycoside	LNCaP, K562, NHDF, A549, MCF7, HCT-116, HeLa (IC_50_ = 9.79 to 20 μM)	NA	[187]
Cladoloside D (**221**)	Vietnamese sea cucumber *Cladolabes schmeltzii*	sulfated triterpene penta- and hexaosides	MCF-7, MDA-MB-231, MCF-10A	NA	[188]
Cladoloside H1 (**222**)	Vietnamese sea cucumber *Cladolabes schmeltzii*	sulfated triterpene penta- and hexaosides	MCF-7, MDA-MB-231, MCF-10A	NA	[188]
Holothurin B (**223**)	Sea cucumber *Holothuria tubulosa*	triterpene glycoside	PC-3 (IC_50_ = 1.22 ± 0.15 µM), PANC-1 (IC_50_ = 3.92 ± 0.35 µM)	Apoptosis-inducing activity	[189]
Cucumarioside C1 (**224**)	Sea star *Solaster pacificus*	triterpene glycoside	Prostate cancer cells	Activation of survival kinases (IKKα, IKKβ, IKKε), necroptosis (MLKL), metabolism (GCN2, PDK1), cytoskeletal dynamics (RHOK), mitophagy (PINK1), apoptosis(PITSLRE), immune modulation; inhibition of p38 & ERK1/2 MAP kinases	[190]

**Table 6:** Summary of Synthetic Glycosides with Anticancer Activity.

Glycoside Type	Most Potent Compounds	IC_50_ Range	Key Synthetic Approach	Advantages	Normal Cell Data
O-Glycosides	**318b**, **338b**, **338e**, **342**, **277f**	0.14–6.13 µM	Koenigs-Knorr; Enzymatic synthesis	Enhanced bioavailability; Increased metabolic stability	Yes (**277a–n**, **278**)
N-Glycosides	**359f**, **349b**, **350c**	2.2–9.8 µM	Click chemistry; CuAAC	Non-hydrolyzable; Resistant to glycosidases	Limited
S-Glycosides	**397a**, **389b**, **388b**	2.08–120.1 nM	Thio-glycosylation; S-alkylation	Excellent EGFR inhibition; High potency	Limited
Miscellaneous	**449b**, **449d**, **491c**, **503**	0.12–0.46 µM	Click chemistry; Metal complexation	Multi-targeting; Apoptosis induction	Yes (**460a–l**, **461a–l**)

## Data Availability

The data presented in this study are within the manuscript. All IC_50_ data with standard deviations and confidence intervals (where available from original studies) are compiled in Supplementary Table S1.

## Supplementary Materials

The following supporting information can be downloaded at: https://www.mdpi.com/article/10.3390/biom16101493/s1, Table S1: Comprehensive IC_50_ Data for Plant-Based and Synthetic Glycosides.

## References

[1] Liu Y., Huang L., Sun Q., Zhang M., Li T., Liang G., Pan W. (2014). Syntheses and Anti-Cancer Activities of Derivatives of Tetrandrine and Fangchinoline. Chem. Res. Chin. Univ..

[2] Tao Z.Q., Shi A.M., Wang K.X., Zhang W.D. (2015). Epidemiology of Prostate Cancer: Current Status. Eur. Rev. Med. Pharmacol. Sci..

[3] Kim C., Kim B. (2018). Anti-Cancer Natural Products and Their Bioactive Compounds Inducing ER Stress-Mediated Apoptosis: A Review. Nutrients.

[4] Demain A.L., Vaishnav P. (2011). Natural Products for Cancer Chemotherapy. Microb. Biotechnol..

[5] Molinski T.F., Dalisay D.S., Lievens S.L., Saludes J.P. (2009). Drug Development from Marine Natural Products. Nat. Rev. Drug Discov..

[6] Debbab A., Aly A.H., Lin W.H., Proksch P. (2010). Bioactive Compounds from Marine Bacteria and Fungi. Microb. Biotechnol..

[7] Proksch P., Putz A., Ortlepp S., Kjer J., Bayer M. (2010). Bioactive Natural Products from Marine Sponges and Fungal Endophytes. Phytochem. Rev..

[8] Gerwick W.H., Moore B.S. (2012). Lessons from the Past and Charting the Future of Marine Natural Products Drug Discovery and Chemical Biology. Chem. Biol..

[9] Reen F.J., Gutiérrez-Barranquero J.A., Dobson A.D.W., Adams C., O’Gara F. (2015). Emerging Concepts Promising New Horizons for Marine Biodiscovery and Synthetic Biology. Mar. Drugs.

[10] Jimenez P.C., Wilke D.V., Branco P.C., Bauermeister A., Rezende-Teixeira P., Gaudêncio S.P., Costa-Lotufo L.V. (2020). Enriching Cancer Pharmacology with Drugs of Marine Origin. Br. J. Pharmacol..

[11] Rotter A., Bacu A., Barbier M., Bertoni F., Bones A.M., Cancela M.L., Carlsson J., Carvalho M.F., Cegłowska M., Dalay M.C. (2020). A New Network for the Advancement of Marine Biotechnology in Europe and Beyond. Front. Mar. Sci..

[12] Chin Y.W., Balunas M.J., Chai H.B., Kinghorn A.D. (2006). Drug Discovery from Natural Sources. AAPS J..

[13] Patra S., Mishra S.R., Behera B.P., Mahapatra K.K., Panigrahi D.P., Bhol C.S., Praharaj P.P., Sethi G., Patra S.K., Bhutia S.K. (2022). Autophagy-Modulating Phytochemicals in Cancer Therapeutics: Current Evidences and Future Perspectives. Semin. Cancer Biol..

[14] Rejhová A., Opattová A., Čumová A., Slíva D., Vodička P. (2018). Natural Compounds and Combination Therapy in Colorectal Cancer Treatment. Eur. J. Med. Chem..

[15] Huang X.M., Yang Z.J., Xie Q., Zhang Z.-K., Zhang H., Ma J.-Y. (2019). Natural Products for Treating Colorectal Cancer: A Mechanistic Review. Biomed. Pharmacother..

[16] Seyed M.A., Ayesha S. (2021). Marine-Derived Pipeline Anticancer Natural Products: A Review of their Pharmacotherapeutic Potential and Molecular Mechanisms. Future J. Pharm. Sci..

[17] Nabil W.N.N., Dai R., Liu M., Xi Z., Xu H. (2024). Repurposing Cardiac Glycosides for Anticancer Treatment: A Review of Clinical Studies. Drug Discov. Today.

[18] El-Seedi H.R., Refaey M.S., Elias N., El-Mallah M.F., Albaqami F.M.K., Dergaa I., Du M., Salem M.F., Tahir H.E., Daglia M. (2025). Marine Natural Products as a Source of Novel Anticancer Drugs: An Updated Review (2019–2023). Nat. Prod. Bioprospect..

[19] Reddy D., Kumavath R., Barh D., Azevedo V., Ghosh P. (2020). Anticancer and Antiviral Properties of Cardiac Glycosides: A Review to Explore the Mechanism of Actions. Molecules.

[20] Shah K., Chhabra S., Chauhan N.S. (2022). Chemistry and Anticancer Activity of Cardiac Glycosides: A Review. Chem. Biol. Drug Des..

[21] Ali S., Rukh G., Ogaly H.A., Ajaj R., Rauf A., Shah S.A.A. (2025). Synthesis and Anticancer Potential of Glycosides. RSC Adv..

[22] Bessen H.A. (1986). Therapeutic and Toxic Effects of Digitalis: William Withering, 1785. J. Emerg. Med..

[23] Krenn L., Kopp B. (1998). Bufadienolides from Animal and Plant Sources. Phytochemistry.

[24] Newman R.A., Yang P., Pawlus A.D., Block K.I. (2008). Cardiac Glycosides as Novel Cancer Therapeutic Agents. Mol. Interv..

[25] Fürstenwerth H. (2010). Ouabain—The Insulin of the Heart. Int. J. Clin. Pract..

[26] Prassas I., Diamandis E.P. (2008). Novel Therapeutic Applications of Cardiac Glycosides. Nat. Rev. Drug Discov..

[27] Gao H., Popescu R., Kopp B., Wang Z. (2011). Bufadienolides and their Antitumor Activity. Nat. Prod. Rep..

[28] Kaushik V., Yakisich J.S., Azad N., Kulkarni Y., Venkatadri R., Wright C., Rojanasakul Y., Iyer A.K.V. (2017). Anti-Tumor Effects of Cardiac Glycosides on Human Lung Cancer Cells and Lung Tumorspheres. J. Cell. Physiol..

[29] Sawadogo W.R., Schumacher M., Teiten M.H., Cerella C., Dicato M., Diederich M. (2013). A Survey of Marine Natural Compounds and Their Derivatives with Anti-Cancer Activity Reported in 2011. Molecules.

[30] Cheng C.S., Wang J., Chen J., Kuo K.T., Tang J., Gao H., Chen L., Chen Z., Meng Z. (2019). New Therapeutic Aspects of Steroidal Cardiac Glycosides: The Anticancer Properties of Huachansu and Its Main Active Constituent Bufalin. Cancer Cell Int..

[31] Kang X.H., Zhang J.H., Zhang Q.Q., Cui Y.-H., Wang Y., Kou W.-Z., Miao Z.-H., Lu P., Wang L.-F., Xu Z.-Y. (2017). Degradation of Mcl-1 through GSK-3β Activation Regulates Apoptosis Induced by Bufalin in Non-Small Cell Lung Cancer H1975 Cells. Cell. Physiol. Biochem..

[32] Zhao Y., Yan J., Han X., Niu F., Zhang J., Hu W. (2017). Anti-Proliferative Effect of Digoxin on Breast Cancer Cells via Inducing Apoptosis. Eur. Rev. Med. Pharmacol. Sci..

[33] Chou W.H., Liu K.L., Shih Y.L., Chuang Y.-Y., Chou J., Lu H.-F., Jair H.-W., Lee M.-Z., Au M.-K., Chung J.-G. (2018). Ouabain Induces Apoptotic Cell Death Through Caspase- and Mitochondria-Dependent Pathways in Human Osteosarcoma U-2 OS Cells. Anticancer Res..

[34] Du J., Jiang L., Chen F., Hu H., Zhou M. (2021). Cardiac Glycoside Ouabain exerts Anticancer Activity via Downregulation of STAT3. Front. Oncol..

[35] Einbond L.S., Shimizu M., Ma H., Wu H.-A., Goldsberry S., Sicular S., Panjikaran M., Genovese G., Cruz E. (2008). Actein Inhibits the Na^+^-K^+^-Atpase and Enhances the Growth Inhibitory Effect of Digitoxin on Human Breast Cancer Cells. Biochem. Biophys. Res. Commun..

[36] Einbond L.S., Wu H.A., Sandu C., Ford M., Mighty J., Antonetti V., Redenti S., Ma H. (2016). Digitoxin Enhances the Growth Inhibitory Effects of Thapsigargin and Simvastatin On ER-Negative Human Breast Cancer Cells. Fitoterapia.

[37] Hothi P., Martins T.J., Chen L., Deleyrolle L., Yoon J.-G., Reynolds B., Foltz G. (2012). High-Throughput Chemical Screens Identify Disulfiram as an Inhibitor of Human Glioblastoma Stem Cells. Oncotarget.

[38] Lee D.H., Lee C.S., Kim D.W., Ae J.E., Lee T. (2014). Digitoxin Sensitizes Glioma Cells to TRAIL-Mediated Apoptosis by Upregulation of Death Receptor 5 And Downregulation of Survivin. Anti-Cancer Drugs.

[39] Zhang Y.Z., Chen X., Fan X.X., He J.X., Huang J., Xiao D.K., Zhou Y.L., Zheng S.Y., Xu J.H., Yao X.J. (2016). Compound Library Screening Identified Cardiac Glycoside Digitoxin as an Effective Growth Inhibitor of Gefitinib-Resistant Non-Small Cell Lung Cancer Via Downregulation of α-Tubulin and Inhibition of Microtubule Formation. Molecules.

[40] Pan L., Zhang Y., Zhao W., Zhou X., Wang C., Deng F. (2017). The Cardiac Glycoside Oleandrin Induces Apoptosis in Human Colon Cancer Cells via the Mitochondrial Pathway. Cancer Chemother. Pharmacol..

[41] Li X.X., Wang D.Q., Sui C.G., Meng F.-D., Sun S.-L., Zheng J., Jiang Y.-H. (2020). Oleandrin Induces Apoptosis via Activating Endoplasmic Reticulum Stress in Breast Cancer Cells. Biomed. Pharmacother..

[42] Li X.L., Ma R.H., Ni Z.J., Thakur K., Cespedes-Acuña C.L., Wang S., Zhang J.-G., Wei Z.-J. (2021). Dioscin Inhibits Human Endometrial Carcinoma Proliferation via G0/G1 Cell Cycle Arrest and Mitochondrial-Dependent Signaling Pathway. Food Chem. Toxicol..

[43] Feng Q., Leong W.S., Liu L., Chan W. (2016). Peruvoside, A Cardiac Glycoside, Induces Primitive Myeloid Leukemia Cell Death. Molecules.

[44] Schneider N.F.Z., Cerella C., Lee J.-Y., Mazumder A., Kim K.R., de Carvalho A., Munkert J., Pádua R.M., Kreis W., Kim K.-W. (2018). Cardiac Glycoside Glucoevatromonoside Induces Cancer Type-Specific Cell Death. Front. Pharmacol..

[45] Schneider N.F.Z., Menegaz D., Dagostin A.L.A., Persich L., Rocha S.C., Ramos A.C.P., Cortes V.F., Fontes C.F.L., de Pádua R.M., Munkert J. (2021). Cytotoxicity of Glucoevatromonoside Alone and in Combination with Chemotherapy Drugs and their Effects on Na^+^,K^+^-Atpase and Ion Channels on Lung Cancer Cells. Mol. Cell. Biochem..

[46] Zhang B.D., Zhu W.F., Akihisa T., Kikuchi T., Ukiya M., Maya F., Xu J., Liu W.-Y., Feng F., Zhang J. (2021). Cardiac Glycosides from the Roots of Streblus Asper Lour. and their Apoptosis-Inducing Activities in A549 Cells. Phytochemistry.

[47] Terzioglu-Usak S., Nalli A., Elibol B., Ozek E., Hatiboglu M.A. (2020). Anvirzel™ Regulates Cell Death Through Inhibiting GSK-3 Activity in Human U87 Glioma Cells. Neurol. Res..

[48] Ren Y., Tan Q., Heath K., Wu S., Wilson J.R., Ren J., Shriwas P., Yuan C., Ngoc Ninh T., Chai H.B. (2020). Cytotoxic and Non-Cytotoxic Cardiac Glycosides Isolated from the Combined Flowers, Leaves, and Twigs of Streblus asper. Bioorg. Med. Chem..

[49] Liu M., Huang Q., A J., Li L., Li X., Zhang Z., Dong J.-T. (2021). The Cardiac Glycoside Deslanoside Exerts Anticancer Activity in Prostate Cancer Cells by Modulating Multiple Signaling Pathways. Cancers.

[50] Weng J.R., Lin W.Y., Bai L.Y., Hu J.-L., Feng C.-H. (2020). Antitumor Activity of the Cardiac Glycoside A-L-Diginoside by Modulating Mcl-1 in Human Oral Squamous Cell Carcinoma Cells. Int. J. Mol. Sci..

[51] Wang F., Liu L., Tong Y., Li L., Liu Y., Gao W.-Q. (2020). Proscillaridin A Slows the Prostate Cancer Progression Through Triggering the Activation of Endoplasmic Reticulum Stress. Cell Cycle.

[52] Al-Abdallat K., Obeidat M., Ababneh N.A., Zalloum S., Al Hadidi S., Al-Abdallat Y., Zihlif M., Awidi A. (2023). Phytochemical Analysis and Anticancer Properties of Drimia Maritima Bulb Extracts on Colorectal Cancer Cells. Molecules.

[53] Leu W.J., Wang C.T., Hsu J.L., Chen I., Chang H., Guh J. (2020). Ascleposide, A Natural Cardenolide, Induces Anticancer Signaling in Human Castration-Resistant Prostatic Cancer Through Na^+^/K^+^-Atpase Internalization and Tubulin Acetylation. Prostate.

[54] An N., Sun Y., Ma L., Shi S., Zheng X., Feng W., Shan Z., Han Y., Zhao L., Wu H. (2020). Helveticoside Exhibited P53-Dependent Anticancer Activity Against Colorectal Cancer. Arch. Med. Res..

[55] Reddy D., Ghosh P., Kumavath R. (2020). Strophanthidin Attenuates MAPK, PI3K/AKT/Mtor, and Wnt/Β-Catenin Signaling Pathways in Human Cancers. Front. Oncol..

[56] Ma L., Meng Y., Tu C., Cao X., Wang H., Li Y., Man S., Zhou J., Li M., Liu Z. (2018). A Cardiac Glycoside HTF-1 Isolated from Helleborus Thibetanus Franch Displays Potent in Vitro Anti-Cancer Activity via Caspase-9, MAPK and PI3K-Akt-Mtor Pathways. Eur. J. Med. Chem..

[57] Sweidan N., Esawi E., Ismail M., Alshaer W. (2021). Anticancer Cardenolides from the Aerial Parts of Calotropis procera. Z. Naturforsch. C.

[58] Rajkovic J., Novakovic R., Grujic-Milanovic J., Ydyrys A., Ablaikhanova N., Calina D., Sharifi-Rad J., Al-Omari B. (2023). An Updated Pharmacological Insight into Calotropin as a Potential Therapeutic Agent in Cancer. Front. Pharmacol..

[59] Hu Q.-Y., Zhang X.-K., Wang J.-N., Chen H.-X., He L.-P., Tang J.-S., Yao X.-S., Liu J. (2021). Malayoside, A Cardenolide Glycoside Extracted from Antiaris Toxicaria Lesch, Induces Apoptosis in Human Non-Small Lung Cancer Cells via MAPK-Nur77 Signaling Pathway. Biochem. Pharmacol..

[60] Hu Y., Yu K., Wang G., Zhang D., Shi C., Ding Y., Hong D., Zhang D., He H., Sun L. (2018). Lanatoside C Inhibits Cell Proliferation and Induces Apoptosis Through Attenuating Wnt/Β-Catenin/C-Myc Signaling Pathway in Human Gastric Cancer Cell. Biochem. Pharmacol..

[61] Rimpelová S., Zimmermann T., Drašar P.B., Dolenský B., Bejček J., Kmoníčková E., Cihlářová P., Gurská S., Kuklíková L., Hajdůch M. (2021). Steroid Glycosides Hyrcanoside and Deglucohyrcanoside: On Isolation, Structural Identification, and Anticancer Activity. Foods.

[62] Mukhtar M., Khan S., Riaz N., Tousif M.I., Nazir M., Jabbar A., Saleem M. (2020). Anticancer Agents from Solanum surattense. Front. Chem. Sci..

[63] Lee J.H., Kim C., Lee S.G., Sethi G., Ahn K.S. (2018). Ophiopogonin D, A Steroidal Glycoside Abrogates STAT3 Signaling Cascade and Exhibits Anti-Cancer Activity by Causing GSH/GSSG Imbalance in Lung Carcinoma. Cancers.

[64] Malyarenko O.S., Malyarenko T.V., Usoltseva R.V., Surits V.V., Kicha A.A., Ivanchina N.V., Ermakova S.P. (2021). Combined Anticancer Effect of Sulfated Laminaran from the Brown Alga Alaria angusta and Polyhydroxysteroid Glycosides from the Starfish Protoreaster lincki on 3D Colorectal Carcinoma HCT 116 Cell Line. Mar. Drugs.

[65] Qin W., Deng Y., Ren H., Liu Y., Liu L., Liu W., Zhao Y., Li C., Yang Z. (2024). Exploring the Anticancer Mechanism of Cardiac Glycosides using Proteome Integral Solubility Alteration Approach. Cancer Med..

[66] Harfita N.L., Santoni A., Suryati S. (2023). Beta-Sitosterol Glycoside from Paraboea Leuserensis and Cytotoxicity Test Against MCF-7 Human Breast Cancer Cells. Ris. Inf. Kesehat..

[67] Bawazeer M., Orabi M.A., Yaseen M., Abdelkader M.S.A. (2021). Cytotoxic Potential of Phenolic Glycosides from Stipagrostis plumosa. Rev. Bras. Farmacogn..

[68] Cuc N.T., Yen D.T.H., Yen P.H., Hang D.T.T., Tai B.H., Seo Y., Namkung W., Kim S.H., Van Cuong P., Van Kiem P. (2022). Dihydrostilbene Glycosides from Camellia Sinensis Var. Assamica and their Cytotoxic Activity. Nat. Prod. Res..

[69] Kim S.M., Han J.M., Le T.T., Sohng J.K., Jung H.J. (2020). Anticancer and Antiangiogenic Activities of Novel A-Mangostin Glycosides in Human Hepatocellular Carcinoma Cells via Downregulation of C-Met and HIF-1α. Int. J. Mol. Sci..

[70] Liu T., Li Y., Sun J., Tian G., Shi Z. (2020). Engeletin Suppresses Lung Cancer Progression by Inducing Apoptotic Cell Death Through Modulating the XIAP Signaling Pathway: A Molecular Mechanism Involving ER Stress. Biomed. Pharmacother..

[71] Elatif R.A., Shabana M., Fawzy L., Mansour R., Awad H., Sharaf M. (2020). Chemical Composition and Biological Activity of *Salicornia fruticosa* L.. Egypt. J. Chem..

[72] Li J.P., Liao X.H., Xiang Y., Yao A., Song R.-H., Zhang Z.-J., Huang F., Dai Z.-T., Zhang T.-C. (2018). Hyperoside and Let-7a-5p Synergistically Inhibit Lung Cancer Cell Proliferation via Inducing G1/S Phase Arrest. Gene.

[73] Hu Z., Zhao P., Xu H. (2020). Hyperoside Exhibits Anticancer Activity in Non-Small Cell Lung Cancer Cells with T790M Mutations by Upregulating Foxo1 Via CCAT1. Oncol. Rep..

[74] Ervina M., Poerwono H., Widyowati R., Matsunami K., Sukardiman S. (2020). Bio-Selective Hormonal Breast Cancer Cytotoxic and Antioxidant Potencies of *Melia azedarach* L. Wild Type Leaves. Biotechnol. Rep..

[75] Mohammed H.A., Khan R.A., Abdel-Hafez A.A., Abdel-Aziz M., Ahmed E., Enany S., Mahgoub S., Al-Rugaie O., Alsharidah M., Aly M.S.A. (2021). Phytochemical Profiling, In Vitro and In silico Anti-Microbial and Anti-Cancer Activity Evaluations and Staph Gyraseb and H-TOP-Iiβ Receptor-Docking Studies of Major Constituents of *Zygophyllum coccineum* L. Aqueous-Ethanolic Extract and its Subsequent Fractions: An Approach to Validate Traditional Phytomedicinal Knowledge. Molecules.

[76] Sameh S., Elissawy A.M., Al-Sayed E., Labib R.M., Chang H.-W., Yu S.-Y., Chang F.-R., Yang S.-C., Singab A.N.B. (2024). Family Malvaceae: A Potential Source of Secondary Metabolites with Chemopreventive and Anticancer Activities Supported with In Silico Pharmacokinetic and Pharmacodynamic Profiles. Front. Pharmacol..

[77] Joshi B.C., Juyal V., Sah A.N., Mukhija M. (2023). In vitro Antiproliferative Efficacy, Antioxidant Activity and LC-MS Based Metabolite Profiling of Premna Barbata Stem Bark. Def. Life Sci. J..

[78] Naso L., Martínez V.R., Lezama L., Salado C., Valcarcel M., Ferrer E.G., Williams P.A. (2016). Antioxidant, Anticancer Activities and Mechanistic Studies of the Flavone Glycoside Diosmin And Its Oxidovanadium(IV) Complex. Interactions with Bovine Serum Albumin. Bioorg. Med. Chem..

[79] Kuo K.W., Hsu S.H., Li Y.P., Lin W.L., Liu L.F., Chang L.C., Lin C.C., Lin C.N., Sheu H.M. (2000). Anticancer Activity Evaluation of the Solanum Glycoalkaloid Solamargine: Triggering Apoptosis in Human Hepatoma Cells. Biochem. Pharmacol..

[80] Liang C.H., Liu L.F., Shiu L.Y., Huang Y.-S., Chang L.-C., Kuo K.-W. (2004). Action of Solamargine on Tnfs and Cisplatin-Resistant Human Lung Cancer Cells. Biochem. Biophys. Res. Commun..

[81] Shiu L., Chang L., Liang C., Huang Y., Sheu H., Kuo K. (2007). Solamargine induces Apoptosis and Sensitizes Breast Cancer Cells to Cisplatin. Food Chem. Toxicol..

[82] Sun L., Zhao Y., Li X., Yuan H., Cheng A., Lou H. (2010). A Lysosomal–Mitochondrial Death Pathway is Induced by Solamargine in Human K562 Leukemia Cells. Toxicol. In Vitro.

[83] Li X., Zhao Y., Wu W.K., Liu S., Cui M., Lou H. (2011). Solamargine Induces Apoptosis Associated with P53 Transcription-Dependent and Transcription-Independent Pathways in Human Osteosarcoma U2OS Cells. Life Sci..

[84] Wu C.H., Liang C.H., Shiu L.Y., Chang L.C., Lin T.S., Lan C.C.E., Tsai J.C., Wong T.W., Wei K.J., Lin T.K. (2011). Solanum incanum Extract (SR-T100) Induces Human Cutaneous Squamous Cell Carcinoma Apoptosis Through Modulating Tumor Necrosis Factor Receptor Signaling Pathway. J. Dermatol. Sci..

[85] Cham B.E. (2013). Drug therapy: Solamargine and Other Solasodine Rhamnosyl Glycosides as Anticancer Agents. Mod. Chemother..

[86] Munari C.C., de Oliveira P.F., Campos J.C.L., Martins S.d.P.L., Da Costa J.C., Bastos J.K., Tavares D.C. (2014). Antiproliferative Activity of Solanum lycocarpum Alkaloidic Extract and their Constituents, Solamargine and Solasonine, in Tumor Cell Lines. J. Nat. Med..

[87] Wu Y.H., Chiu W.T., Young M.J., Chang T.-H., Huang Y.-F., Chou C.-Y. (2015). *Solanum incanum* Extract Downregulates Aldehyde Dehydrogenase 1-Mediated Stemness and Inhibits Tumor Formation in Ovarian Cancer Cells. J. Cancer.

[88] Xiang S., Zhang Q., Tang Q., Zheng F., Wu J., Yang L., Hann S.S. (2016). Activation of Ampkα Mediates Additive Effects of Solamargine and Metformin on Suppressing MUC1 Expression in Castration-Resistant Prostate Cancer Cells. Sci. Rep..

[89] Yu S., Sheu H.M., Lee C.H. (2017). *Solanum incanum* Extract (SR-T100) Induces Melanoma Cell Apoptosis and Inhibits Established Lung Metastasis. Oncotarget.

[90] Sun H., Lv C., Yang L., Wang Y., Zhang Q., Yu S., Kong H., Wang M., Xie J., Zhang C. (2014). Solanine Induces Mitochondria-Mediated Apoptosis in Human Pancreatic Cancer Cells. Biomed. Res. Int..

[91] Fu R., Wang X., Hu Y., Du H., Dong B., Ao S., Zhang L., Sun Z., Zhang L., Lv G. (2019). Solamargine Inhibits Gastric Cancer Progression by Regulating the Expression of Lncneat1_2 Via the MAPK Signaling Pathway. Int. J. Oncol..

[92] Wu J., Tang X., Ma C., Shi Y., Wu W., Hann S.S. (2020). The Regulation and Interaction of Colon Cancer-Associated Transcript-1 and miR7-5p Contribute to the Inhibition of SP1 Expression by Solamargine in Human Nasopharyngeal Carcinoma Cells. Phytother. Res..

[93] Wang M., Wang X. (2025). Chemoprotective Potential of Cyanidin-3-Glucoside Against 1,2-Dimethylhydrazine-Induced Colorectal Cancer: Modulation of NF-κB and Bcl-2/Bax/Caspase Pathway. J. Biochem. Mol. Toxicol..

[94] James A.R., Jayaprakash S., Sundeep L.M. (2024). In Vitro Cytotoxicity, Apoptotic Property, And Gene Expression Changes Induced by Naringenin-7-O-Glucoside in Triple-Negative Breast Cancer. Cureus.

[95] Tong J., Lifang L., Dong Z., Xu Z. (2023). Anticancer Effects of Hesperidin on Gastric Cancer Cell Lines and Fibroblast Cell Lines by Reducing the Activation of PI3K Pathway. Biomed. Res. Bull..

[96] Jamshidi S., Eghbalian A., Shojaei S., Taherkhani A., Feizi-Dehnayebi M. (2025). Flavonoids as Promising Akt1 Inhibitors in Cancer Medicine: Insights from Molecular Docking, Dynamics, DFT Calculations, And in Vitro Validation. Cancer Rep..

[97] Trivedi P.B., Badole M. (2024). Isolation of 5-Hydroxy-7-Methoxy-8-O-Β-D-Glucopyranosyl-2S-Flavanone from a New Source, Haplanthodes tentaculatus: Characterization and Anticancer Activity. Nat. Prod. Res..

[98] Sowa I., Mołdoch J., Paduch R., Strzemski M., Szkutnik J., Tyszczuk-Rotko K., Dresler S., Szczepanek D., Wójciak M. (2023). Polyphenolic Composition of *Carlina acaulis* L. Extract and Cytotoxic Potential Against Colorectal Adenocarcinoma and Cervical Cancer Cells. Molecules.

[99] Bouribab A., Karim E.M., Khedraoui M., Abchir O., Errougui A., Raouf Y.S., Samadi A., Chtita S. (2024). Exploring Moroccan Medicinal Plants for Anticancer Therapy Development through In Silico Studies. Pharmaceuticals.

[100] Fernández-Ochoa Á., Younis I.Y., Arafa R.K., Cádiz-Gurrea M.d.l.L., Leyva-Jiménez F.J., Carretero A.S., Mohsen E., Saber F.R. (2024). Metabolite Profiling of Colvillea Racemosa via UPLC-ESI-QTOF-MS Analysis in Correlation to the in vitro Antioxidant and Cytotoxic Potential Against A549 Non-Small Cell Lung Cancer Cell Line. Plants.

[101] Gui Y., Deng S., Li J., Zuo D., Fan H. (2026). Onion Induces Ferroptosis in Colorectal Cancer Cells Via the PI3K/AKT/Nrf2 Pathway to Enhance Anti-Cancer Immunotherapy. Naunyn-Schmiedeberg’s Arch. Pharmacol..

[102] Abdelrahim M.S., Abdel-Baky A.M., Bayoumi S.A., Backheet E.Y. (2023). Antioxidant and Antidiabetic Flavonoids from the Leaves of *Dypsis pembana* (H.E. Moore) Beentje & J. Dransf., Arecaceae: In vitro and Molecular Docking Studies. BMC Complement. Med. Ther..

[103] Abd-El-Aziz N.M., Hifnawy M.S., Lotfy R.A., Younis I.Y. (2024). LC/MS/MS and GC/MS/MS Metabolic Profiling of Leontodon hispidulus, in vitro and in silico Anticancer Activity Evaluation Targeting Hexokinase 2 Enzyme. Sci. Rep..

[104] Medjahed Z., Chaher-Bazizi N., Atmani-Kilani D., Ahmane N., Ruiz-Larrea M.B., Sanz J.I.R., Charid I., Amant F., Fonayet J.V., Saidene N. (2023). A Novel Flavonol Glycoside and Six Derivatives of Quercetin and Kaempferol from Clematis flammula with Antioxidant and Anticancer Potentials. Fitoterapia.

[105] Wei M.P., Zhu X.W., Yu H., Xie Y.-F., Guo Y.-H., Cheng Y.-L., Qian H., Yao W.-R. (2021). Isolation of Two Sesquiterpene Glycosides from Sapindus mukorossi Gaertn. with Cytotoxic Properties and Analysis of Their Mechanism Based on Network Pharmacology. Nat. Prod. Res..

[106] Cui L., Yang G., Ye J., Yao Y., Lu G., Chen J., Fang L., Lu S., Zhou J. (2020). Dioscin Elicits Anti-Tumour Immunity by Inhibiting Macrophage M2 Polarization via JNK and STAT3 Pathways in Lung Cancer. J. Cell. Mol. Med..

[107] Zhang Y.S., Ma Y.L., Thakur K., Hussain S.S., Wang J., Zhang Q., Zhang J.-G., Wei Z.-J. (2018). Molecular Mechanism and Inhibitory Targets of Dioscin in HepG2 Cells. Food Chem. Toxicol..

[108] Hernández-Vázquez J.M.V., López-Muñoz H., Escobar-Sánchez M.L., Flores-Guzmán F., Weiss-Steider B., Hilario-Martínez J.C., Sandoval-Ramírez J., Fernández-Herrera M.A., Sánchez Sánchez L. (2020). Apoptotic, Necrotic, and Antiproliferative Activity of Diosgenin and Diosgenin Glycosides on Cervical Cancer Cells. Eur. J. Pharmacol..

[109] Yen P.H., Chuong N.T.H., Lien G.T.K., Cuc N.T., Nhiem N.X., Thanh N.T.V., Tai B.H., Seo Y., Namkung W., Park S. (2021). Oleanane-Type Triterpene Saponins from *Aralia armata* Leaves and their Cytotoxic Activity. Nat. Prod. Res..

[110] Zhang Z., Xu M., Sun X., Guo X. (2020). Naturally Occurring Glycyrrhizin Triterpene Exerts Anticancer Effects on Colorectal Cancer Cells via Induction of Apoptosis and Autophagy and Suppression of Cell Migration and Invasion by Targeting MMP-9 And MMP-2 Expression. J. BUON.

[111] Rossi T., Benassi L., Magnoni C., Ruberto A.I., Coppi A., Baggio G. (2005). Effects of Glycyrrhizin on UVB-Irradiated Melanoma Cells. In Vivo.

[112] Haghshenas V., Fakhari S., Mirzaie S., Rahmani M., Farhadifar F., Peirzadeh S., Jalili A. (2014). Glycyrrhetinic Acid Inhibits Cell Growth and Induces Apoptosis in Ovarian Cancer A2780 Cells. Adv. Pharm. Bull..

[113] Khan R., Khan A.Q., Lateef A., Rehman M.U., Tahir M., Ali F., O Hamiza O., Sultana S. (2013). Glycyrrhizic Acid Suppresses the Development of Precancerous Lesions Via Regulating the Hyperproliferation, Inflammation, Angiogenesis and Apoptosis in the Colon of Wistar Rats. PLoS ONE.

[114] Routhier A., Astuccio M., Lahey D., Monfredo N., Johnson A., Callahan W., Partington A., Fellows K., Ouellette L., Zhidro S. (2010). Pharmacological Inhibition of Rho-Kinase Signaling with Y-27632 Blocks Melanoma Tumor Growth. Oncol. Rep..

[115] Ha D.T., Kicha A.A., Kalinovsky A.I., Malyarenko T.V., Popov R.S., Malyarenko O.S., Ermakova S.P., Thuy T.T.T., Long P.Q., Ivanchina N.V. (2021). Asterosaponins from the Tropical Starfish Acanthaster planci and their Cytotoxic and Anticancer Activities in vitro. Nat. Prod. Res..

[116] Sharma N., Kumar A., Sharma P., Qayum A., Singh S.K., Dutt P., Paul S., Gupta V., Verma M., Satti N. (2018). A New Clerodane Furano Diterpene Glycoside from Tinospora cordifolia Triggers Autophagy and Apoptosis in HCT-116 Colon Cancer Cells. J. Ethnopharmacol..

[117] Nath H., Sinha S., Rajkhowa S. (2022). Comparative Molecular Structure and Interaction Analysis of Some of the Glycoconjugates of Tinospora Isolate as Targeted Anti-Cancer Therapy: A DFT and Molecular Docking Study. Trends Carbohydr. Res..

[118] Patlolla J.M., Rao C.V. (2015). Anti-Inflammatory and Anti-Cancer Properties of β-escin, a Triterpene Saponin. Curr. Pharmacol. Rep..

[119] Öğütçü G., Tülay P., Kükner A., Çaliş İ., Şenol H. (2023). The effect of cycloartane-type saponins from Astragalus species on the proliferation of MCF-7 and MDA-MB-231 breast cancer cells. Turk. Onkol. Derg..

[120] Charalambous D., Christoforou M., Christou K., Christou M., Ververis A., Andreou M., Christodoulou K., Koutsoulidou A., Papachrysostomou C., Pantelidou M. (2024). Saponin and phenolic composition and assessment of biological activities of *Saponaria officinalis* L. root extracts. Plants.

[121] Elmaidomy A.H., El Zawily A., Salem A.K., Altemani F.H., Algehainy N.A., Altemani A.H., Rateb M.E., Abdelmohsen U.R., Shady N.H. (2023). New Cytotoxic Dammarane-Type Saponins from Ziziphus spina-christi. Sci. Rep..

[122] Hao Y., Zhang C., Sun Y., Xu H. (2019). Licochalcone A Inhibits Cell Proliferation, Migration, and Invasion Through Regulating the PI3K/AKT Signaling Pathway in Oral Squamous Cell Carcinoma. OncoTargets Ther..

[123] Kuhtinskaja M., Bragina O., Kulp M., Vaher M. (2020). Anticancer Effect of the Iridoid Glycoside Fraction from *Dipsacus fullonum* L. leaves. Nat. Prod. Commun..

[124] Shi L., Pi Y., Luo C., Zhang C., Tan D., Meng X. (2015). In vitro Inhibitory Activities of Six Gypenosides on Human Liver Cancer Cell Line HepG2 and Possible Role of HIF-1α Pathway in them. Chem.-Biol. Interact..

[125] Demirci S., Alp C., Akşit H., Ulutaş Y., Altay A., Yeniçeri E., Köksal E., Yaylı N. (2023). Isolation, Characterization and Anticancer Activity of Secondary Metabolites from Verbascum speciosum. Chem. Biol. Drug Des..

[126] Sikdar S., Mukherjee A., Khuda-Bukhsh A.R. (2015). Anti-Lung Cancer Potential of Pure Esteric-Glycoside Condurangogenin a against Non-Small-Cell Lung Cancer Cells in vitro via p21/p53-Mediated Cell Cycle Modulation and DNA Damage-Induced Apoptosis. Pharmacogn. Mag..

[127] Wang M., Tan J., Chen J., Xie T., Lin L.-M., Zhong L.-L., Cai X.-J., Wu Q.-S. (2021). Three New Ester Glycosides with Cytotoxic Activity from the Seeds of *Caesalpinia sappan*. Nat. Prod. Res..

[128] Chang H.K., Shin M.S., Yang H.Y., Lee J.W., Kim Y.S., Lee M.H., Kim J., Kim K.H., Kim C.J. (2006). Câncer de próstata e amigdalina (benzaldeído mais cianeto). Biol. Pharm. Bull..

[129] Milazzo S., Lejeune S., Ernst E. (2007). Laetrile for cancer: A Systematic Review of the Clinical Evidence. Support. Care Cancer.

[130] Meenatchi V., Narayanan K.B., Sood A., Han S.S. (2024). Formation of Amygdalin/β-Cyclodextrin Derivatives Inclusion Complexes for Anticancer Activity Assessment in Human Cervical Carcinoma HeLa Cell Line. Int. J. Pharm..

[131] Arshi A.S., Hosseini M., Hosseini F.S.K., Amiri Z.Y., Hosseini F.S., Sheikholia Lavasani M., Kerdarian H., Dehkordi M.S. (2019). The Anti-Cancer Effect of Amygdalin On Human Cancer Cell Lines. Mol. Biol. Rep..

[132] Nagdeve S.N., Suganthan B., Ramasamy R.P. (2025). Perspectives on the Application of Biosensors for the Early Detection of Oral Cancer. Sensors.

[133] Kuete V. (2014). Toxicological Survey of African Medicinal Plants.

[134] Yusuf U.F., Fakhru’l-Razi A.R., Ahmadun R.R., Iyuke S.E., Billa N., Abdullah N., Umar-Tsafe N. (2006). An in vitro Inhibition of Human Malignant Cell Growth of Crude Water Extract of Cassava (Manihot esculenta Crantz) and Commercial Linamarin. In Vitro.

[135] Idibie C.A., Davids H., Iyuke S.E. (2007). Cytotoxicity of Purified Cassava Linamarin to Selected Cancer Cell Lines. Bioprocess Biosyst. Eng..

[136] García-Escudero V., Gargini R. (2008). Autophagy Induction as an Efficient Strategy to Eradicate Tumors. Autophagy.

[137] Wang J., Luo L.Z., Liang D.M., Guo C., Huang Z.-H., Jian X.-H., Wen J. (2023). Recent Progress in Understanding Mitokines as Diagnostic and Therapeutic Targets in Hepatocellular Carcinoma. World J. Clin. Cases.

[138] Budzianowska A., Totoń E., Romaniuk-Drapała A., Kikowska M., Budzianowski J. (2023). Cytotoxic Effect of Phenylethanoid Glycosides Isolated from *Plantago lanceolata* L.. Life.

[139] Vinoth M., Natarajan B. (2021). Isolation, Characterization, Antibiological, Anticancer Activity of Tetrahydrobenzochromone Glycoside Derivative from Leaf of *Melochia corchorifolia*. Rasayan J. Chem..

[140] Zhu G.L., Hao Q., Xing L., Yang X.Q., Xie S.D., Zhao P., Li H.Z. (2021). C_21_, C_22_ Pregnane Glycosides and Cytotoxic C_27_ Spirostanol Steroids from Asparagus cochinchinensis. Steroids.

[141] Chauhan R., Malhotra L., Gupta A., Dagar G., Mendiratta M., Masoodi T., Hashem S., Al Marzooqi S., Das D., Uddin S. (2024). Bergenin Inhibits Growth of Human Cervical Cancer Cells by Decreasing Galectin-3 and MMP-9 Expression. Sci. Rep..

[142] Ngo T.H., Lee Y.J., Choi H., Song K.-S., Lee K.J., Nam J.-W. (2024). Evaluating the Anticancer Potential of *Polygonum multiflorum* Root-Derived Stilbenes Against H2452 Malignant Pleural Mesothelioma Cells. Fitoterapia.

[143] Gu J.P., Qi T.Z., Zhu D.R., He X.-J., Guo S.-P., Lan X., Gu H., Luo J.-L., Yang M., Gu Y.-C. (2025). Isolation of Pentasaccharide Resin Glycosides from the Whole Plants of Ipomoea biflora and Their Cytotoxic Activities. Phytochemistry.

[144] Janakiram N.B., Mohammed A., Rao C.V. (2015). Sea Cucumbers Metabolites as Potent Anti-Cancer Agents. Mar. Drugs.

[145] Barreca M., Spanò V., Montalbano A., Cueto M., Marrero A.R.D., Deniz I., Erdoğan A., Bilela L.L., Moulin C., Taffin-De-Givenchy E. (2020). Marine Anticancer Agents: An Overview with a Particular Focus on their Chemical Classes. Mar. Drugs.

[146] Gomes N.G., Dasari R., Chandra S., Kiss R., Kornienko A. (2016). Marine Invertebrate Metabolites with Anticancer Activities: Solutions to the “Supply Problem”. Mar. Drugs.

[147] Qu X.Y., Ren J.W., Peng A.H., Lin S.-Q., Lu D.-D., Du Q.-Q., Liu L., Li X., Li E.-W., Xie W.-D. (2019). Cytotoxic, Anti-Migration, and Anti-Invasion Activities on Breast Cancer Cells of Angucycline Glycosides Isolated from A Marine-Derived *Streptomyces* sp. Mar. Drugs.

[148] Bowers Z., Caraballo D., Bentley A. (2021). Therapeutic Potential of Pseudopterosin H on A Prostate Cancer Cell Line. J. Cancer Prev. Curr. Res..

[149] Hawas U.W., Abou El-Kassem L.T., Shaher F.M., Ghandourah M., Al-Farawati R. (2021). Sulfated triterpene glycosides from the Saudi Red Sea cucumber *Holothuria atra* with antioxidant and cytotoxic activities. Thalassas.

[150] Yang W.S., Qi X.R., Xu Q.Z., Yuan C.-H., Yi Y.-H., Tang H.-F., Shen L., Han H. (2021). A New Sulfated Triterpene Glycoside from the Sea Cucumber Colochirus quadrangularis, and Evaluation of Its Antifungal, Antitumor and Immunomodulatory Activities. Bioorg. Med. Chem..

[151] Anh C.V., Kwon J.H., Kang J.S., Lee H.-S., Heo C.-S., Shin H.J. (2022). New Angucycline Glycosides from A Marine-Derived Bacterium *Streptomyces ardesiacus*. Int. J. Mol. Sci..

[152] Silchenko A.S., Kalinovsky A.I., Avilov S.A., Kalinin V.I., Andrijaschenko P.V., Dmitrenok P.S., Chingizova E.A., Ermakova S.P., Malyarenko O.S., Dautova T.N. (2017). Nine New Triterpene Glycosides, Magnumosides A1–A4, B1, B2, C1, C2 and C4, from the Vietnamese Sea Cucumber *Neothyonidium* (=*Massinium*) *magnum*: Structures and Activities Against Tumor Cells Independently and in Synergy with Radioactive Irradiation. Mar. Drugs.

[153] Wu J., Yi Y.H., Tang H.F., Zou Z.-R., Wu H.-M. (2006). Structure and Cytotoxicity of a New Lanostane-Type Triterpene Glycoside from the Sea Cucumber *Holothuria hilla*. Chem. Biodivers..

[154] Zhang S.L., Li L., Yi Y.H., Sun P. (2006). Philinopsides E and F, Two New Sulfated Triterpene Glycosides from the Sea Cucumber Pentacta quadrangularis. Nat. Prod. Res..

[155] Zhang S.Y., Yi Y.H., Tang H.F. (2006). Bioactive Triterpene Glycosides from the Sea Cucumber *Holothuria fuscocinerea*. J. Nat. Prod..

[156] Dang N.H., Van N., Thanh P., Kiem V., Huong L.M., Minh C.V., Kim Y.H. (2007). Two New Triterpene Glycosides from the Vietnamese Sea Cucumber Holothuria scabra. Arch. Pharm. Res..

[157] Zou Z., Yi Y., Wu H., Yao X., Du L., Jiuhong W., Liaw C.C., Lee K.H. (2005). Intercedensides D–I, Cytotoxic Triterpene Glycosides from the Sea Cucumber *Mensamaria intercedens* Lampert. J. Nat. Prod..

[158] Yi Y.H., Xu Q.Z., Li L., Zhang S.L., Wu H.M., Ding J., Tong Y.G., Tan W.F., Li M.H., Tian F. (2006). Philinopsides A and B, Two New Sulfated Triterpene Glycosides from the Sea Cucumber *Pentacta quadrangularis*. Helv. Chim. Acta.

[159] Rodriguez J., Castro R., Riguera R. (1991). Holothurinosides: New Antitumour Non-Sulphated Triterpenoid Glycosides from the Sea Cucumber *Holothuria forskalii*. Tetrahedron.

[160] Liu B.S., Yi Y.H., Li L., Sun P., Yuan W., Sun G., Han H., Xue M. (2008). Argusides B and C, Two New Cytotoxic Triterpene Glycosides from the Sea Cucumber *Bohadschia argus* Jaeger. Chem. Biodivers..

[161] Lu Y., Li H., Wang M., Liu Y., Feng Y., Liu K., Tang H. (2018). Cytotoxic Polyhydroxysteroidal Glycosides from Starfish *Culcita novaeguineae*. Mar. Drugs.

[162] Li M., Miao Z.H., Chen Z., Chen Q., Gui M., Lin L.-P., Sun P., Yi Y.-H., Ding J. (2010). Echinoside A, A New Marine-Derived Anticancer Saponin, Targets Topoisomerase Iiα by Unique Interference with its DNA Binding and Catalytic Cycle. Ann. Oncol..

[163] Zhao Q., Xue Y., Wang J.F., Li H., Long T., Li Z., Wang Y., Dong P., Xue C. (2012). In vitro and in vivo Anti-Tumour Activities of Echinoside a And Ds-Echinoside A from Pearsonothuria graeffei. J. Sci. Food Agric..

[164] Wang J., Han H., Chen X., Yi Y., Sun H. (2014). Cytotoxic and Apoptosis-Inducing Activity of Triterpene Glycosides from *Holothuria scabra* and *Cucumaria frondosa* against HepG2 cells. Mar. Drugs.

[165] Malyarenko O.S., Dyshlovoy S.A., Kicha A.A., Ivanchina N.V., Malyarenko T.V., Carsten B., Gunhild V.A., Stonik V.A., Ermakova S.P. (2017). The Inhibitory Activity of Luzonicosides from the Starfish *Echinaster luzonicus* against human melanoma cells. Mar. Drugs.

[166] Park E.S., Yun S.H., Shin S.W., Kwak J.-Y., Park J.-I. (2012). Induction of Apoptosis and Antitumor Activity by Stichoposide D Through the Generation of Ceramide in Human Leukemia Cells. J. Life Sci..

[167] Yun S.H., Park E.S., Shin S.W., Na Y.-W., Han J.-Y., Jeong J.-S., Shastina V.V., Stonik V.A., Park J.-I., Kwak J.-Y. (2012). Stichoposide C Induces Apoptosis Through the Generation of Ceramide in Leukemia and Colorectal Cancer Cells and Shows in vivo Antitumor Activity. Clin. Cancer Res..

[168] Peng A., Qu X., Liu F., Li X., Li E., Xie W. (2018). Angucycline Glycosides from an Intertidal Sediment Strain *Streptomyces* sp. and Their Cytotoxic Activity Against Hepatoma Carcinoma Cells. Mar. Drugs.

[169] Sajwani F.H. (2019). Frondoside A is a Potential Anticancer Agent from Sea Cucumbers. J. Cancer Res. Ther..

[170] Zhang J.J., Zhu K.Q. (2017). A novel antitumor compound nobiliside D Isolated from Sea Cucumber (*Holothuria nobilis* Selenka). Exp. Ther. Med..

[171] Han H., Xu Q.Z., Yi Y.H., Gong W., Jiao B. (2010). Two New Cytotoxic Disulfated Holostane Glycosides from the Sea Cucumber *Pentacta quadrangularis*. Chem. Biodivers..

[172] Cuong N.X., Hoang L., Hanh T.T.H., Thao D.T., Van Thanh N., Nam N.H., Thung D.C., Van Kiem P., Van Minh C. (2017). Cytotoxic Triterpene Diglycosides from the Sea Cucumber *Stichopus horrens*. Bioorg. Med. Chem. Lett..

[173] Silchenko A.S., Avilov S.A., Kalinin V.I., Kalinovsky A.I., Dmitrenok P.S., Fedorov S.N., Stepanov V.G., Dong Z., Stonik V.A. (2008). Constituents of the Sea Cucumber *Cucumaria okhotensis*. Structures of Okhotosides B1–B3 and Cytotoxic Activities of Some Glycosides from this Species. J. Nat. Prod..

[174] Zhao Q., Xue Y., Liu Z.D., Li H., Wang J., Li Z., Wang Y., Dong P., Xue C. (2010). Differential Effects of Sulfated Triterpene Glycosides, Holothurin A1, and 24-Dehydroechinoside A, on Antimetastatic Activity Via Regulation of the MMP-9 Signaling Pathway. J. Food Sci..

[175] Chao C.H., Chou K.J., Huang C.Y., Wen Z.-H., Hsu C.-H., Wu Y.-C., Dai C.-F., Sheu J.-H. (2012). Steroids from the Soft Coral *Sinularia crassa*. Mar. Drugs.

[176] López-Sampedro E., Arreguin-Espinosa R., Simonet A.M. (2025). Cytotoxic Triterpene Glycosides from Mexican Sea Cucumber *Holothuria inornata*. J. Nat. Prod..

[177] Liu G., Zhang S., Lin R., Cao X., Yuan L. (2023). Anti-Tumor Target Screening of Sea Cucumber Saponin Frondoside A: A Bioinformatics and Molecular Docking Analysis. Front. Oncol..

[178] Silchenko A.S., Kalinovsky A.I., Avilov S.A., Popov R.S., Chingizova E.A., Menchinskaya E.S., Zelepuga E.A., Panina E.G., Stepanov V.G., Kalinin V.I. (2023). Sulfated Triterpene Glycosides from the Far Eastern Sea Cucumber *Cucumaria djakonovi*: Djakonoviosides C1, D1, E1, and F1; cytotoxicity against human breast cancer cell lines; quantitative structure–activity relationships. Mar. Drugs.

[179] Hieu N.V., Vinh L.B., Mai P.T., Hung L.N., Dat N.T., Phuong L.H., Anh T.P., Tuan D.T., Phong N.V., Hien T.T.T. (2025). Chemical Constituents from the Vietnamese Mangrove *Avicennia marina*: Two New Iridoid Glycosides and Their Cytotoxicity Against Cancer Cell Lines. Int. J. Mol. Sci..

[180] Liu D.N., Liu M., Zhang S.S., Shang Y.-F., Zhang W.-F., Song F.-H., Zhang H.-W., Du G.-H., Wang Y.-H. (2023). Chrysomycin A Regulates Proliferation and Apoptosis of Neuroglioma Cells via the Akt/GSK-3β Signaling Pathway in vivo and in vitro. Mar. Drugs.

[181] Kokkaliari S., Luo D., Paul V.J., Luesch H. (2023). Isolation and Biological Activity of Iezoside and Iezoside B, SERCA Inhibitors from Floridian Marine Cyanobacteria. Mar. Drugs.

[182] Silchenko A.S., Kalinovsky A.I., Avilov S.A., Popov R.S., Dmitrenok P.S., Chingizova E.A., Menchinskaya E.S., Panina E.G., Stepanov V.G., Kalinin V.I. (2023). Djakonoviosides A, A1, A2, B1–B4—Triterpene Monosulfated Tetra- and Pentaosides from the Sea Cucumber *Cucumaria djakonovi*: The first finding of a hemiketal fragment in the aglycones; activity against human breast cancer cell lines. Int. J. Mol. Sci..

[183] Malyarenko T.V., Kicha A.A., Kuzmich A.S., Malyarenko O.S., Kalinovsky A.I., Popov R.S., Dmitrenok P.S., Ivanchina N.V., Stonik V.A. (2024). New Rare Triterpene Glycosides from Pacific Sun Star, *Solaster pacificus*, and Their Anticancer Activity. Mar. Drugs.

[184] Silchenko A.S., Chingizova E.A., Menchinskaya E.S., Zelepuga E.A., Kalinovsky A.I., Avilov S.A., Tabakmakher K.M., Popov R.S., Dmitrenok P.S., Dautov S.S. (2024). Composition of Triterpene Glycosides of the Far Eastern Sea Cucumber *Cucumaria conicospermium* Levin et Stepanov; structure elucidation of five minor conicospermiumosides A3-1, A3-2, A3-3, A7-1, and A7-2; cytotoxicity of the glycosides against human breast cancer cell lines; structure–activity relationships. Mar. Drugs.

[185] Silchenko A.S., Kalinovsky A.I., Avilov S.A., Popov R.S., Chingizova E.A., Menchinskaya E.S., Zelepuga E.A., Tabakmakher K.M., Stepanov V.G., Kalinin V.I. (2024). The Composition of Triterpene Glycosides in the Sea Cucumber *Psolus peronii*: Anticancer Activity of the Glycosides Against Three Human Breast Cancer Cell Lines and Quantitative Structure–Activity Relationships (QSAR). Mar. Drugs.

[186] Kicha A.A., Tolkanov D.K., Malyarenko T.V., Malyarenko O.S., Kuzmich A.S., Kalinovsky A.I., Popov R.S., Stonik V.A., Ivanchina N.V., Dmitrenok P.S. (2024). Sulfated Polyhydroxysteroid Glycosides from the Sea of Okhotsk Starfish *Henricia leviuscula* Spiculifera and Potential Mechanisms for Their Observed Anti-Cancer Activity Against Several Types of Human Cancer Cells. Mar. Drugs.

[187] Zhang F., Wang W., Braun D.R., Ananiev G.E., Liao W., Harper M.K., Rajski S.R., Bugni T.S. (2025). Isolation, Structure Elucidation and Biological Evaluation of Lomaiviticins F–H, Dimeric Benzofluorene Glycosides from Marine-Derived *Micromonospora* sp. Bacterium. Mar. Drugs.

[188] Silchenko A.S., Zelepuga E.A., Chingizova E.A., Menchinskaya E.S., Tabakmakher K.M., Kalinovsky A.I., Avilov S.A., Popov R.S., Dmitrenok P.S., Kalinin V.I. (2025). Cladolosides of Groups S and T: Triterpene Glycosides from the Sea Cucumber *Cladolabes schmeltzii* with Unique Sulfation; Human Breast Cancer Cytotoxicity and QSAR. Mar. Drugs.

[189] Gucur G., Zeinali S., Gunay D., Nalbantsoy A., Sarikahya N.B. (2025). Cytotoxic Impacts of Holothurin B from Sea Cucumber *Holothuria tubulosa* Via Inducing Apoptosis on Human Prostate and Pancreatic Cancer Cells. Turk. J. Fish. Aquat. Sci..

[190] Dyshlovoy S.A., Hauschild J., Kriegs M., Hoffer K., Burenina O.Y., Strewinsky N., Malyarenko T.V., Kicha A.A., Ivanchina N.V., Stonik V.A. (2025). Anticancer Activity of Triterpene Glycosides from the Sea Star *Solaster pacificus*. Sci. Rep..

[191] Chou F.P., Hsu W.C., Huang S.C., Chang C.-Y., Chiou Y.-S., Tsai C.-T., Lyu J.W., Chen W.-T., Wu T.-K. (2020). Pregnenolonyl-α-glucoside exhibits marked anti-cancer and CYP17A1 enzymatic inhibitory activities. Chem. Commun..

[192] Krawczyk M., Pastuch-Gawolek G., Mrozek-Wilczkiewicz A., Kuczak M., Skonieczna M., Musiol R. (2019). Synthesis of 8-Hydroxyquinoline Glycoconjugates and Preliminary Assay of their β1,4-GalT Inhibitory and Anti-Cancer Properties. Bioorg. Chem..

[193] Tan Y., Xiao X., Yao J., Han F., Lou H., Luo H., Liang G., Ben-David Y., Pan W. (2017). Syntheses and Anti-Cancer Activities of Glycosylated Derivatives of Diosgenin. Chem. Res. Chin. Univ..

[194] Fujitaka Y., Hamada H., Masuoka N., Shimoda K., Uesugi D., Iwaki T., Kuboki A., Ishihara K., Kiriake Y. (2019). Chemo-Enzymatic Synthesis of Ester-Linked Docetaxel-Glycoside Conjugate and Its Drug Delivery System Using Hybrid-Bio-Nanocapsules Targeted with Trastuzumab and Cetuximab. Nat. Prod. Commun..

[195] Yu Y., Yu J., Zou X., Xu W., Zhang J., Bian H., Dong X., Shen Z. (2020). Studies on the Stereoselective Synthesis and Immunosuppressive Activity of Dihydroartemisinin-O-Glycoside Derivatives. Bioorg. Med. Chem. Lett..

[196] Rayavarapu S., Yarla N.S., Kadiri S.K., Bishayee A., Vidavalur S., Tadikonda R., Basha M., Pidugu V.R., Dowluru K.S.V.G.K., Lakappa D.B. (2017). Synthesis of Saccharumoside-B Analogue with Potential of Antiproliferative and Pro-Apoptotic Activities. Sci. Rep..

[197] Nguyen T.T.H., Pandey R.P., Parajuli P., Han J.M., Jung H.J., Park Y.I., Sohng J.K. (2018). Microbial Synthesis of Non-Natural Anthraquinone Glucosides Displaying Superior Antiproliferative Properties. Molecules.

[198] Sharipova R.R., Belenok M.G., Garifullin B.F., Sapunova A.S., Voloshina A.D., Andreeva O.V., Strobykina I.Y., Skvortsova P.V., Zuev Y.F., Kataev V.E. (2019). Synthesis and Anti-Cancer Activities of Glycosides and Glycoconjugates of Diterpenoid Isosteviol. MedChemComm.

[199] Elganzory H.H., Alminderej F.M., El-Bayaa M.N., Awad H.M., Nossier E.S., El-Sayed W.A. (2022). Design, Synthesis, Anticancer Activity and Molecular Docking of New 1,2,3-Triazole-Based Glycosides Bearing 1,3,4-Thiadiazolyl, Indolyl and Arylacetamide Scaffolds. Molecules.

[200] Xie J.Z., Xu H.J., Zhang Q., Wu Z., Wu X., Li X., Tan Z., Xie Y., Qiu L. (2023). Semi-Synthesis of Flavonoid Glycosides and Their Anti-Inflammatory and Antitumor Activities Towards Triple-Negative Breast Cancer. Chem. Biodivers..

[201] Liu M., Kong J.Q. (2018). The Enzymatic Biosynthesis of Acylated Steroidal Glycosides and Their Cytotoxic Activity. Acta Pharm. Sin. B.

[202] Gurung R.B., Gong S.Y., Dhakal D., Le T.T., Jung N.R., Jung H.J., Oh T.J., Sohng J.K. (2018). Synthesis of Curcumin Glycosides with Enhanced Anticancer Properties Using One-Pot Multienzyme Glycosylation Technique. J. Microbiol. Biotechnol..

[203] Zi C.T., Yang D., Dong F.W., Li G.-T., Li Y., Ding Z.-T., Zhou J., Jiang Z.-H., Hu J.-M. (2015). Synthesis and Antitumor Activity of Novel Per-Butyrylated Glycosides of Podophyllotoxin and Its Derivatives. Bioorg. Med. Chem..

[204] Alminderej F.M., Elganzory H.H., El-Bayaa M.N., Awad H.M., El-Sayed W.A. (2019). Synthesis and Cytotoxic Activity of New 1,3,4-Thiadiazole Thioglycosides and 1,2,3-Triazolyl-1,3,4-Thiadiazole N-Glycosides. Molecules.

[205] Khodair A.I., Alzahrani F.M., Awad M.K., Al-Issa S.A., Al-Hazmi G.H., Nafie M.S. (2023). Design, Synthesis, Computational Investigations, and Antitumor Evaluation of N-Rhodanine Glycoside Derivatives as Potent DNA Intercalation and Topo II Inhibition Agents Against Cancer Cells. ACS Omega.

[206] Srinivas A. (2021). Microwave-Assisted Synthesis of Hybrid Heterocyclics. Indian J. Chem..

[207] Vudhgiri S., Koude D., Veeragoni D.K., Misra S., Prasad R., Jala R.C.R. (2017). Synthesis and Biological Evaluation of 5-Fatty-Acylamido-1,3,4-Thiadiazole-2-Thioglycosides. Bioorg. Med. Chem. Lett..

[208] Flefel E.M., El-Sayed W.A., Mohamed A.M., El-Sofany W.I., Awad H.M. (2017). Synthesis and Anticancer Activity of New 1-Thia-4-azaspiro[4.5]Decane, their Derived Thiazolopyrimidine and 1,3,4-Thiadiazole Thioglycosides. Molecules.

[209] Khalaf H., Tolan H., El-Bayaa M., Radwan M.A.A., El-Manawaty M., El-Sayed W.A. (2020). Synthesis and Anticancer Activity of New Pyridine-Thiophene and Pyridine-Furan Hybrid Compounds, Their Sugar Hydrazone, and Glycosyl Derivatives. Russ. J. Gen. Chem..

[210] Khodair A.I., Alsafi M.A., Nafie M.S. (2019). Synthesis, Molecular Modeling and Anti-Cancer Evaluation of a Series of Quinazoline Derivatives. Carbohydr. Res..

[211] Khodair A.I., Attia A.M., Gendy E.A., Elshaier Y.A.M.M., El-Magd M.A. (2019). Discovery of New S-Glycosides and N-Glycosides of Pyridine-Biphenyl System with Antiviral Activity and Induction of Apoptosis in MCF-7 Cells. J. Heterocycl. Chem..

[212] Tolan H.E., Radwan M.A., Soliman H.A., Awad H.M., El-Sayed W.A. (2020). Synthesis and Anti-Proliferative Activity of New Acridinyl and Benzothiazolyl-Based Triazole Glycosides Via Click Cycloaddition and Their Tetrazolyl Analogs. Russ. J. Bioorg. Chem..

[213] Khalaf H.S., Tolan H.E., Radwan M.A., Mohamed A.M., Awad H.M., El-Sayed W.A. (2020). Design, Synthesis and Anticancer Activity of Novel Pyrimidine and Pyrimidine-Thiadiazole Hybrid Glycosides. Nucleosides Nucleotides Nucleic Acids.

[214] El-Sofany W.I., El-Sayed W.A., Abd-Rabou A.A., El-Shahat M. (2022). Synthesis of New Imidazole-Triazole-Glycoside Hybrids as Anti-Breast Cancer Candidates. J. Mol. Struct..

[215] El-Sayed W.A., Alminderej F.M., Mounier M.M., Nossier E.S., Saleh S.M., Kassem A.F. (2022). New 1,2,3-Triazole-Coumarin-Glycoside Hybrids and Their 1,2,4-Triazolyl Thioglycoside Analogs Targeting Mitochondria Apoptotic Pathway: Synthesis, Anticancer Activity and Docking Simulation. Molecules.

[216] Libnow S., Methling K., Hein M., Michalik D., Harms M., Wende K., Flemming A., Köckerling M., Reinke H., Bednarski P.J. (2008). Synthesis of Indirubin-N′-glycosides and their Anti-Proliferative Activity Against Human Cancer Cell Lines. Bioorg. Med. Chem..

[217] Tiwari G., Mishra V.K., Kumari P., Khanna A., Sharma S., Sagar R. (2024). Synthesis of Triazole Bridged N-Glycosides of Pyrazolo[1,5-α]Pyrimidinones as Anticancer Agents and their in silico Docking Studies. RSC Adv..

[218] Moreno-Narváez M.E., Arenaza-Corona A., González-Sebastián L., Ramírez T.A., Ortega S.H., Cruz-Navarro J.A., Alí-Torres J., Orjuela A.L., Reyes-Marquez V., Lomas-Romero L. (2025). Glycoconjugate Pd(II) and Cu(II) Complexes of Fluorinated N,O Schiff Base Ligands for Targeted Cancer Therapy: Synthesis, Characterization and in vitro Cytotoxic Activity Evaluation. New J. Chem..

[219] Abbas H.A.S., Nossier E.S., El-Manawaty M.A., El-Bayaa M.N. (2024). New Sulfonamide-Based Glycosides Incorporated 1,2,3-Triazole as Cytotoxic Agents Through VEGFR-2 and Carbonic Anhydrase Inhibitory Activity. Sci. Rep..

[220] Srour A.M., El-Bayaa M.N., Temirak A., Alanzy A.L., Awad H.M., Saleh A., Saleh M.G., El-Sayed W.A. (2025). New Benzimidazole-Triazole Glycoconjugates as Anti-Cancer Agents and EGFR Inhibitors. Sci. Rep..

[221] Somani S., Menta L., Wang-Johanning F., Johanning G., Hong S., Njoo E. (2025). Synthesis, Anticancer Properties, And Biological Profiling of Synthetic Glycan Analogs of Proscillaridin A. Discov. Pharm. Sci..

[222] Kassem A.F., Omar M.A., Nossier E.S., Awad H.M., El-Sayed W.A. (2023). Novel Pyridine-Thiazolidinone-Triazole Hybrid Glycosides Targeting EGFR and CDK-2: Design, Synthesis, Anticancer Evaluation, and Molecular Docking Simulation. J. Mol. Struct..

[223] El Malah T., El-Bayaa M.N., Abdelrahman M.T., Awad H.M., Soliman H.A. (2025). Synthesis of Some 1,2,3-Triazole-Bridged Glycosides with Benzoquinoline-3-Carbonitriles via Click Chemistry for Anticancer and Docking Evaluation. J. Mol. Struct..

[224] Kassem A.F., Younis A., Nossier E.S., Awad H.M., El-Sayed W.A. (2024). Pyridine-Based Glycosides Bearing 1,2,4-Triazole and Their 1,3,4-Oxadiazole Analogues as Potential EGFR and CDK-2 Inhibitors: Design, Synthesis, Antiproliferative Activity and in silico Studies. J. Mol. Struct..

