# Supplementary material for: Exploring the Anticancer Potential of Plant, Marine, and Synthetic Glycosides: A Comprehensive Review
Source: Biomolecules. 2026 Oct 14;16(10):1493. doi: 10.3390/biom16101493 (PMC13702531; doi:10.3390/biom16101493)
Supplement: Supplementary file 1 [file biomolecules-16-01493-s001.zip › biomolecules-4521187-supplementary.pdf]

Supplementary Table S1

Exploring the Anticancer Potential of Plant, Marine, and Synthetic Glycosides: A Comprehensive Review

Tooba Jabeen[1], Sumayya Akram[1], Matloob Ahmad[1], Sami A. Al-Hussain[2], and Magdi E. A. Zaki[2]

[1] Department of Chemistry, Government College University, Faisalabad 38000, Pakistan; sumayya.201002384@gcuf.edu.pk
[2] Department of Chemistry, Faculty of Science, Imam Mohammad Ibn Saud Islamic University (IMSIU), Riyadh 11623, Saudi Arabia; mezaki@imamu.edu.sa

Correspondence: matloob.ahmad@gcuf.edu.pk (M.A.); sahussain@imamu.edu.sa (S.A.A.H.)

Supplementary Table S1. Comprehensive IC_{50} Data for Plant-Based and Synthetic Glycosides

Plant-Derived Glycosides (Steroid Glycosides)

**Compound(s) / Extract**	**Cell Line / Target**	**IC_{50} (or GI_{50}) Value**	**SD/CI Reported?**	**Ref.**
Digitoxin (4)	NSCLC H1975	CC_{50} = 0.19 ± 0.06 μM	Y	[39]
Oleandrin (5)	SW480 (colon)	IC_{50} = 0.02 μM	N	[40]
Oleandrin (5)	Normal colonic epithelial cells	IC_{50} = 0.56 μM	N	[40]
Compounds 9–14 (undescribed cardiac glycosides)	A549	IC_{50} = 0.01–6.08 μM (range)	N	[46]
α-L-diginoside (18)	OSCC cell lines	IC_{50} < 0.2 μM	N	[50]
Proscillaridin A (19, extract)	Caco-2	IC_{50} = 0.9 μg/mL	N	[52]
Proscillaridin A (19, extract)	COLO-205	IC_{50} = 2.3 μg/mL	N	[52]
Proscillaridin A (19, extract)	Normal fibroblasts	IC_{50} = 13.1 μg/mL	N	[52]
Calotropin (24) / Calotoxin (25)	MDA-MB-231, A549	IC_{50} = 0.046–0.072 μM (vs. reference 0.47–58.41 μM)	N	[57]
Solamargine (30)	NIH-3T3 fibroblast	IC_{50} = 7.55 ± 1.5 μg/mL	Y	[62]
Dioscine (31)	NIH-3T3 fibroblast	IC_{50} = 3.3 ± 1.9 μg/mL	Y	[62]
Cycloheximide (reference)	NIH-3T3 fibroblast	IC_{50} = 0.8 ± 0.2 μg/mL	Y	[62]
β-Sitosterol glycoside (36)	MCF-7	IC_{50} = 24.83 μg/mL	N	[66]

Plant-Derived Glycosides (Flavonoid and Polyphenol Glycosides)

**Compound(s) / Extract**	**Cell Line / Target**	**IC_{50} (or GI_{50}) Value**	**SD/CI Reported?**	**Ref.**
(−)-Epigallocatechin-3-O-gallate deriv. (44)	CAL27	IC_{50} = 9.78 ± 0.25 μM	Y	[68]
(−)-Epigallocatechin-3-O-gallate deriv. (45)	MDA-MB-231	IC_{50} = 3.27 ± 0.18 μM	Y	[68]
Capecitabine (reference)	CAL27	IC_{50} = 8.20 ± 0.75 μM	Y	[68]
Capecitabine (reference)	MDA-MB-231	IC_{50} = 5.20 ± 0.89 μM	Y	[68]
Isorhamnetin glycosides (49–53)	HepG2 / MCF-7 / A549 / HCT-116	IC_{50} = 2.6, 10.9, 37.9, 5.4 mg/mL (per line)	N	[71]
Ethyl acetate fraction (quercetin glycosides 55, 56)	T47D	IC_{50} = 147.90 ± 8.49 mg/mL	Y	[74]
Tiliroside-containing extract	h-TOP-IIβ (enzyme)	IC_{50} = 45.05 ng/mL	N	[75]
Staurosporine (reference)	h-TOP-IIβ (enzyme)	IC_{50} = 135.33 ng/mL	N	[75]
Tiliroside-containing extract	HCT-116	IC_{50} = 3.19 μg/mL	N	[75]
Tiliroside-containing extract	MCF-7	IC_{50} = 3.47 μg/mL	N	[75]
Tiliroside-containing extract	HepG2	IC_{50} = 2.27 μg/mL	N	[75]
Naringenin-7-O-glucoside (67)	MDA-MB-231, MCF-7	IC_{50} = 233.56 μg/μL	N	[94]
Compound 71	MDA-MB-231	IC_{50} = 155.21 ± 1.59 μM	Y	[97]
Colvillea racemosa extract (leaf)	A549	IC_{50} = 17.0 μg/mL	N	[100]
Colvillea racemosa extract (flower)	A549	IC_{50} = 17.2 μg/mL	N	[100]
Vicenin-II (83)	HepG2	IC_{50} = 14.38 μg/mL	N	[102]
Isovitexin (84)	HepG2	IC_{50} = 15.39 μg/mL	N	[102]
Methylene chloride fraction (L. hispidulus)	PC3	IC_{50} = 19 μg/mL	N	[103]
Ethyl acetate fraction (L. hispidulus)	PC3	IC_{50} = 19.6 μg/mL	N	[103]
Compound 91	A2780	IC_{50} = 77.0 μg/mL	N	[104]

Taxol (reference)	A2780	IC_{50} = 76.9 μg/mL	N	[104]

Plant-Derived Glycosides (Terpene and Terpenoid Glycosides)

Compound(s) / Extract	Cell Line / Target	IC_{50} (or GI_{50}) Value	SD/CI Reported?	Ref.
Compounds 92, 93	MCF-7 / MDA-MB-231 / MDA-MB-435s (XTT assay)	IC_{50} = 120.73–154.01 μg/mL (range)	N	[105]
Compounds 95a, 95b, 96, 97	A2058, HT29, A549	IC_{50} = 2.01 ± 0.17 to 18.8 ± 1.17 μM (range)	Y	[109]
Compound 95b (narcissiflorin)	HT29	IC_{50} = 2.02 ± 1.65 μM	Y	[109]
Compound 95b (narcissiflorin)	A549	IC_{50} = 2.01 ± 0.17 μM	Y	[109]
Irinotecan hydrochloride (reference)	HT29	IC_{50} = 10.3 ± 1.32 μM	Y	[109]
Irinotecan hydrochloride (reference)	A549	IC_{50} = 9.89 ± 0.19 μM	Y	[109]
Premna barbata stem bark extract (contains geniposidic acid, 128)	COLO-205	GI_{50} ≈ 44.6 μg/mL (value as reported; variance formatting unclear in source)	Unclear	[77]

Plant-Derived Glycosides (Other Glycosides)

Compound(s) / Extract	Cell Line / Target	IC_{50} (or GI_{50}) Value	SD/CI Reported?	Ref.
Condurangogenin A (129)	H460	IC_{50} = 32 μg/mL	N	[126]
Compound 141	MCF-7	IC_{50} = 120.71 ± 3.46 (unit not restated at this point in source)	Y	[139]
Compounds 142, 143	Cervical cancer cell lines	IC_{50} = 35.5 and 39.6 μM	N	[140]

Synthetic Glycosides (O-Glycosides)

Compound(s) / Extract	Cell Line / Target	IC_{50} (or GI_{50}) Value	SD/CI Reported?	Ref.
Compounds 330 (curcumin glycoside)	AGS	IC_{50} = 7.11 μM	N	[202]
Compounds 330 (curcumin glycoside)	HCT116	IC_{50} = 5.27 μM	N	[202]
Compound 332 (curcumin glycoside)	AGS	IC_{50} = 5.86 μM	N	[202]
Compound 332 (curcumin glycoside)	HepG2	IC_{50} = 18.61 μM	N	[202]
Compound 332 (curcumin glycoside)	HCT	IC_{50} = 5.40 μM	N	[202]
Compound 332 (curcumin glycoside)	HeLa	IC_{50} = 12.47 μM	N	[202]
Compounds 338b, 338e, 342 (podophyllotoxin glycosides)	SMMC-7721, A-549, MCF-7, HL-60, SW480 (panel)	IC_{50} = 0.14–1.69 μM (range)	N	[203]

Synthetic Glycosides (N-Glycosides)

Compound(s) / Extract	Cell Line / Target	IC_{50} (or GI_{50}) Value	SD/CI Reported?	Ref.
Compound 397a (quinazoline S-glycoside)	MCF-7	IC_{50} = 2.09 μM	N	[210]
Compound 397a (quinazoline S-glycoside)	HepG2	IC_{50} = 2.08 μM	N	[210]

Synthetic Glycosides (S-Glycosides)

Compound(s) / Extract	Cell Line / Target	IC_{50} (or GI_{50}) Value	SD/CI Reported?	Ref.
Compound 358a (N-rhodanine glycoside)	HepG2, MCF-7, A549	IC_{50} = 3.7, 8.2, 9.8 μM (per line)	N	[205]
Compound 359b (N-rhodanine glycoside)	HepG2, MCF-7, A549	IC_{50} = 3.1, 13.7, 21.8 μM (per line)	N	[205]

Compound 359f (N-rhodanine glycoside)	HepG2, MCF-7, A549	IC_{50} = 7.17, 2.2, 4.5 μM (per line)	N	[205]
Doxorubicin (reference)	HepG2, MCF-7, A549	IC_{50} = 7.67, 8.28, 6.62 μM (per line)	N	[205]
Compound 359f	DNA intercalation assay	IC_{50} = 7.3 μM	N	[205]
Compound 359f	Topo II inhibition assay	IC_{50} = 18.2 μM	N	[205]
Doxorubicin (reference, 359f comparator)	DNA intercalation / Topo II	IC_{50} = 9.65 and 31.27 μM	N	[205]
Thioglycoside derivatives (371a-j, 372a-j)	HCT116 (most active subset)	IC_{50} = 92.2–120.1 nM (range)	N	[208]

Synthetic Glycosides (Miscellaneous)

**Compound(s) / Extract**	**Cell Line / Target**	**IC_{50} (or GI_{50}) Value**	**SD/CI Reported?**	**Ref.**
Compounds 449b, 449d (coumarin triazole-glycosyl hybrids)	vs. Sorafenib/Erlotinib/Roscovitine targets	IC_{50} = 0.22±0.01 / 0.93±0.42 / 0.24±0.20 μM (449b); 0.12±0.50 / 0.79±0.14 / 0.15±0.60 μM (449d)	Y	[215]
Sorafenib / Erlotinib / Roscovitine (references)	Same targets	IC_{50} = 1.58±0.11 / 0.18±0.05 / 0.46±0.30 μM	Y	[215]
Compounds 460a-l, 461a-l (isoxazole/pyrazoline indole glycosides)	MDA-MB-231, MCF-7, MDA-MB-453	IC_{50} = 0.67–4.67 μM (range, active subset)	N	[216]
Compound 464b (triazole-bridged N-glycoside)	MDA-MB-231	IC_{50} = 27.66 mM	N	[217]
Compound 466f (triazole-bridged N-glycoside)	MCF-7	IC_{50} = 4.93 mM	N	[217]
Compounds 474a, 474b (sulfonamide-triazole-glycoside hybrids)	HepG-2	IC_{50} = 8.39–16.90 μM (range)	N	[219]
Compounds 474a, 474b	MCF-7	IC_{50} = 19.57–21.15 μM (range)	N	[219]
Doxorubicin (reference)	HepG-2	IC_{50} = 13.76 ± 0.45 μM	Y	[219]
Doxorubicin (reference)	MCF-7	IC_{50} = 17.44 ± 0.46 μM	Y	[219]

Compounds 474a, 474b	VEGFR-2 (enzyme)	IC_{50} = 1.33 and 0.38 μM	N	[219]
Compounds 474a, 474b	hCA IX (enzyme)	IC_{50} = 66 and 40 nM	N	[219]
Compounds 474a, 474b	hCA XII (enzyme)	IC_{50} = 7.6 and 3.2 nM	N	[219]
Sorafenib (reference)	hCA IX (enzyme)	IC_{50} = 0.43 μM	N	[219]
SLC-0111 (reference)	hCA IX / hCA XII (enzyme)	IC_{50} = 53 and 4.8 nM	N	[219]
Compound 479a (N-glycosyl heterocyclic)	HepG-2	IC_{50} = 1.64 ± 0.11 μM	Y	[220]
Compound 479a	HCT-116	IC_{50} = 5.00 ± 0.51 μM	Y	[220]
Erlotinib (reference)	HepG-2 / HCT-116	IC_{50} = 2.07 ± 0.07 and 5.14 ± 0.33 μM	Y	[220]
Compounds 479a, 479b	EGFR (enzyme)	IC_{50} = 0.069 ± 0.003 and 0.075 ± 0.003 μM	Y	[220]
Erlotinib (reference)	EGFR (enzyme)	IC_{50} = 0.048 ± 0.002 μM	Y	[220]
Compounds 491a-c (pyridine-thiazole-triazole glycosides)	CDK-2/cyclin A2 (enzyme)	IC_{50} = 0.26, 0.22, 0.18 μM (per compound)	N	[222]
Roscovitine (reference)	CDK-2/cyclin A2 (enzyme)	IC_{50} = 0.42 μM	N	[222]
Compounds 491b, 491c	EGFR (enzyme)	IC_{50} = 0.12 and 0.15 μM	N	[222]
Erlotinib (reference)	EGFR (enzyme)	IC_{50} = 0.15 μM	N	[222]
Compounds 499, 503 (pyridine-triazole / oxadiazole glycosides)	HepG-2	IC_{50} = 10.6 and 12.8 μM	N	[224]
Compounds 499, 503	MCF-7	IC_{50} = 8.3 and 9.8 μM	N	[224]
